# *Microcystis* pangenome reveals cryptic diversity within and across morphospecies

**DOI:** 10.1126/sciadv.add3783

**Published:** 2023-01-13

**Authors:** Haiyuan Cai, Christopher J. McLimans, Jessica E. Beyer, Lee R. Krumholz, K. David Hambright

**Affiliations:** ^1^ Plankton Ecology and Limnology Laboratory, Department of Biology, University of Oklahoma, Norman, OK, USA.; ^2^ Program in Ecology and Evolutionary Biology, Department of Biology, University of Oklahoma, Norman, OK, USA.; ^3^ Department of Microbiology and Plant Biology and Institute for Energy and the Environment, University of Oklahoma, Norman, OK, USA.; ^4^ Geographical Ecology, Department of Biology, University of Oklahoma, Norman, OK, USA.

## Abstract

*Microcystis*, a common harmful algal bloom (HAB) taxon, threatens water supplies and human health, yet species delimitation is contentious in this taxon, leading to challenges in research and management of this threat. Historical and common morphology-based classifications recognize multiple morphospecies, most with variable and diverse ecologies, while DNA sequence–based classifications indicate a single species with multiple ecotypes. To better delimit *Microcystis* species, we conducted a pangenome analysis of 122 genomes. Core- and non–core gene phylogenetic analyses placed 113 genomes into 23 monophyletic clusters containing at least two genomes. Overall, genome-related indices revealed that *Microcystis* contains at least 16 putative genospecies. Fifteen genospecies included at least one *Microcystis aeruginosa* morphospecies, and 10 genospecies included two or more morphospecies. This classification system will enable consistent taxonomic identification of *Microcystis* and thereby aid in resolving some of the complexities and controversies that have long characterized eco-evolutionary research and management of this important HAB taxon.

## Teaser

Cryptic diversity in Microcystis may explain ecotype variability across morphospecies.

## INTRODUCTION

Ecologically, the widespread, toxigenic, bloom-forming, ecosystem-disruptive cyanobacterium *Microcystis* represents an intriguing enigma, due to its complex and controversial taxonomy (*1*), combined with its characteristically broad niche and cosmopolitan distribution (*2*–*5*). Variations in nutrient affinities, uptake rates, cell quotas, nitrogen metabolism, and toxin production have been observed within species (*4*, *6*, *7*), leading to the question of whether current morphological taxonomy represents accurate and meaningful species delimitation that can reliably inform *Microcystis* evolutionary history and niche adaptation. This enigma is typified by the most common species, *Microcystis aeruginosa*, but is also apparent in others, including *Microcystis viridis*, *Microcystis wesenbergii*, *Microcystis ichthyoblabe*, *Microcystis natans*, *Microcystis botrys*, *Microcystis firma*, *Microcystis flos-aquae*, *Microcystis novacekii*, *Microcystis panniformis*, and *Microcystis smithii* [e.g., (*8*, *9*)]. Colony and cell morphology, primary characteristics used for species delineation, and toxin production, the primary public health concern for temperate and tropical waters globally, have been shown to vary within *Microcystis* taxa and across environmental and seasonal conditions (*4*, *5*, *10*–*15*).

Taxonomy and systematics provide a foundational understanding of the biology and evolutionary relationships among organisms, without which our understanding of an organism’s biology and ecology is severely impeded (*16*, *17*). Historically, taxonomy has been predominantly morphology based, although morphology does not necessarily reflect species boundaries as defined genetically (*16*, *18*). Working with a flawed species delineation will inevitably produce erroneous answers to evolutionary and biogeographical questions. For example, the whole-genome–based analysis of *Prochlorococcus* by Thompson and colleagues (*19*) upended the previous assumption of niche specialization and range restriction. On the basis of whole genomes, their analysis revealed that some species are generalists with wide geographical ranges, whereas some are specialists with narrow ranges. Regardless of which species concept is used (*20*, *21*), limitations of morphologically based taxonomy can become exacerbated by the low morphological complexity and small size of microbes (*16*). For example, it has long been known that morphology is unreliable for cyanobacterial species identification and systematics (*9*, *22*, *23*). *Microcystis* is no different. Because of the morphological and ecological incongruencies across *Microcystis* species, researchers have sought and used genetic-based taxonomic markers for decades [e.g., (*24*, *25*)].

Standard genetic markers in bacterial taxonomy include hypervariable regions within the 16*S* small subunit ribosomal RNA [rRNA; sensu, (*26*)], but the utility of the 16*S* rRNA genes for taxonomic resolution at the species level within *Microcystis* has proven uninformative due to high sequence similarities (>>97%) (*4*, *5*, *27*). Intergenic regions with higher levels of sequence divergence than 16*S* hypervariable regions, including the internal transcribed spacer (ITS) of the rRNA operon (*28*–*30*) and the intergenic spacer genes for phycocyanin biosynthesis (cpcBA-IGS) (*28*, *31*–*33*), have also previously been used in an effort to analyze *Microcystis* diversity and composition. As with 16*S* rRNA genes, these markers have proven insufficient with respect to satisfactory taxonomic or phylogenetic resolution beyond a single *Microcystis* genus. Classification using multilocus sequence typing based on seven single-copy housekeeping genes has been useful in sorting species belonging to this genus (*34*); however, the selected genes might not reflect overall genome evolution of *Microcystis.*

Whole-genome sequencing provides a potential solution to the lack of taxonomic resolution of traditional genetic markers (*19*, *35*, *36*). In instances for which full-length 16*S* rRNA sequence similarity is ≥98.7%, Chun *et al.* (*37*) suggest that species boundaries might be identified using whole-genome sequencing and an overall genome-related index (OGRI) based on average nucleotide identity (ANI) of <95 to 96% and digital DNA-DNA hybridization (dDDH) values of <70%. However, studies have shown that multiple *Microcystis* morphospecies form a single-species complex using this approach (*4*, *5*, *9*). Moreover, such universal criteria may not be robust for taxa with high variability in genome size and gene content, such as *Microcystis*, in which only a small part of each genome contains appropriate orthologous regions used in ANI calculations. Such high genome variability is primarily due to high genome plasticity and horizontal gene transfer (HGT), which has apparently played a substantial role in the evolution of *Microcystis* (*12*–*14*, *38*, *39*). As a complement to OGRI-based analyses, the use of a small group of vertically inherited genes (core genes) has the potential to create a more complete genealogical history in difficult to classify families of bacteria (*40*). Moreover, use of single-copy orthologous genes could minimize confounding effects of HGT (*41*) and thereby generate robust and resolved phylogenies (*42*). Further, phylogenies based on the complete genome data (i.e., core and noncore genes) should reflect both an organism’s evolution and adaptation to specific habitats, thus providing a preferable framework for demarcating species (*40*, *43*).

Here, we used 122 published *Microcystis* genomes to create a robust phylogeny based on both core- and pan-gene phylogenies and identified putative genospecies using modified OGRI thresholds. We classified 113 genomes into 23 monophyletic clusters and identified at least 16 putative genospecies. To aid in the application of this new classification scheme when whole-genome sequences are not available, we propose 11 marker genes that can reliably place new *Microcystis* strains within the classification scheme. Ultimately, this new and emerging *Microcystis* taxonomy will focus further research on better characterizing *Microcystis* ecotypes, niches, and evolutionary history.

## RESULTS

### The *Microcystis* pangenome

**Fig. 1.**
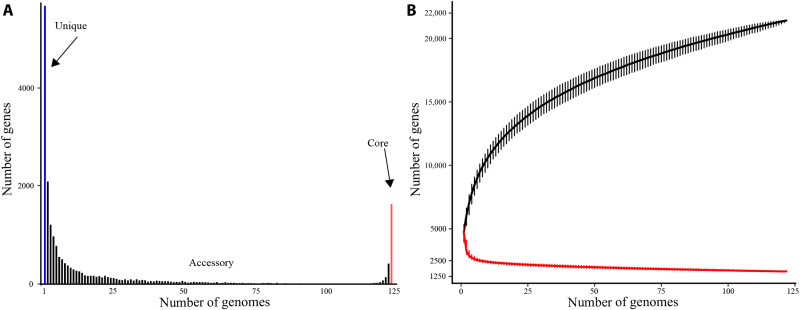
Pangenome analysis of 122 *Microcystis*. (**A**) The frequencies of pan-genes (*y* axis) are plotted as a function of the number of genomes sharing those genes (*x* axis). Blue indicates unique genes, black indicates accessory genes, and orange indicates core genes. (**B**) Rarefaction curves created with PEPPAN for the accumulations of pan genes and core genes of 122 *Microcystis* genomes from 1000 random permutations. The gene accumulation curve of the increasing number of genomes fitted the power law (*n* = κN^α^) with exponent α ± 95% confidence interval (CI) = 0.847 ± 0.003; core genes decreased as the number of genomes increases and followed a power law (*n* = κN^α^) with exponent α ± 95% CI = −0.172 ± 0.008. Error bars indicate 95% CIs for 1000 random permutations.

The 122 *Microcystis* genomes ranged in size between 3.88 and 5.89 million base pairs (Mbp) and were comprised on average of 354 contigs, 42.7% G + C content, and 4597 protein-coding sequences (table S1). Inclusion of potentially incomplete draft genomes in our analysis did not appear to affect these estimates, as the nine complete genomes ranged in size from 4.30 to 5.87 Mbp, with an average of 42.5% G + C content, and 4999 protein-coding sequences. The pangenome contained 21,880 nonredundant genes, of which the core, accessory, and unique genomes were composed of 1639, 14,562, and 5679 genes, respectively (Fig. 1A). The rarefaction curve showed that more genes would be identified as more genomes are added, but the rate of new gene discovery decelerates as more genomes are added (43 new genes are predicted for the 123rd genome) (Fig. 1B). The rate of reduction in core gene numbers also slows but is projected to be much less affected by the addition of new genomes (e.g., the 123rd genome would have ~two fewer core genes) (Fig. 1B).

Among the core genes, 1452 single-copy genes (table S2) were used to identify core genomic relationships and evolutionary history. These single-copy genes included B vitamin biosynthetic genes, such as *thiCEG* [thiamine (B_1_)-phosphate synthase], *ribAB* [riboflavin (B_2_) biosynthesis], *pdxJ* [pyridoxine (B_6_) 5′-phosphate synthase], and *cobD* [cobalamin (B_12_) biosynthesis]. A set of organic and inorganic compound transporter genes were also present, including *pstABC* (phosphate transport system), *amtB* (an ammonium transporter), *cmpBCD* (bicarbonate transport system), *cysTW* (sulfate transport system), *corA* (cobalt/magnesium transport protein), *znuABC* (high-affinity zinc uptake system), *dppB* (dipeptide transport system permease protein), *glnQ* (glutamine transport adenosine triphosphate–binding protein), and *livFH* (high-affinity branched-chain amino acid transport system).

### *Microcystis* phylogeny

**Fig. 2.**
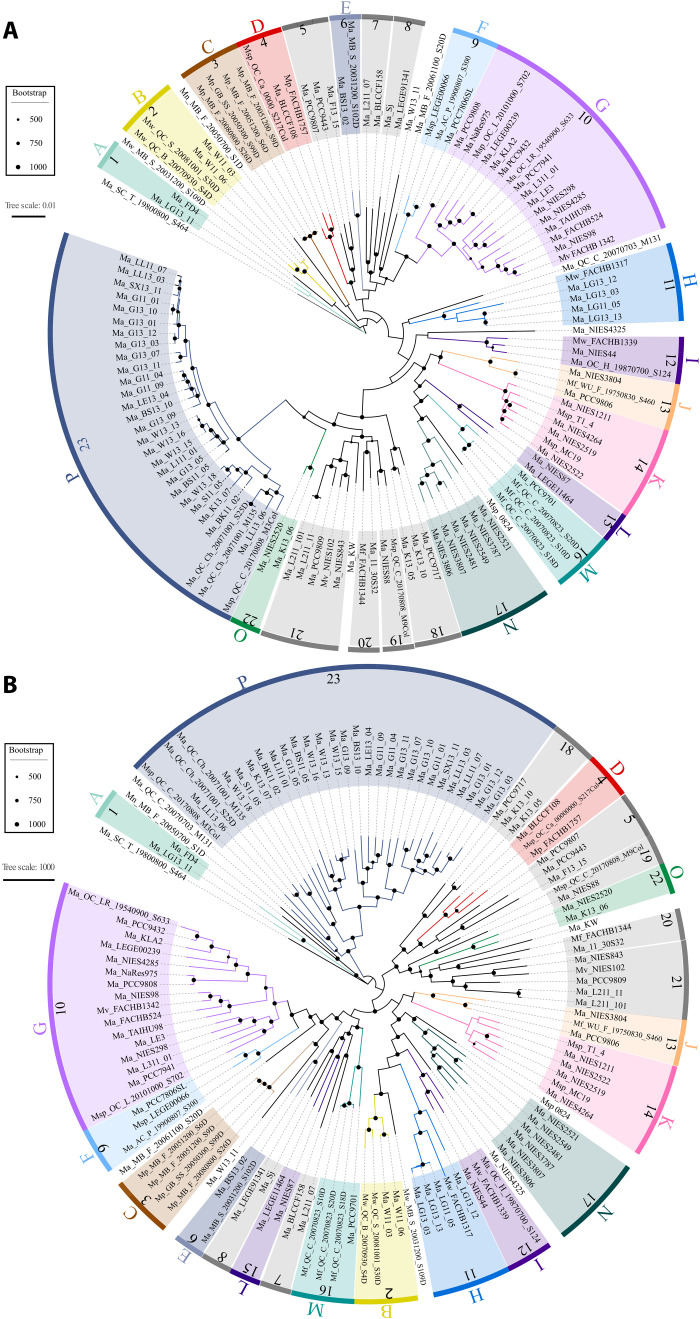
*Microcystis* core- and pan-gene phylogenies. Twenty-three genomically coherent, monophyletic clusters identified by comparing core phylogeny and pangenome-based dDDH/ANI clustering. (**A**) Rooted maximum likelihood phylogenetic tree inferred for 122 *Microcystis* genomes using the concatenated alignment of 1452 single-copy core genes. A total of 1,330,000 nucleotide positions was used, and *M. aeruginosa* Ma_SC_T_19800800_S464 was used as an outgroup, as identified through a previous comparison of all *Microcystis* genomes to other cyanobacteria (see fig. S1). (**B**) Phylogeny based on the binary gene presence and absence matrix of all pan-genes. Bootstrap support values were calculated from 1000 replicates; values above 500 are indicated. Lettered color segments indicate genospecies supported by ANI and dDDH clustering; numbered gray segments indicate clusters that were not supported by ANI and dDDH.

The phylogeny based on sequence similarity of the single-copy core genes suggested the existence of 23 conserved, monophyletic clusters containing 113 genomes (Fig. 2A). Nine genomes, including the outgroup, failed to group with any other genome. The presence of 23 single-copy core gene clusters was corroborated by the pan-gene presence-absence tree (Fig. 2B). There were some differences in topology between the single-copy core and pan-gene trees, but they had identical cluster membership, including the same nine outliers. All 23 clusters included at least two genomes, were monophyletic, genomically coherent across core- and pan-gene phylogenies, and were supported by high bootstrap values. Moreover, ANI and dDDH analyses (fig. S2) revealed that 16 of the 23 clusters could be defined by thresholds of 0.970 and 0.750, respectively, with maximum between cluster ANI and dDDH values of 0.969 and 0.746, respectively (table S3).

Twenty-two of 23 clusters and 15 of the 16 putative genospecies contained at least one genome previously described as *M. aeruginosa* [as identified in the National Center for Biotechnology Information (NCBI) database]. Twelve clusters and 10 genospecies included at least two morphospecies. Genospecies C was composed solely of *M. panniformis*. However, some *M. panniformis* genomes also aligned with genospecies D (containing *M. aeruginosa* and unidentified *Microcystis* sp.). Genospecies B, H, and I contained both *M. aeruginosa* and *M. wesenbergii.* Genospecies J and M, as well as cluster 20, contained both *M. aeruginosa* and *M. flos-aquae*. Strains identified as *M. viridis* appeared in genospecies G and cluster 21. The nine genomes that did not cluster in either tree included six strains identified as *M. aeruginosa* (the outgroup Ma_SC_T_19800800_S464, Ma_W13_11, Ma_MB_F_20061100_S20D, Ma_QC_C_20070703_M131, Ma_NIES4325, and Ma_KW), one *M. wesenbergii* strain (Mw_MB-S_200031200_S109D), the only high-quality *M. novacekii* strain (Mn_MB_F_20050700_S1D), and one unclassified *Microcystis* strain (Msp_0824).

**Fig. 3.**
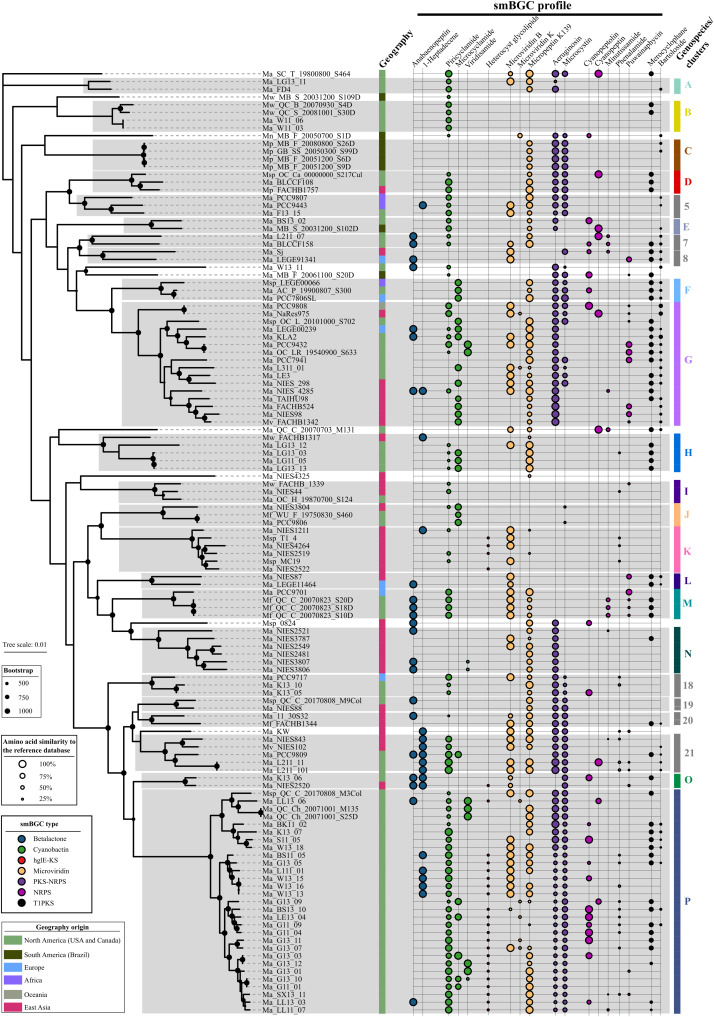
Profile of smBGCs across *Microcystis*. Bootstrap support values were calculated from 1000 replicates; values above 500 are indicated. *M. aeruginosa* Ma_SC_T_19800800_S464 was used as an outgroup. From left to right: 1, geographic origin of isolates; 2, smBGC; 3, *Microcystis* genospecies or groups. Colored bars and letters on the right side of the figure indicate genospecies supported by ANI and dDDH clustering. Gray bars and numbers indicate groups that were not supported by ANI and dDDH clustering. The tree bar scale indicates the number of nucleotide substitutions per site. The area of circles indicates the amino acid similarity to the reference database.

On the basis of the reported geographic origins of each strain, five genospecies revealed some level of geographic fidelity (Fig. 3). All strains within genospecies A, B, and P were isolated from lakes in North America (Canada or United States). However, North American strains are also grouped into genospecies D, E, F, G, H, I, J, M, and O, with 12 genomes falling outside the 16 putative genospecies. Genomes within genospecies K and N were isolated from lakes in East Asia, but East Asian genomes also grouped with genospecies D, G, H, I, J, L, and O, with nine genomes falling outside the 16 putative genospecies. Strains from genospecies C were all isolated from lakes in Brazil, but three Brazilian genomes did not cluster with any of the 16 putative genospecies. Only genospecies F and G appear to be more cosmopolitan, with genospecies F associated with North America, Africa, and Europe and genospecies G associated with North America, East Asia, Europe, and Australia. While there was some evidence for geographic relationships among some of the putative genospecies, ANI, dDDH, and core gene genetic distance revealed little relationship to geographic distance, with geographic distance explaining less than 7% of the variance in ANI, dDDH, or core gene genetic distance values (fig. S3).

Putative secondary metabolite biosynthetic gene clusters (smBGCs) constituted part of the *Microcystis* accessory genome and an average of 6.6% of the total genome across all 122 *Microcystis* strains. Seven general smBGC types were consistently identified by antiSMASH, although their distribution varied across genospecies (Fig. 3). For example, most genospecies and groups, except C, O, 8, and 19, had cytotoxic cyanobactin-type smBGCs (piricyclamide, microcyclamide, and viridisamide A). However, heterocyst glycolipid synthase-like PKS (hglE-KS)–type heterocyst glycolipid synthases were detected only in genospecies K, O, and P. Serine protease–inhibiting microviridins (microviridin B, K, and K139) were detected in all genospecies except B, I, and J. The cytotoxic nonribosomal peptide synthetase/polyketide synthase (NRPS/PKS)–type gene clusters (aeruginosin and microcystin) were found in all genospecies and groups except genospecies B, H, I, K, L, and M and group 7. The first and third most abundant smBGCs across all genomes were terpene-type (*N* = 377) and bacteriocin-type (*N* = 208) gene clusters that did not correspond to any known smBGCs in the antiSMASH database (fig. S4).

### Marker genes

Analysis of genetic markers standard to bacterial taxonomy (16*S* rRNA and its various hypervariable regions, ITS of the rRNA operon, and the cpcBA-IGS) confirmed the low power of these traditional species markers in resolving *Microcystis* taxonomy. Linear correlations between the genome-based parameters (i.e., ANI, dDDH, and core gene similarity) and the 16*S* rRNA sequence similarities were weak (Table 1). Complete 16*S* rRNA genes (including hypervariable regions, e.g., V3 and V4) were detected in only 61 of 122 genomes with sequence similarities ranging from 99.3 to 100% (table S4). Notably, some genomes from different genospecies had identical 16*S* rRNA sequences (c.f., strain Mp_FACHB 1757 in genospecies D, Ma_PCC7806SL in genospecies F, and strain Ma_PCC9808 in genospecies G) (table S4). Like the 16*S* sequences, both the ITS (*N* = 61 complete sequences) and the cpcBA-IGS (*N* = 122 complete sequences) showed weak correlations with ANI, dDDH, and core gene similarity (ITS: *r*^2^ = 0.242, 0.237, and 0.220; cpcBA-IGS: *r*^2^ = 0.429, 0.449, and 0.411) (see fig. S5 for cpcBA-IGS phylogeny).

**Table 1.** Coherence between proposed marker genes and genome-based similarity measures and genospecies and cluster assignment. Squared Pearson correlation coefficients (*r*^2^) between marker gene similarities and ANI, digital dDDH values, and core gene similarity values, as well as cluster fidelity of the marker gene placement relative to the core gene tree (placement/expected), for both the 16 genospecies and 23 clusters. All comparisons are pairwise (16*S*, *N* = 1830; ITS, *N* = 1830; all others, *N* = 7260). Cluster fidelity was not possible for 16*S* rRNA or ITS due to the low number of available sequences (*N* = 61 for both). Bold correlations were significant at *P* ≤ 0.05.

Marker gene	ANI	dDDH	Core gene similarity value	Genospecies assignment fidelity	Cluster assignment fidelity
Traditional					
16*S* rRNA	0.1167	0.1058	0.0999	–	–
ITS	0.2421	0.2373	0.2196	–	–
cpcBA-IGS	0.4289	0.4493	0.4106	90.4	86.7
Proposed					
*acrB*	**0.6846**	**0.7002**	**0.7333**	95.8	92.0
*amtB*	**0.7565**	**0.7442**	**0.7183**	100	95.6
*cpoB*	**0.7110**	**0.7211**	**0.7511**	100	91.2
*dppA*	**0.6502**	**0.6515**	**0.6632**	98.9	92.9
*glnA*	**0.5881**	**0.6091**	**0.6447**	100	97.4
*helY*	**0.7243**	**0.7185**	**0.7566**	97.8	95.6
*murC*	**0.7430**	**0.7191**	**0.6394**	100	93.8
*recJ*	**0.7013**	**0.6953**	**0.7104**	100	89.4
*sbcC*	**0.7641**	**0.7619**	**0.8308**	100	94.7
*trpE*	**0.6226**	**0.6358**	**0.6351**	97.8	92.0
*yccS*	**0.7575**	**0.7495**	**0.7754**	100	95.6

Among the 1452 single-copy core genes, 303 were longer than 1400 bp (i.e., comparable in size to the 16*S* rRNA gene). From these genes, we identified 11 genes that could resolve trees similar to the core gene tree and thus serve as taxonomically diagnostic marker genes (table S5). The ratio of variable sites to conserved sites (*44*) varied from 0.148 to 0.250, indicating that the gene sequences contain both highly conserved regions for primer design and hypervariable regions for phylogenetical analysis. Hypervariable regions were separated by highly conserved regions (fig. S6), allowing us to generate two sets of primers for each gene designed to amplify 1000- to 1500-bp fragments and 300- to 400-bp hypervariable fragments, respectively (table S5). These 11 genes included three transporter genes (*amtB*, *dppA*, and *yccS*), two exonuclease genes (*recJ* and *sbcC*), and two ligase genes (*glnA* and *murC*). All genes reflected overall genome evolution indicated by concordance with ANI, dDDH, and the core gene similarities (Table 1). Correlations between the 11 marker genes and the ANI, dDDH, and core gene similarity ranged from 59 to 83%, while correlations of the 16*S* rRNA gene to the same measures ranged from 9 to 11%. Moreover, the sequence similarity of marker genes ranged from 92.2 to 100%, compared with the similarity of 16*S* rRNA gene sequences that ranged from 99.3 to 100%, indicating that our 11 marker genes have diverged at a higher rate than the 16*S* rRNA gene, thus ultimately allowing more accurate identification of small-scale differences between *Microcystis* genospecies. Phylogenetic trees produced from these genes produced monophyletic groupings similar to those of the core gene– and the pangenome-based phylogenies (i.e., cluster assignment accuracy of >89%). The *sbcC* gene showed the highest concordance with ANI, dDDH, and core gene similarities, although none of the 11 marker genes individually generate 100% cluster fidelity compared with the core gene tree. However, bootstrapped trees inferred from concatenated sequences [see (*45*)] of pairs of the 11 genes (table S6) revealed that the concatenation of four pairs of these marker genes (*acrB* + *glnA*, *amtB* + *sbcC*, *glnA* + *helY*, and *helY* + *sbcC*) could each generate phylogenetic trees with 100% cluster fidelity compared with the core gene phylogeny. The utility of the four gene pairs was confirmed by classifying 106 *Microcystis* MAGs from Lake Champlain and Pampulha Reservoir (fig. S7) (*14*). Even for cases in which none of these gene pairs were available, application of single marker genes to *Microcystis* MAGs used in in the studies of Pérez-Carrascal et al. (*14*) and Cook et al. (*3*) were able to provide first approximations of *Microcystis* identity and diversity (table S7).

## DISCUSSION

Accurate classification of *Microcystis* species is essential for biological inquiry and will likely affect future management of *Microcystis*-based harmful algal blooms (HABs). Unfortunately, traditional morphospecies and DNA sequence approaches have provided contradictory and insufficient detail for advancing the study and understanding of *Microcystis*-based HABs (*9*, *46*). As in past evaluations [e.g., (*4*, *5*, *28*)], our work found that standard bacterial taxonomic markers (complete and hypervariable regions of the 16*S* rRNA gene, ITS, and cpcBA-IGS sequences) were not able to consistently resolve the 23 monophyletic, genomically coherent clusters that were identified through our whole-genome approach. Moreover, quantitative polymerase chain reaction (PCR) approaches for quantification of toxic genotypes using abundances of microcystin (*mcy*) genes fail to inform management of public health risk as *mcy* gene numbers do not correlate with environmental microcystin concentrations (*47*–*49*). Whole-genome–based taxonomy has shown good potential for distinguishing toxic and nontoxic *Microcystis* strains, except for those in genospecies G and J and group 8, in which both ecotypes are included. Although whole-genome based taxonomy is also of little direct value to public health risk management, our classification scheme offers a strong foundation for robust characterization of species-specific ecologies, which can then inform management.

Our analysis of sequence similarities found that at least 16 of the 23 clusters met putative genospecies criteria, with as many as 30 or more total possible genospecies to be delimited as more genomes become available. We posit that ANI and dDDH can be used in combination to classify *Microcystis* genospecies using the following rule: Any *Microcystis* strain exhibiting ANI and dDDH values of ≥0.970 and 0.750, respectively, to a strain from a valid genospecies, belongs to that genospecies, as long as the strain also exhibits ANI and dDDH values of <0.970 and 0.750 compared with other genospecies. For example, when a new *Microcystis* genome M054S2 (*14*) was placed into the core gene tree it joined existing genospecies G, which was supported with these criteria with the lowest within group ANI and dDDH of 0.985 and 0.867, respectively, and the maximum between group ANI and dDDH of 0.964 and 0.699. These threshold values of ANI and dDDH will allow genospecies classification of newly sequenced strains as the reconstruction of a core genome phylogenetic tree of all representatives is not practical each time a new genome becomes available. It is worth noting that our proposed ANI and dDDH thresholds may need to be reevaluated as more genomes are analyzed, especially with the addition of more closed (i.e., complete) genomes that will improve the identification of complete orthologous regions, as well as pan- and core genes. Ultimately, more complete genomes will increase reliability of ANI and dDDH thresholds across the phylogeny.

Pangenome analysis also revealed that less than half (35.6%) of each genome consisted of shared core genes, with 26% of pan genes being unique to a single strain. These results corroborate a highly plastic accessory *Microcystis* genome (*13*, *38*). By using both core sequence similarities and pan-gene presence-absence to construct phylogenetic trees, our proposed *Microcystis* classification considers the phylogenetic relationships among core genes that reflect evolution during adaptation and speciation, as well as phylogenetic relationships among noncore genes that reflect ecologically relevant species-environment interactions (*13*). Moreover, our proposed classification scheme supports Rosselló-Móra and Amann’s (*21*) definition of a bacterial species, which is considered to represent “a category that circumscribes monophyletic and genomically and phenotypically coherent populations of individuals that can be clearly discriminated from other such entities by means of standardized parameters.” A challenge ahead will be the phenotypic characterization of our proposed putative genospecies.

Although there is considerable debate regarding the roles of dispersal and environmental conditions in microbial biogeography (*5*, *50*, *51*), some strains within a genospecies seemed to reflect specific biogeography, with genospecies C all from South America, genospecies N from Southeast Asia, and genospecies P from North America. However, overall, we did not detect general geographic patterns in ANI, dDDH, or core gene phylogenetic distance metrics. Thus, it appears that geographic dispersal is not a major driver of *Microcystis* phylogenetic relationships. By contrast, our analysis of secondary metabolites corroborates the possibility for environmental filtering and ecologically relevant genomic variation between strains (*3*, *13*, *52*). While the specific functions of many secondary metabolites in bacteria are unknown, in general, they mediate response of species to other organisms or the environment. For example, secondary metabolites can serve as weapons against competitors or predators and regulators of symbiosis and nutrient acquisition and transport (*53*). Hence, secondary metabolites may provide some insight into certain ecotypes [sensu (*54*)] that may have arisen through environmental adaptation. For example, microcystin is thought to bind to enzymes as a means of protecting them from degradation by reactive oxygen species (ROS) produced during a bloom (*55*), whereas nonmicrocystin producing strains use enzymatic degradation of ROS (*4*). Hence, observed succession from toxic to nontoxic strains during the course of a bloom (*56*) may reflect a shift in ROS protection strategies from one based on production of high-nitrogen–containing microcystin to one based on enzymatic degradation of ROS when nitrogen becomes limiting (*57*, *58*). Our classification scheme could be used to differentiate between strains with the potential to produce microcystin (genospecies C, D, F, O, and P and groups 5, 18, 19, 20, and 21) from those without microcystin capabilities (genospecies A, B, E, H, I, K, L, M, and N and group 7), except for genospecies G and J and group 8, in which both putatively toxic and nontoxigenic strains are included. Whether all different smBGC profiles correspond to different *Microcystis* ecotypes remains unknown. However, recent analysis suggests that smBGC profiles may not correlate strongly with core genome phylogeny due to the high prevalence of HGT events (*59*). Cao *et al.* (*59*) recently determined that four transferred genes that could encode for microcystin biosynthesis in *M. panniformis* FACHB-1757 (member of genospecies D in our classification) were likely derived from *Planktothrix* via HGT. Given the high nitrogen content of microcystins and their potential role in protection from ROS, a selective advantage is likely afforded to microcystin-capable *Microcystis* genotypes in nitrogen-rich systems.

While additional *Microcystis* genomes will strengthen the genomic classification of *Microcystis*, we are now better positioned to begin reassessing ecotypes and ecological niches of *Microcystis* [e.g., (*4*)] within the context of a proposed phylogeny. To aid in advancing the phenotypic characterization of *Microcystis* globally without the need for whole-genome sequencing and analysis, we propose 11 new marker genes that in select concatenated pairs, or even individually for first approximations, can replace the traditional marker gene approaches for identifying *Microcystis*. In particular, we suggest the pairing of *glnA* with either *acrB* or *helY* or *sbcC* with *helY* or *amtB* for identifying *Microcystis* genospecies. These proposed marker genes may be used to reduce the need for shotgun sequencing and analysis efforts to identify genospecies by enabling researchers to PCR amplify the single gene(s) and/or hypervariable regions with the primers proposed in table S5. Once amplified, either long- or short-read sequencing may be performed for full-length amplicons or hypervariable specific regions, respectively, and the resulting sequence compared to the existing sequences to identify the most closely related taxon and ultimately the genospecies classification. Once identified, characterization of nutrient uptake kinetics, light- and temperature-dependent growth rates, microbiomes, and other phenotypic traits [sensu (*3*, *60*, *61*–*63*)] can be defined and assigned for each genospecies, with the end result being a more concrete understanding of *Microcystis* diversity, ecology, and evolution.

Uncertainty in available metadata may limit the conclusions of our study. Namely, we cannot confirm that the provided morphospecies classifications assigned to these genomes in NCBI have been verified microscopically or using consistent criteria. These morphospecies identities may have been assigned via sequence similarity to previously deposited genomes with inaccurate classifications, thus propagating existing errors. In addition, identification may have been assigned with inappropriate methods (e.g., marker genes and universal OGRI). Hence, we must rely on comparing our genomic classification to the morphospecies name as assigned in NCBI in our analysis, although it may not be entirely accurate. We selected genomes from NCBI with the genus labeled as “*Microcystis*” (see Materials and Methods for further details). We did not find evidence that any *Microcystis* genome was misclassified at the genus level. Specifically, our use of closely related outgroups, such as *Aphanocapsa montana* BDHKU210001 (see fig. S1) to identify the root *Microcystis* genome indicated that all *Microcystis* genomes were more closely related to each other than to any closely related outgroup.

We offer a new whole-genome–based taxonomy, along with genetic markers that provide the beginnings of a new *Microcystis* classification system that will advance research characterizing the *Microcystis* niche. Our proposed *Microcystis* classification considers phylogenetic relationships among core genes (reflecting evolution during speciation), as well as phylogenetic relationships among noncore genes (reflecting species-environment interactions). This whole-genome–based classification approach overcomes historical limitations in *Microcystis* taxonomy, long troubled by morphological plasticity and cryptic species and ecotypes. This innovation will advance ecological and evolutionary research and management of this important HAB taxon.

## MATERIALS AND METHODS

### Study design

The current taxonomy of *Microcystis* is based on morphology. The problematic morphological taxonomy of *Microcystis* was initially challenged by the introduction of 16*S* rRNA genes. However, 16*S* rRNA genes are still insufficient for taxonomic resolution at the species level. At present, an increasing number of complete or draft genome sequences of *Microcysts* have been published, allowing for the application of whole-genome–based taxonomic tools including OGRI. However, as the OGRI threshold may not be applicable to *Microcystis* species, we needed to reevaluate the OGRI threshold. In this study, we first downloaded all *Microcystis* genomes from the NCBI database and then removed low-quality or duplicated genomes. We then identified monophyletic and genomically coherent clusters supported by both core gene– and pan-gene–based phylogenies. The clusters that were also supported jointly by ANI and dDDH clustering would be considered genospecies. We proposed new candidate marker genes that, when phylogenetically compared, produced similar groupings as the core gene– and pangenome-based phylogenies.

### Dataset preparation

We downloaded all 173 complete and draft *Microcystis* genomes from the NCBI database (https://ncbi.nlm.nih.gov/genome/), on 8 August 2021. These genomes consisted of 6 complete and 114 draft genomes identified as *M. aeruginosa*, 11 draft genomes identified as *M. flos-aquae*, 10 draft genomes identified as *M. wesenbergii*, 1 complete and 8 draft genomes identified as *M. panniformis*, 1 complete and 5 draft genomes identified as *M. viridis*, 2 draft genomes identified as *Microcystis novacekii*, 1 draft genome identified as *M. elabens*, and 1 complete and 13 draft genomes with uncertain taxonomic status. We narrowed this group by selecting the highest-quality genome for strains that have been sequenced multiple times (*N* = 2 or 3) using “dereplicate” function of dRep v3.0.0 (*64*) with the default settings, leaving a total of 9 complete and 136 draft *Microcystis* genomes. We further filtered this dataset by removing poorly assembled genomes based on number of contigs (>1000), completeness (<97%), and contamination (>1%) using CheckM (version 1.1.3) using cyanobacteria-specific marker genes and default parameters (*65*). This quality filtering left 122 genomes that constitute the basis for further analysis (table S1). The estimated genome size was adjusted to account for its estimated completeness and contamination using the equation: estimated genome size = assembly genome size/estimated completeness/(1 + estimated contamination) (*66*).

### Pangenome analysis

Identification of the pangenome requires first creating a rooted tree to determine taxonomic relationships and species evolution (*67*). However, introducing genomes of other genera for pangenome analysis would notably reduce the core gene number. To minimize this issue, we took a two-step approach to create the phylogenetic trees. Phylogenetic analyses were first performed with 122 *Microcystis* genomes along with one of several related species (based on ANI values), including *A. montana* BDHKU210001 (accession number: NZ_JTJD00000000.1), *Cyanobium gracile* PCC-6307 (NC_019675.1), *Gloeocapsa* sp. PCC-7428 (GCA_000317555.1), and *Synechococcus elongatus* PCC-6301 (AP008231.1) (*5*). Trees were constructed using the up-to-date bacterial core gene pipeline, which infers a maximum likelihood phylogeny using the concatenated sequences of 92 universal single-copy core genes (*68*) identified across all *Microcystis* and each related, non-*Microcystis* species. All phylogenetic trees showed that *M. aeruginosa* Ma_SC_T_19800800_S464 was the basal taxon (fig. S1). We then eliminated all non-*Microcystis* strains and used *M. aeruginosa* Ma_SC_T_19800800_S464 as the outgroup in all subsequent phylogenetic analyses.

The entire gene pool of a bacterial clade, referred to as a pangenome, can be divided into a core genome (a set of genes shared by all genomes), accessory genes (a set of genes present in some genomes), and unique genes (a set of genes found in only one genome) (*69*). Genomes were annotated using Prokka (version 1.14.5, with default parameters) (*70*), followed by processing with PEPPAN (version 1.0.5), which provides consistent gene and pseudogene annotation and identification and exclusion of paralogs (*71*). A gene-presence tree was constructed using PEPPAN_parser. Single-copy core genes were aligned using MAFFT (version 7.471) (*72*) and concatenated using the FASconCAT-G software (version 1.04) (*73*). The maximum likelihood tree was inferred using RAxML-NG (version 1.0.3) (*74*) using the GTR + G +I model, which was selected as the best model by ModelTest-NG (version 0.1.7) and 1000 bootstrap replications (*75*). Trees were visualized using iTOL (http://itol.embl.de/) (*76*). Putative smBGCs encoded within the *Microcystis* genomes were identified and analyzed using the online server antiSMASH 6.0 (*77*) with detection strictness set to “relaxed” and all extra features activated.

### Genospecies boundaries

We defined boundaries between *Microcystis* genospecies based on Chun *et al.*’s (*37*) OGRI, which relies on multiple sequence similarity approaches. For each pair of 122 genomes, we calculated ANI using BLASTN (*78*) and digital dDDH (*78*). The dDDH values between genome pairs were predicted using the GGDC 2.1 web server (*79*) available at http://ggdc.dsmz.de/distcalc2.php with the recommended Formula 2 and the alignment tool BLAST+. Heatmap and hierarchical cluster analyses were performed using the “pheatmap” package with default parameters in R (*80*). Average nucleotide identities were high among the 122 genomes. In pairwise genome comparisons, 70% of ANI values (range = 94.0 to 99.9%; median = 95.3%) were above the nominal prokaryote species threshold of 95%, indicating that existing ANI criteria may not be useful in delimiting *Microcystis.* In contrast, only 11% of dDDH values (range = 56.0 to 99.8%; median = 63.2%) were above the nominal prokaryote species threshold of 70%, suggesting that dDDH may be more useful. Using these OGRI results, we examined all pairwise ANI and dDDH values of the suspected monophyletic clusters from the core gene phylogeny. We evaluated all within-cluster pairwise ANI and dDDH values to find the lowest within-cluster value for each metric and then identified the highest pairwise ANI and dDDH values for any member of a cluster to all other genomes outside of that cluster (i.e., “between-cluster” comparisons). We identified new thresholds by identifying all clusters with at least three genomes in which the lowest within-cluster ANI and dDDH values were higher than the highest between-cluster ANI and dDDH values. We then found the lowest ANI and dDDH values from this subset of clusters meeting these criteria and set that as the new threshold for *Microcystis* genospecies. Clusters of two or more *Microcystis* genomes were defined as putative genospecies if they were (i) monophyletic and genomically coherent as identified by core and pan-gene trees and (ii) met our within-cluster and between-cluster OGRI thresholds (*37*).

### Identification of marker genes for assigning *Microcystis* taxonomy

Given that use of the 16*S* rRNA genes for species-level resolution within *Microcystis* is limited [e.g., (*5*)], we explored the possibility of using single-copy core genes as taxonomic markers for *Microcystis* genospecies [sensu (*41*, *42*)]. Potential marker genes initially were identified as single-copy core genes with a length of >1400 bp (comparable to the size of the 16*S* rRNA gene). These were aligned within the 122 sequences for a single gene and used to build trees based on the maximum likelihood approach implemented in RAxML-NG 1.0.3 (*74*). The topological structures of the marker gene phylogenetic trees were compared with the phylogenetic tree generated from the core genome and genes with the best genospecies placement when compared to the core gene tree were considered marker genes. In addition, pairwise nucleotide sequence similarity values obtained for the marker genes were calculated and compared to dDDH, ANI, and pairwise core gene nucleotide sequence similarity values by correlation analysis. Pearson’s correlation coefficient was used for the correlation analysis with linear regression.

### Statistical analyses

All statistical analyses were completed in the R statistical environment v.4.0.3 (www.r-project.org). We first tested for associations between OGRIs and single-copy core gene phylogenetical distance. Pairwise genetic distance of concatenated core genes or marker genes between genomes was calculated in MEGA X using the Kimura 2-parameter model (*44*). The calculation of similarity values was performed as follows: similarity percentage = (1 − genetic distance) × 100%. Geographic distance between sites was calculated using the “rdist.earth” function in the R “fields” library v 13.3 using estimated GPS coordinates based on source lake name. Generalized linear models (GLMs) were used to assess the relationships between index measures (using default family = Gaussian, link = identity). Deviation explained by GLMs coupled with *P* values was used to assess the significance and strength of the relationships.

We then evaluated the pairwise correlation (Pearson’s correlation) between dDDH, ANI, and pairwise core gene similarity values with the pairwise nucleotide sequence similarity values obtained for the 16*S* rRNA and other marker genes, individually. Values were considered statistically significant at *P* < 0.05.

## Supplementary Materials

### This PDF file includes:

Figs. S1 to S7 Tables S1 to S7 References

## Notes

View/request a protocol for this paper from *Bio-protocol*.
