# Supplementary material for: Microcystis pangenome reveals cryptic diversity within and across morphospecies
Source: Sci Adv. 2023 Jan 13;9(2):eadd3783. doi: 10.1126/sciadv.add3783 (PMC9839332; doi:10.1126/sciadv.add3783)
Supplement: Supplementary file 1 — Figs. S1 to S7 Tables S1 to S7 References [file sciadv.add3783_sm.pdf]

Science Advances
AAAS

Supplementary Materials for

Microcystis pangenome reveals cryptic diversity within and across morphospecies

Haiyuan Cai *et al.*

Corresponding author: K. David Hambright, dhambright@ou.edu

Sci. Adv. **9**, eadd3783 (2023)
DOI: 10.1126/sciadv.add3783

This PDF file includes:

Figs. S1 to S7
Tables S1 to S7

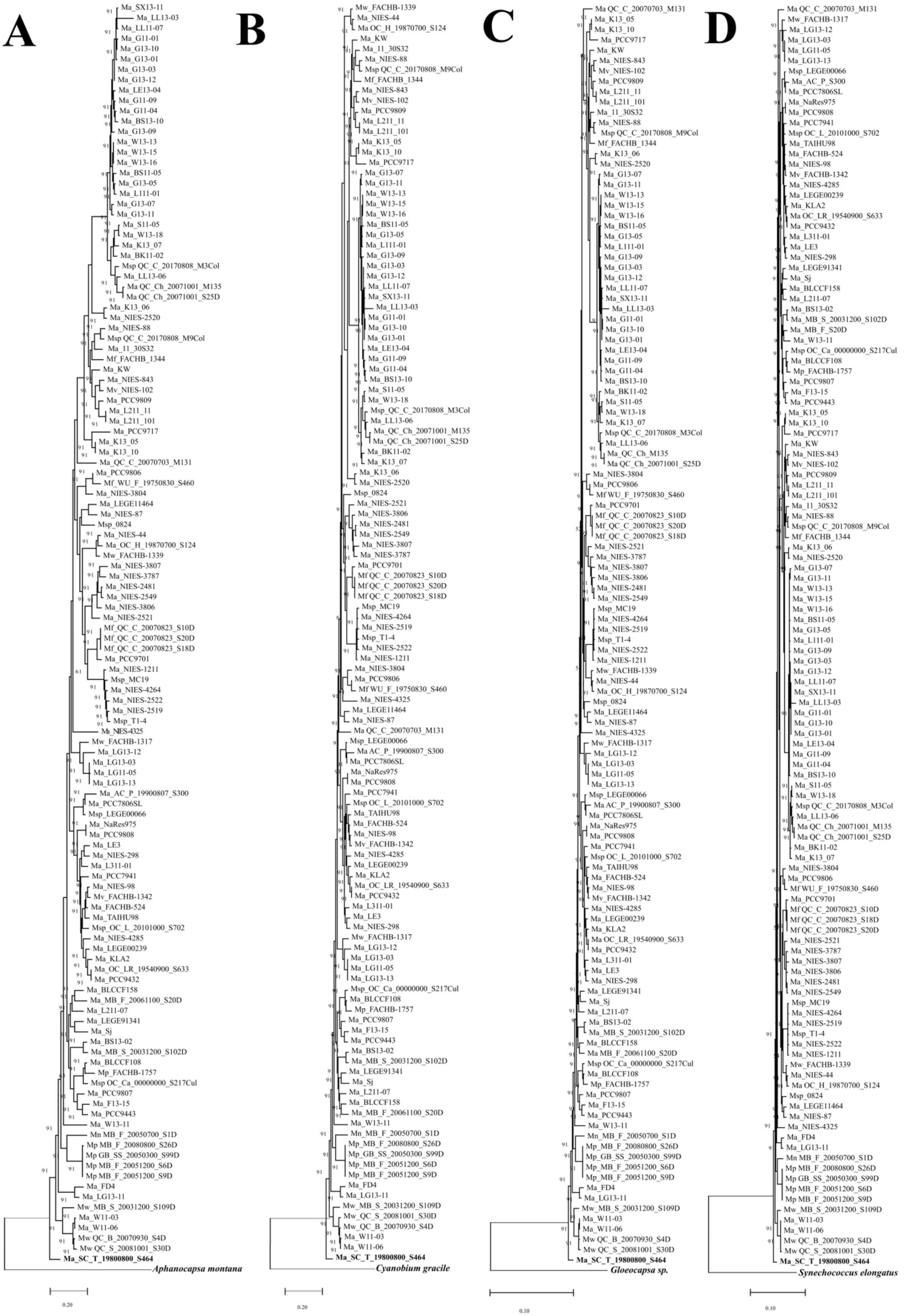
A
B
C
D
Ma_SC_T_19800800_S464
Aphanocapsa montana
Cyanobium gracile
Gloeocapsa sp.
Synechococcus elongatus
0.20
0.20
0.10
0.10

Fig. S1.

Phylogenomic trees of *Microcystis* and close relatives using the up-to-date bacterial core gene (UBCG) set. Phylogenetic analyses were performed with 122 *Microcystis* genomes and closely related species to *M. aeruginosa* NIES-843 (based on ANI values) [Harke, 2016 #109], including *Aphanocapsa montana* BDHKU210001 (accession number: NZ_JTJD00000000.1) (A), *Cyanobium gracile* PCC-6307 (NC_019675.1) (B), *Gloeocapsa* sp. PCC-7428 (GCA_000317555.1) (C), and *Synechococcus elongatus* PCC-6301 (AP008231.1) (D). All phylogenetic trees showed *M. aeruginosa* Ma_SC_T_19800800_S464 (SFBL01) was the root within *Microcystis* clades.

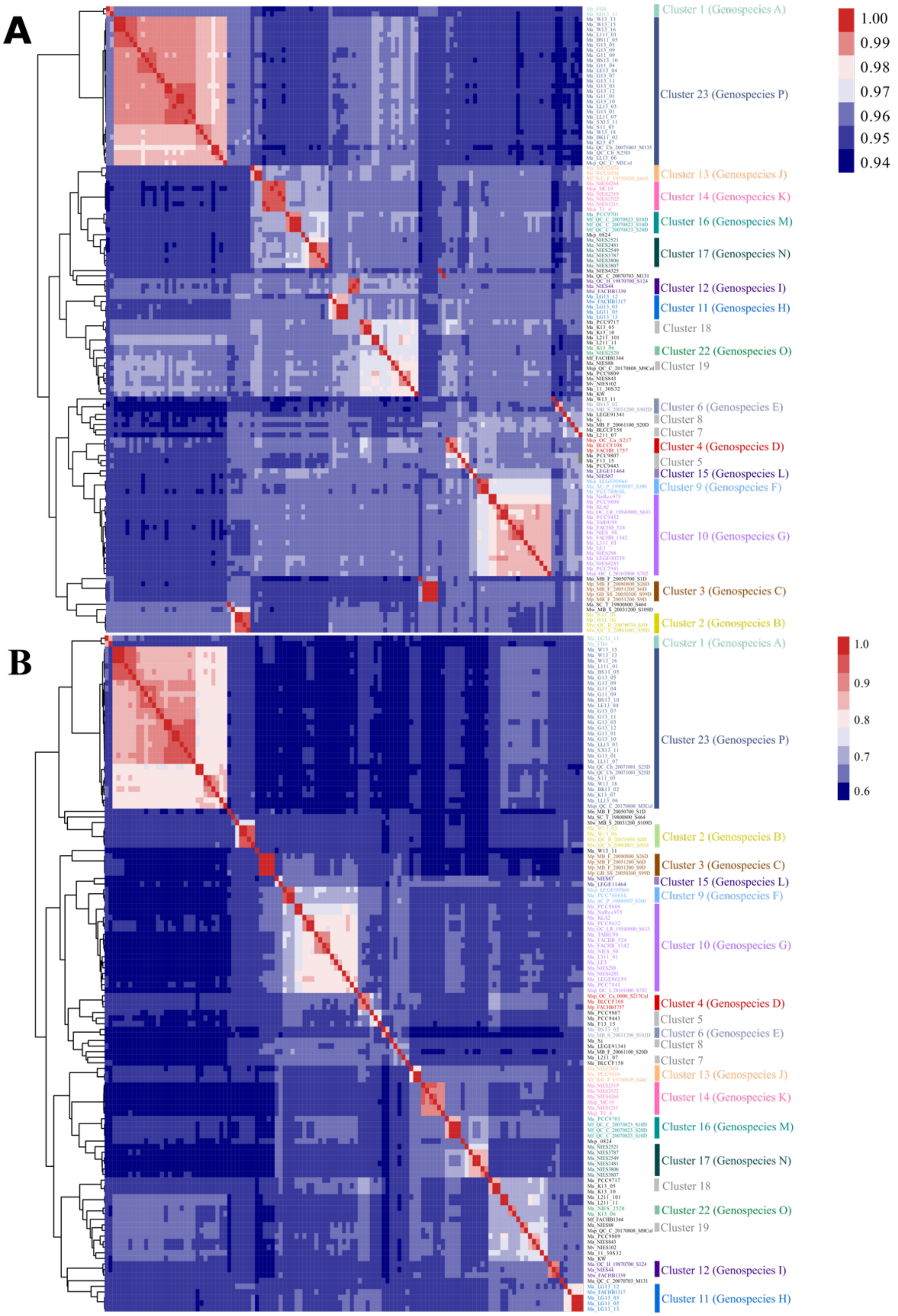

Fig. S2.

Heat map of pairwise ANI (A) and dDDH (B) values for 122 genomes of *Microcystis* strains. Dendrogram shows the hierarchical clustering. Genome ID is listed in Table S1. Heatmap was visualized with pheatmap v1.0.12 with default parameters. Genomes within clusters 20 or 21 do not form ANI or dDDH clusters.

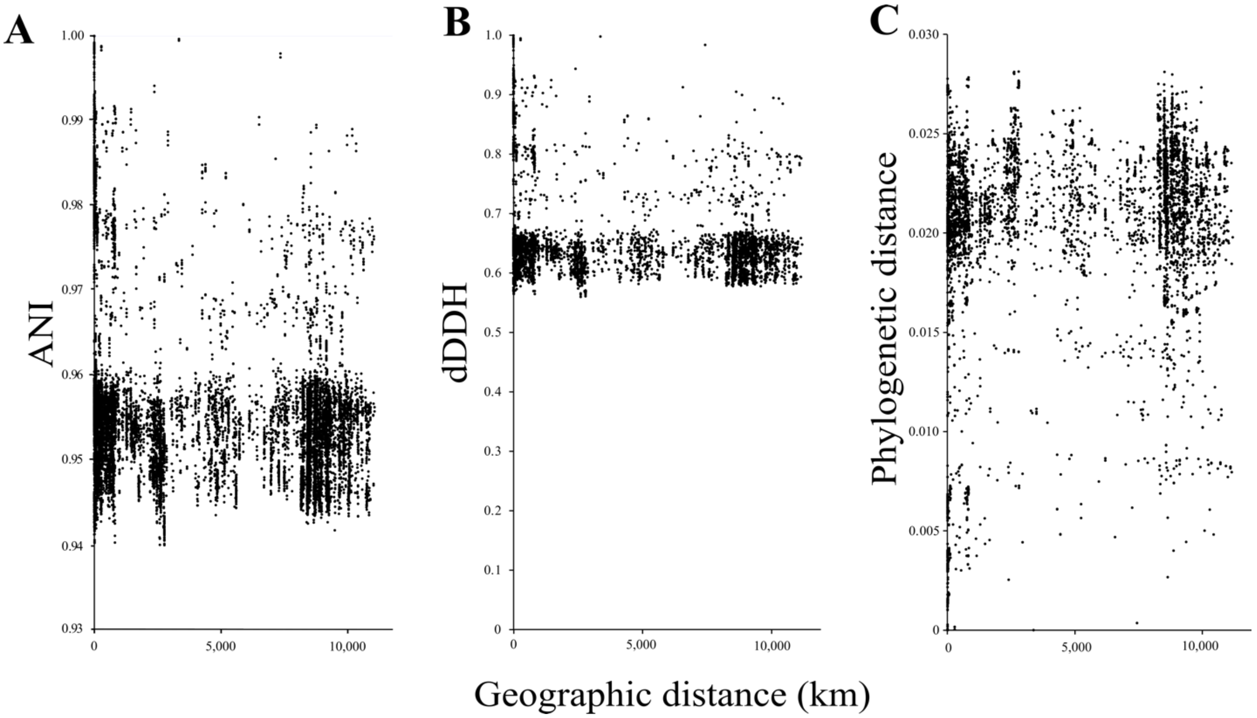

Fig. S3.

Scatter plots of pairwise genome sequence differences and geographic distances between *Microcystis* strains. Geographic distance between sites was calculated using the "rdist.earth" function in the R "fields" library. Generalized linear models (GLM) were used to assess the relationships between pairwise genome sequence differences and geographic distances, with 6.9% of the variance in ANI (A), 5.5% of the variance in dDDH (B), and 6.5% of the variance in phylogenetic distance of core genes (C) explained by geographic distance.

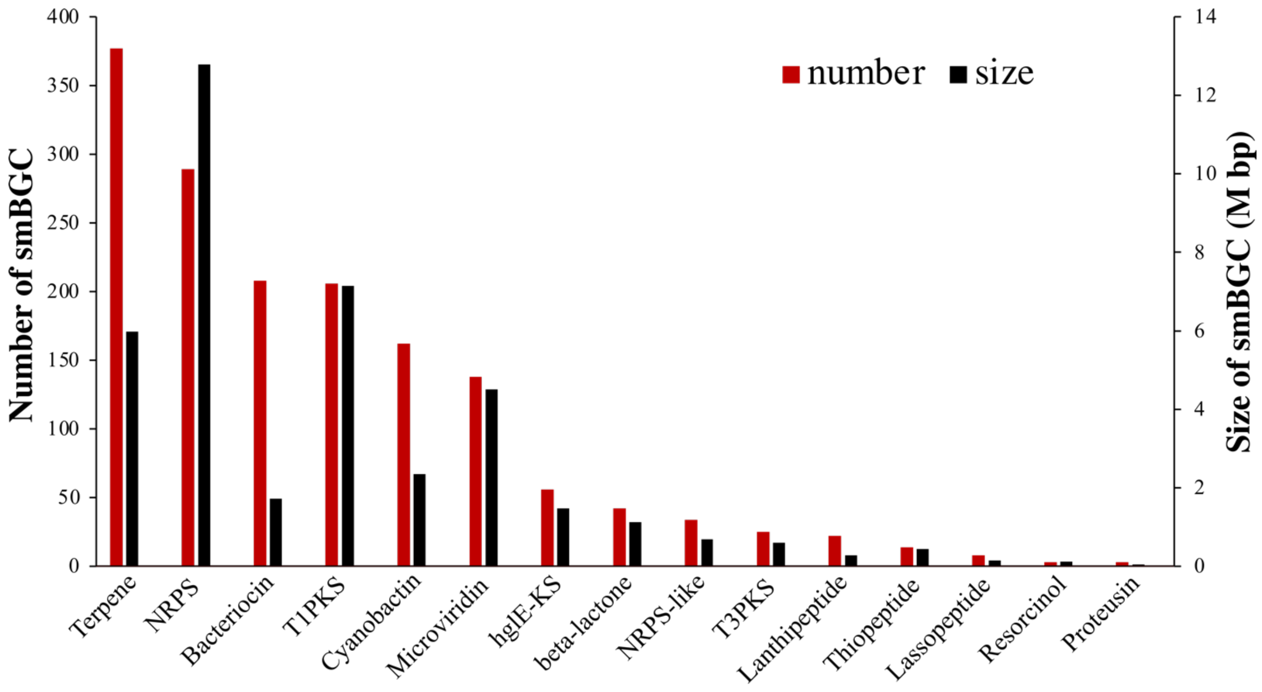

Fig. S4.

Number and size of secondary metabolite biosynthetic gene clusters. The total number and size of secondary metabolite biosynthetic gene clusters (smBGCs) across 122 *Microcystis* genomes.

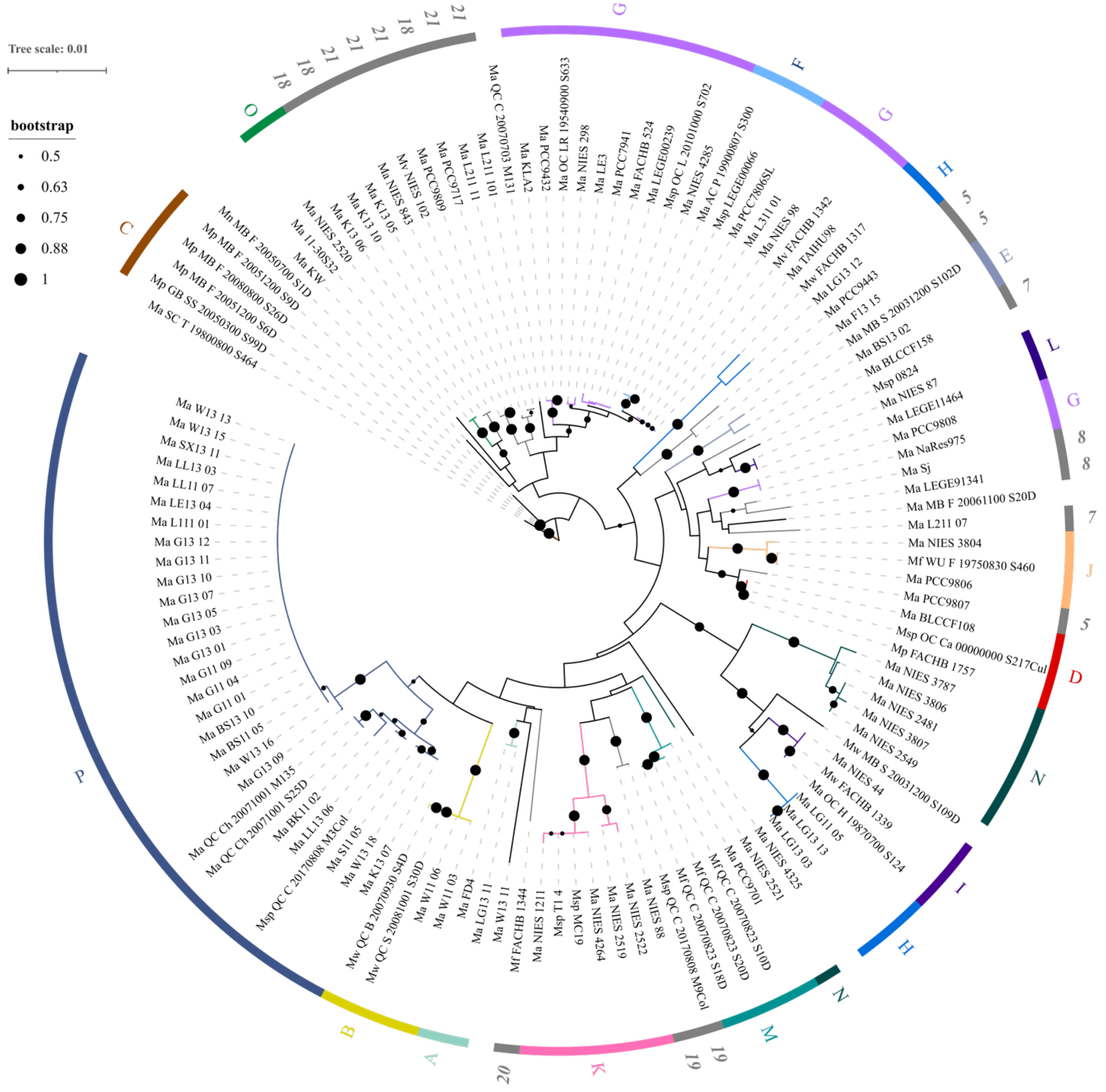

Fig S5.

Maximum Likelihood phylogenetic tree of intergenic spacer of phycocyanin biosynthesis genes (cpcBA-IGS) of 122 *Microcystis* genomes. Bootstrap support values were calculated from 1000 replicates; values above 500 are indicated. Ma_SC_T_19800800_S464 was used as the outgroup. The color segments indicate genospecies supported by ANI and dDDH clustering, and grey segments indicate clusters that were not supported by ANI and dDDH clustering.

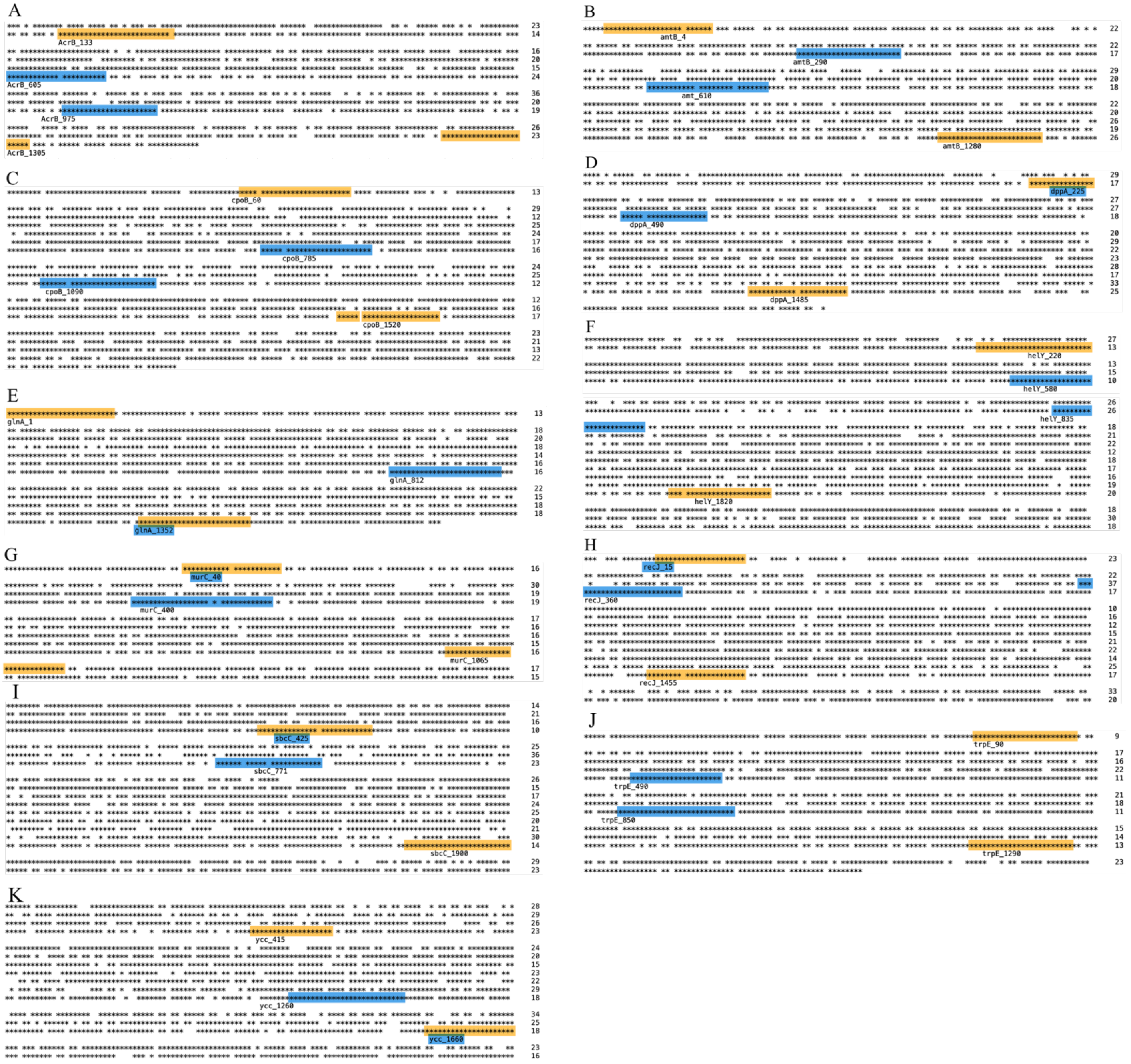

Fig S6.

The distribution of variable and conserved sites of marker genes. Alignments were constructed using *acrB* (A), *amtB* (B), *cpoB* (C), *dppA* (D), *glnA* (E), *helY* (F), *murC* (G), *recJ* (H), *sbcC* (I), *trpE* (J), *yccS* (K) from our 122 genomes. Asterisks (*) indicate conserved sites, gaps ( ) indicate variable sites, and the number to the right of each line indicates the number of variable sites (120 total sites each line). The locations of primers for Sanger sequencing and Illumina sequencing are highlighted in yellow and blue, respectively.

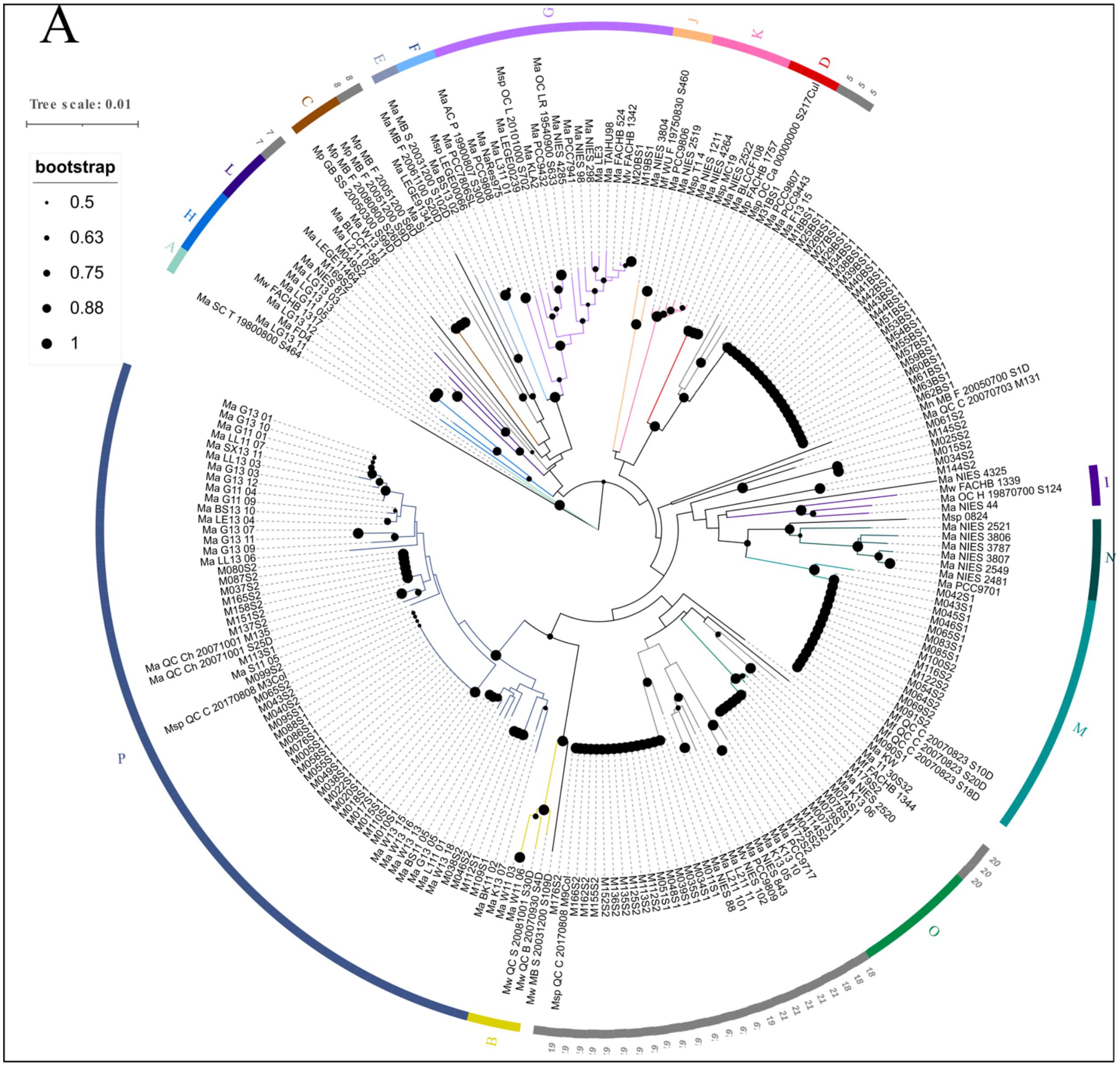
A
Tree scale: 0.01
bootstrap
0.5
0.63
0.75
0.88
1
A
H
L
C
E
F
G
J
K
D
I
N
M
O
B
P

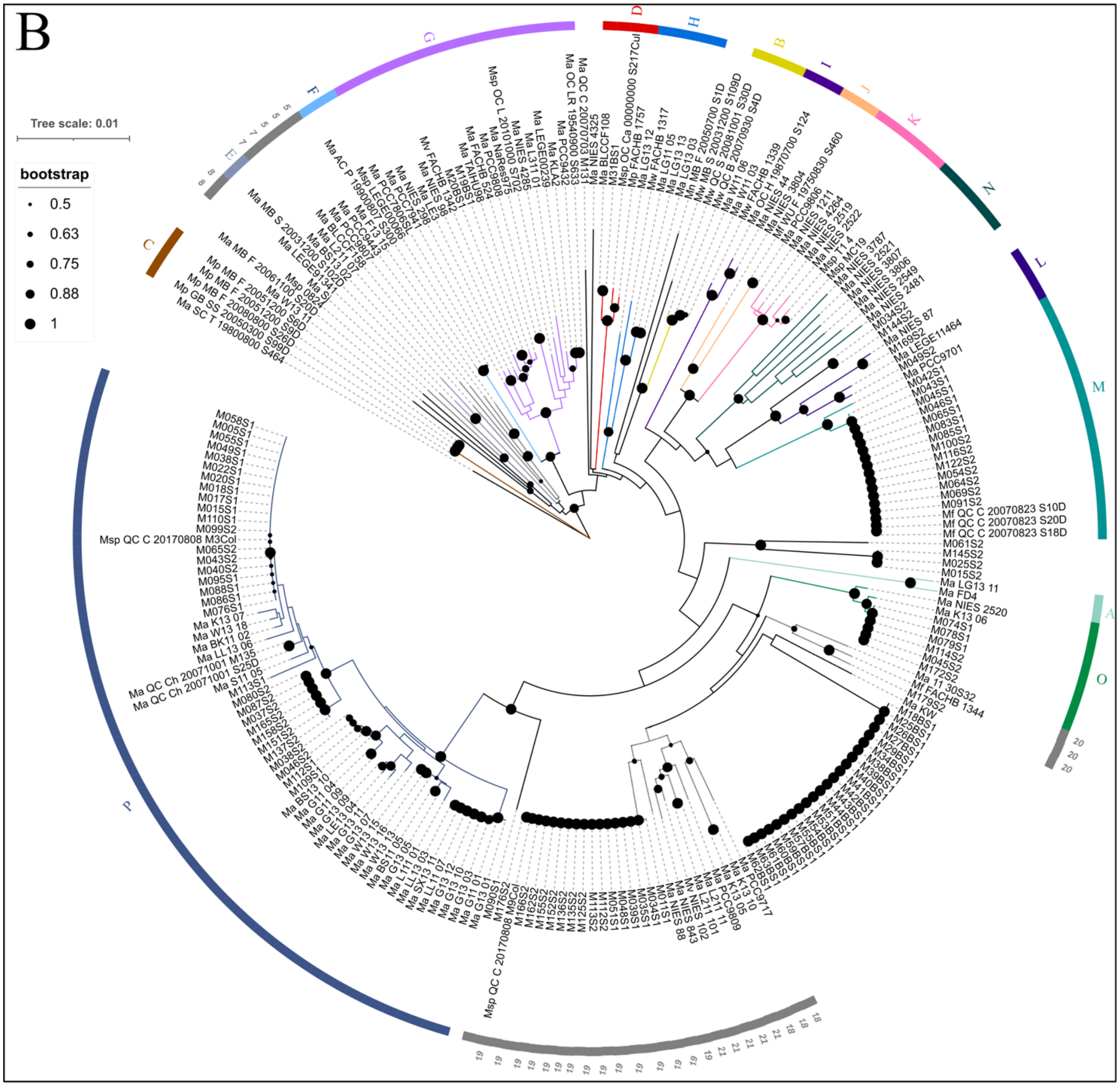

B
Tree scale: 0.01
bootstrap
0.5
0.63
0.75
0.88
1

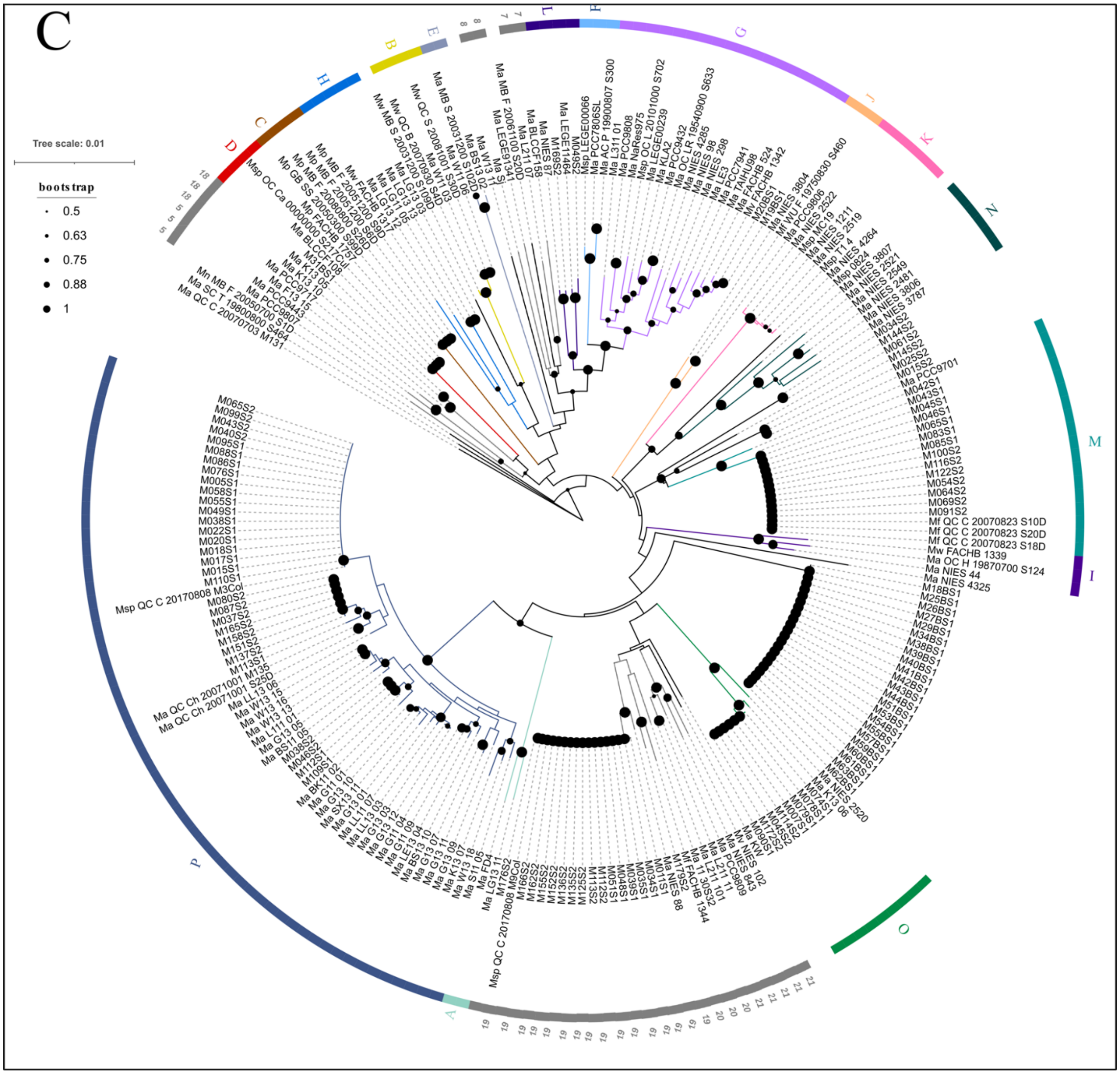
C
Tree scale: 0.01
bootstrap
0.5
0.63
0.75
0.88
1
A
B
C
D
E
F
G
H
I
J
K
L
M
N
O
P

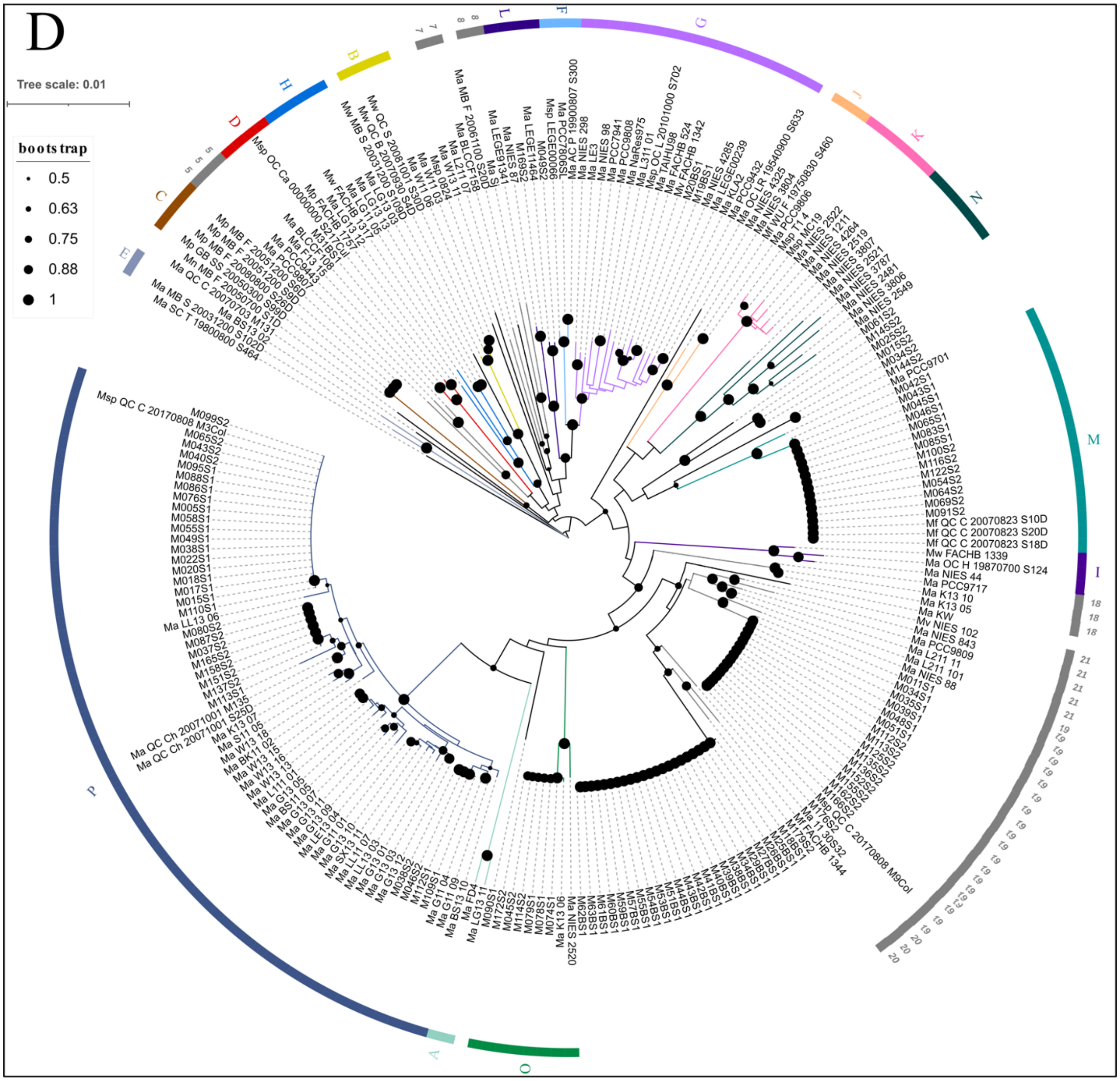

Fig. S7.

Phylogenetic positions of 106 *Microcystis* MAGs from Lake Champlain and Pampulha Reservoir by the concatenation of four pairs of marker genes. Phylogenies were constructed using the concatenation of (A) *acrB*+*glnA*, (B) *amtB*+*sbcC*, (C) *glnA*+*helY*, and (D) *helY*+*sbcC* from our 122 references and extracted from 106 *Microcystis* MAGs from Lake Champlain and Pampulha Reservoir *(14)*. The color segments indicate genospecies supported by ANI and dDDH clustering, and grey segments indicate clusters that were not supported by ANI and dDDH clustering. Ma_SC_T_19800800_S464 was used as the outgroup.

Table S1.
List of the genome sequences used in this study. Abbreviation: Ma=*Microcystis aeruginosa*, Mf= *M. flos-aquae*, Mw=*M. wesenbergii*, Mp=*M. panniformis*, Mv=*M. viridis* , Mn=*M. novacekii*. The nine complete genomes are highlighted in **bold** print.

Strain	Status	Genome size (Mb)	GC%	Number of contigs	Number of protein-coding sequences§	Accession no.
Ma_11_30S32	Draft	5.357	42.7	814	4930	BHVU01
Ma_AC_P_19900807_S300	Draft	4.512	42.5	235	4174	SFBP01
Ma_BK11_02	Draft	4.924	42.6	687	4619	JAADAC01
Ma_BLCCF108	Draft	5.038	42.6	487	4711	JACEGB01
Ma_BLCCF158	Draft	5.168	42.8	512	4802	JACEGC01
Ma_BS11_05	Draft	4.925	42.5	586	4632	JAADAD01
Ma_BS13_02	Draft	4.624	42.3	250	4333	JAADAE01
Ma_BS13_10	Draft	5.180	42.4	549	4902	JAADAF01
Ma_F13_15	Draft	5.435	42.7	446	5064	JAADAG01
Ma_FACHB_524	Draft	4.846	42.6	119	4625	RJVW01
**Ma_FD4**	**Complete**	**5.493**	**42.6**	**2**	**5421**	**NZ_CP046973.1**
Ma_G11_01	Draft	5.240	42.5	735	4923	JAADAH01
Ma_G11_04	Draft	5.001	42.5	634	4693	JAADAI01
Ma_G11_09	Draft	5.068	42.4	668	4831	JAADAK01
Ma_G13_01	Draft	5.072	42.5	765	4722	JAADAL01
Ma_G13_03	Draft	5.125	42.5	802	4884	JAADAM01
Ma_G13_05	Draft	5.110	42.5	644	4861	JAADAN01
Ma_G13_07	Draft	5.086	42.5	684	4848	JAADAO01
Ma_G13_09	Draft	4.912	42.4	638	4638	JAADAP01
Ma_G13_10	Draft	5.116	42.5	689	4829	JAADAQ01
Ma_G13_11	Draft	5.018	42.5	674	4712	JAADAR01
Ma_G13_12	Draft	5.075	42.6	774	4702	JAADAS01
Ma_K13_05	Draft	5.274	42.8	456	4866	JAADAT01
Ma_K13_06	Draft	4.988	42.9	353	4679	JAADAU01
Ma_K13_07	Draft	4.982	42.5	615	4618	JAADAV01
Ma_K13_10	Draft	5.313	42.9	481	4940	JAADAW01
Ma_KLA2	Draft	5.213	42.8	251	4880	VTRR01
Ma_KW	Draft	5.889	42.8	6	5559	MVGR01
Ma_L111_01	Draft	5.012	42.6	596	4723	JAADAX01
Ma_L211_07	Draft	4.934	42.7	268	4557	JAADAY01
Ma_L211_101	Draft	5.105	42.7	394	4829	JAADAZ01
Ma_L211_11	Draft	5.115	42.7	392	4845	JAADBA01
Ma_L311_01	Draft	4.769	42.7	275	4520	JAADBB01
Ma_LE13_04	Draft	5.038	42.5	631	4751	JAADBC01
Ma_LE3	Draft	4.557	42.8	163	4263	MTBS01
Ma_LEGE00239	Draft	5.072	42.5	359	4836	JADEVY01
Ma_LEGE11464	Draft	4.768	42.9	500	4381	JADEXM01
Ma_LEGE91341	Draft	4.485	42.9	280	4059	JADEXY01
Ma_LG11_05	Draft	5.060	42.7	326	4834	JAADBD01
Ma_LG13_03	Draft	5.041	42.7	337	4803	JAADBE01
Ma_LG13_11	Draft	5.080	42.7	426	4882	JAADBF01
Ma_LG13_12	Draft	4.984	42.6	396	4835	JAADBG01
Ma_LG13_13	Draft	5.049	42.7	363	4827	JAADBH01
Ma_LL11_07	Draft	5.151	42.4	779	4844	JAADBI01
Ma_LL13_03	Draft	5.133	42.5	801	4874	JAADBJ01
Ma_LL13_06	Draft	4.822	42.4	594	4561	JAADBK01
Ma_MB_F_20061100_S20D	Draft	4.505	42.7	169	4201	SFBH01
Ma_MB_S_20031200_S102D	Draft	4.636	42.5	246	4291	SFBJ01
Ma_NaRes975	Draft	5.118	42.4	413	4897	MOLN01
Ma_NIES_1211	Draft	4.738	42.8	264	4465	BEIV01
**Ma_NIES_2481**	**Complete**	**4.441**	**42.8**	**2**	**4155**	**NZ_CP012375.1**
Ma_NIES_2519	Draft	4.937	42.7	412	4703	BHVO01
Ma_NIES_2520	Draft	5.272	42.8	475	4837	BHVP01
Ma_NIES_2521	Draft	4.275	43.1	176	4027	BHVQ01
Ma_NIES_2522	Draft	4.895	42.7	412	4627	BHVR01
**Ma_NIES_2549**	**Complete**	**4.301**	**42.9**	**2**	**3985**	**NZ_CP011304.1**
**Ma_NIES_298**	**Complete**	**5.015**	**42.6**	**2**	**4742**	**NZ_CP046058.1**

Ma_NIES_3787	Draft	4.378	43.0	214	4125	BJCH01
Ma_NIES_3804	Draft	4.493	43.0	238	4225	BJCI01
Ma_NIES_3806	Draft	4.523	43.0	235	4179	BJCJ01
Ma_NIES_3807	Draft	4.370	43.0	228	4065	BJCK01
Ma_NIES_4264	Draft	4.970	42.7	468	4671	BHVS01
Ma_NIES_4285	Draft	5.255	42.6	444	4928	BIFY01
Ma_NIES_4325	Draft	3.884	43.2	166	3614	BJKP01
Ma_NIES_44	Draft	4.565	43.2	79	4278	BBPA01
**Ma_NIES_843**	**Complete**	**5.843**	**42.3**	**1**	**5595**	**NC_010296.1**
Ma_NIES_87	Draft	4.947	42.9	206	4522	BFAC01
Ma_NIES_88	Draft	5.263	43.0	29	4991	JXYX01
Ma_NIES_98	Draft	4.984	42.4	497	4647	MDZH01
Ma_OC_H_19870700_S124	Draft	4.358	43.3	213	4084	SFBR01
Ma_OC_LR_19540900_S633	Draft	4.728	42.6	231	4421	SFBQ01
**Ma_PCC7806SL**	**Complete**	**5.139**	**42.1**	**1**	**4858**	**NZ_CP020771.1**
Ma_PCC7941	Draft	4.802	42.6	77	4432	CAIK01
Ma_PCC9432	Draft	4.995	42.5	132	4652	CAIH01
Ma_PCC9443	Draft	5.185	42.7	221	4763	CAIJ01
Ma_PCC9701	Draft	4.756	42.7	323	4402	CAIQ01
Ma_PCC9717	Draft	5.300	42.7	264	4800	CAII01
Ma_PCC9806	Draft	4.205	43.1	93	3924	CAIL01
Ma_PCC9807	Draft	5.156	42.6	267	4758	CAIM01
Ma_PCC9808	Draft	5.051	42.4	141	4738	CAIN01
Ma_PCC9809	Draft	5.011	42.8	303	4639	CAIO01
Ma_QC_C_20070703_M131	Draft	4.882	42.9	274	4619	SFCA01
Ma_QC_Ch_20071001_M135	Draft	4.744	42.7	463	4495	SFBC01
Ma_QC_Ch_20071001_S25D	Draft	4.611	42.6	395	4343	SFBE01
Ma_S11_05	Draft	4.976	42.5	622	4603	JAADBM01
Ma_SC_T_19800800_S464	Draft	4.771	42.5	240	4429	SFBL01
Ma_Sj	Draft	4.617	42.8	341	4192	BDSG01
Ma_SX13_11	Draft	5.089	42.5	805	4759	JAADBO01
Ma_TAIHU98	Draft	4.850	42.5	4	4694	ANKQ01
Ma_W11_03	Draft	4.479	42.8	257	4242	JAADBP01
Ma_W11_06	Draft	4.480	42.8	245	4260	JAADBQ01
Ma_W13_11	Draft	4.866	42.4	279	4892	JAADBR01
Ma_W13_13	Draft	4.964	42.5	595	4681	JAADBS01
Ma_W13_15	Draft	5.065	42.4	638	4786	JAADBT01
Ma_W13_16	Draft	5.005	42.4	617	4699	JAADBU01
Ma_W13_18	Draft	4.975	42.5	589	4613	JAADBV01
Mf_ FACHB_1344	Draft	5.257	42.6	243	4949	JACJSW01
Mf_QC_C_20070823_S10D	Draft	4.633	42.8	226	4267	SFAM01
Mf_QC_C_20070823_S18D	Draft	4.694	42.8	231	4367	SFBT01
Mf_QC_C_20070823_S20D	Draft	4.650	42.8	216	4288	SFAJ01
Mf_WU_F_19750830_S460	Draft	4.160	43.2	193	3873	SFAN01
Mn_MB_F_20050700_S1D	Draft	4.789	42.8	348	4581	SFAV01
**Mp_FACHB_1757**	**Complete**	**5.687**	**42.4**	**1**	**5678**	**NZ_CP011339.1**
Mp_GB_SS_20050300_S99D	Draft	4.755	42.3	319	4505	SFAF01
Mp_MB_F_20080800_S26D	Draft	4.762	42.3	326	4553	SFAH01
Mp_MB_F_20051200_S6D	Draft	4.779	42.3	336	4516	SFAB01
Mp_MB_F_20051200_S9D	Draft	4.788	42.3	326	4507	SFAD01
Msp_0824	Draft	4.103	43.0	78	3838	BDSM01
Msp_LEGE00066	Draft	4.810	42.3	383	4512	JADEVX01
**Msp_MC19**	**Complete**	**5.020**	**42.7**	**1**	**4816**	**NZ_CP020664.1**
Msp_OC_Ca_00000000_S217Cul	Draft	4.819	42.7	372	4464	SFBW01
Msp_OC_L_20101000_S702	Draft	4.800	42.7	232	4405	SFBO01
Msp_QC_C_20170808_M3Col	Draft	4.536	42.5	486	4202	SFBY01
Msp_QC_C_20170808_M9Col	Draft	4.486	43.0	343	4142	SFBZ01
Msp_T1_4	Draft	4.694	42.8	449	4359	CAIP01
Mv_FACHB_1342	Draft	4.697	51.4	138	4454	JACJSV01
**Mv_NIES_102**	**Complete**	**5.874**	**42.4**	**1**	**5747**	**NZ_AP019314.1**
Mw_FACHB_1317	Draft	5.042	42.5	248	4841	JACJQV01
Mw_FACHB_1339	Draft	4.431	43.2	605	4104	JACJPS01
Mw_MB_S_20031200_S109D	Draft	4.447	43.0	285	4246	SFAP01
Mw_QC_B_20070930_S4D	Draft	4.592	42.9	207	4309	SFAR01
Mw_QC_S_20081001_S30D	Draft	4.460	42.9	208	4199	SFAT01

§ The number of protein-coding sequences is predicted by prokka v1.14 *(69)*.

Table S2.

List of single copy core genes of *Microcystis (begin next page)*. Description of core genes identified through pangenome analysis. Each core gene was used in the core gene phylogenetic tree was a complete coding sequence and a single-copy gene. Gene names and protein products were identified via blast against NCBI non-redundant database to identify the gene.

Representative gene (Mv_NIES-102 as reference)	Used for core genome tree?	% presence	% intact & single-copy	No. BP	Name	Product
Mv_NIES-102_04444	Y	100%	100%	989	accA	Acetyl-coenzyme A carboxylase carboxyl transferase subunit alpha
Mv_NIES-102_03959	Y	100%	100%	1205	ackA	Acetate kinase
Mv_NIES-102_01950	Y	100%	100%	1034	acoA	Acetoin:2%2C6-dichlorophenolindophenol oxidoreductase subunit alpha
Mv_NIES-102_05075	Y	100%	100%	248	acpP_2	Acyl carrier protein
Mv_NIES-102_03296	Y	100%	100%	1970	acsA	Acetyl-coenzyme A synthetase
Mv_NIES-102_02735	Y	100%	100%	554	act	Methanol dehydrogenase activator
Mv_NIES-102_02366	Y	100%	100%	2693	adhE	Aldehyde-alcohol dehydrogenase
Mv_NIES-102_03088	Y	100%	100%	566	adk	Adenylate kinase
Mv_NIES-102_02457	Y	100%	100%	1277	ahcY	Adenosylhomocysteinase
Mv_NIES-102_03877	Y	100%	100%	1376	aldH1	4%2C4'-diaponeurosporen-aldehyde dehydrogenase
Mv_NIES-102_05469	Y	100%	100%	1061	algA_3	Alginate biosynthesis protein AlgA
Mv_NIES-102_04253	Y	100%	100%	1652	alsS	Acetolactate synthase
Mv_NIES-102_02065	Y	100%	100%	1397	amiB2	Putative amidase AmiB2
Mv_NIES-102_04276	Y	100%	100%	1316	amiC	Aliphatic amidase expression-regulating protein
Mv_NIES-102_03978	Y	100%	100%	1241	ampG	Anhydromuropeptide permease
Mv_NIES-102_01546	Y	100%	100%	1436	amtB_1	Ammonia channel
Mv_NIES-102_01959	Y	100%	100%	1373	ankX	Phosphocholine transferase AnkX
Mv_NIES-102_03188	Y	100%	100%	662	apaH_1	Bis(5'-nucleosyl)-tetraphosphatase%2C symmetrical
Mv_NIES-102_04740	Y	100%	100%	764	apaH_2	Bis(5'-nucleosyl)-tetraphosphatase%2C symmetrical
Mv_NIES-102_00119	Y	100%	100%	1061	apbC	Iron-sulfur cluster carrier protein
Mv_NIES-102_04644	Y	100%	100%	485	apcA	Allophycocyanin alpha chain
Mv_NIES-102_04643	Y	100%	100%	485	apcB	Allophycocyanin beta chain
Mv_NIES-102_04642	Y	100%	100%	203	apcC	Phycobilisome 7.8 kDa linker polypeptide%2C allophycocyanin-associated%2C core
Mv_NIES-102_05684	Y	100%	100%	593	apcD_2	Phycobiliprotein beta chain
Mv_NIES-102_02370	Y	100%	100%	2705	apcE	Phycobiliprotein ApcE
Mv_NIES-102_01346	Y	100%	100%	1571	apnhaP	Na(+)/H(+) antiporter ApNhaP
Mv_NIES-102_00203	Y	100%	100%	1733	appA	Oligopeptide-binding protein AppA
Mv_NIES-102_01607	Y	100%	100%	518	apt	Adenine phosphoribosyltransferase
Mv_NIES-102_05140	Y	100%	100%	1283	argD	Acetylornithine aminotransferase
Mv_NIES-102_02795	Y	100%	100%	926	argF	Ornithine carbamoyltransferase
Mv_NIES-102_03890	Y	100%	100%	1205	argG	Argininosuccinate synthase
Mv_NIES-102_00761	Y	100%	100%	1757	argS	Arginine--tRNA ligase
Mv_NIES-102_01439	Y	100%	100%	1625	arnT	Undecaprenyl phosphate-alpha-4-amino-4-deoxy-L-arabinose arabinosyl transferase
Mv_NIES-102_05115	Y	100%	100%	1340	aroA1	3-phosphoshikimate 1-carboxyvinyltransferase 1
Mv_NIES-102_00298	Y	100%	100%	1100	aroB	3-dehydroquinate synthase
Mv_NIES-102_05457	Y	100%	100%	1100	aroC	Chorismate synthase
Mv_NIES-102_01323	Y	100%	100%	860	aroE	Shikimate dehydrogenase (NADP(+))
Mv_NIES-102_05475	Y	100%	100%	401	aroH	Chorismate mutase AroH
Mv_NIES-102_05673	Y	100%	100%	572	aroK	Shikimate kinase
Mv_NIES-102_01268	Y	100%	100%	1187	arsA_1	Arsenical pump-driving ATPase
Mv_NIES-102_01317	Y	100%	100%	1901	arsA_2	Arsenical pump-driving ATPase
Mv_NIES-102_03932	Y	100%	100%	398	arsC	Glutaredoxin arsenate reductase
Mv_NIES-102_03933	Y	100%	100%	647	arsH_1	NADPH-dependent quinone reductase ArsH
Mv_NIES-102_03968	Y	100%	100%	542	arsH_2	NADPH-dependent quinone reductase ArsH
Mv_NIES-102_03935	Y	100%	100%	338	arsR	Arsenical resistance operon repressor
Mv_NIES-102_00657	Y	100%	100%	1166	aspC_1	Aspartate aminotransferase
Mv_NIES-102_01106	Y	100%	100%	1163	aspC_2	Aspartate aminotransferase
Mv_NIES-102_01272	Y	100%	100%	1787	aspS	Aspartate--tRNA ligase
Mv_NIES-102_02451	Y	100%	100%	1508	atpA	ATP synthase subunit alpha

Representative gene (Mv_NIES-102 as reference)	Used for core genome tree?	% presence	% intact & single-copy	No. BP	Name	Product
Mv_NIES-102_03754	Y	100%	100%	416	atpC	ATP synthase epsilon chain
Mv_NIES-102_03753	Y	100%	100%	1448	atpD	ATP synthase subunit beta
Mv_NIES-102_02449	Y	100%	100%	542	atpF	ATP synthase subunit b
Mv_NIES-102_02452	Y	100%	100%	947	atpG_2	ATP synthase gamma chain
Mv_NIES-102_02450	Y	100%	100%	548	atpH	ATP synthase subunit delta
Mv_NIES-102_03594	Y	100%	100%	794	aviRb	23S rRNA (uridine(2479)-2'-O)-methyltransferase
Mv_NIES-102_01458	Y	100%	100%	2075	bamA_1	Outer membrane protein assembly factor BamA
Mv_NIES-102_02329	Y	100%	100%	1073	bchI	Magnesium-chelatase 38 kDa subunit
Mv_NIES-102_01642	Y	100%	100%	455	bcp_1	Putative peroxiredoxin bcp
Mv_NIES-102_01989	Y	100%	100%	467	bcp_2	Putative peroxiredoxin bcp
Mv_NIES-102_02742	Y	100%	100%	704	bcsA	Cellulose synthase catalytic subunit [UDP-forming]
Mv_NIES-102_01174	Y	100%	100%	3302	bepE	Efflux pump membrane transporter BepE
Mv_NIES-102_02178	Y	100%	100%	1601	besA	L-propargylglycine--L-glutamate ligase
Mv_NIES-102_04223	Y	100%	100%	983	bfmBAB	2-oxoisovalerate dehydrogenase subunit beta
Mv_NIES-102_03685	Y	100%	100%	1094	bioB	Biotin synthase
Mv_NIES-102_05412	Y	100%	100%	1148	bshA	N-acetyl-alpha-D-glucosaminyl L-malate synthase
Mv_NIES-102_00429	Y	100%	100%	893	btuD_2	Vitamin B12 import ATP-binding protein BtuD
Mv_NIES-102_01770	Y	100%	100%	755	btuD_3	Vitamin B12 import ATP-binding protein BtuD
Mv_NIES-102_03284	Y	100%	100%	2015	btuD_8	Vitamin B12 import ATP-binding protein BtuD
Mv_NIES-102_05020	Y	100%	100%	728	cah	Carbonic anhydrase
Mv_NIES-102_02476	Y	100%	100%	3245	carB	Carbamoyl-phosphate synthase large chain
Mv_NIES-102_00463	Y	100%	100%	914	carC_1	2-hydroxy-6-oxo-6-(2'-aminophenyl)hexa-2%2C4-dienoic acid hydrolase
Mv_NIES-102_05476	Y	100%	100%	893	carC_2	2-hydroxy-6-oxo-6-(2'-aminophenyl)hexa-2%2C4-dienoic acid hydrolase
Mv_NIES-102_02233	Y	100%	100%	1415	cbbL	Ribulose bisphosphate carboxylase large chain
Mv_NIES-102_02231	Y	100%	100%	335	cbbS	Ribulose bisphosphate carboxylase small chain
Mv_NIES-102_04263	Y	100%	100%	1376	cbiA	Cobyrinate a%2Cc-diamide synthase
Mv_NIES-102_03429	Y	100%	100%	782	cbiO	Cobalt import ATP-binding protein CbiO
Mv_NIES-102_04204	Y	100%	100%	710	cbiX	Sirohydrochlorin cobaltochelatase
Mv_NIES-102_05161	Y	100%	100%	875	cbpA	Curved DNA-binding protein
Mv_NIES-102_02237	Y	100%	100%	341	ccmK1	Carbon dioxide-concentrating mechanism protein CcmK
Mv_NIES-102_02238	Y	100%	100%	311	ccmK2	Carbon dioxide-concentrating mechanism protein CcmK
Mv_NIES-102_02236	Y	100%	100%	311	ccmL	Carbon dioxide concentrating mechanism protein CcmL
Mv_NIES-102_03870	Y	100%	100%	986	ccsA	Cytochrome c biogenesis protein CcsA
Mv_NIES-102_00226	Y	100%	100%	920	cdaA	Cyclic di-AMP synthase CdaA
Mv_NIES-102_02442	Y	100%	100%	1133	cdr	Coenzyme A disulfide reductase
Mv_NIES-102_02270	Y	100%	100%	1214	cefD_1	Isopenicillin N epimerase
Mv_NIES-102_02777	Y	100%	100%	1526	chlB	Light-independent protochlorophyllide reductase subunit B
Mv_NIES-102_05183	Y	100%	100%	869	chlL	Light-independent protochlorophyllide reductase iron-sulfur ATP-binding protein
Mv_NIES-102_02919	Y	100%	100%	692	chlM	Magnesium-protoporphyrin O-methyltransferase
Mv_NIES-102_05185	Y	100%	100%	1400	chlN	Light-independent protochlorophyllide reductase subunit N
Mv_NIES-102_03673	Y	100%	100%	1640	cimA	(R)-citramalate synthase
Mv_NIES-102_05244	Y	100%	100%	1253	cinA	Putative competence-damage inducible protein
Mv_NIES-102_01408	Y	100%	100%	2591	clcA	H(+)/Cl(-) exchange transporter ClcA
Mv_NIES-102_01463	Y	100%	100%	2723	clpB1_1	Chaperone protein ClpB 1
Mv_NIES-102_00282	Y	100%	100%	2387	clpC_1	ATP-dependent Clp protease ATP-binding subunit ClpC
Mv_NIES-102_03634	Y	100%	100%	701	clpP_3	ATP-dependent Clp protease proteolytic subunit
Mv_NIES-102_02639	Y	100%	100%	287	clpS_1	ATP-dependent Clp protease adapter protein ClpS
Mv_NIES-102_04352	Y	100%	100%	293	clpS_2	ATP-dependent Clp protease adapter protein ClpS
Mv_NIES-102_03635	Y	100%	100%	1334	clpX	ATP-dependent Clp protease ATP-binding subunit ClpX

Representative gene (Mv_NIES-102 as reference)	Used for core genome tree?	% presence	% intact & single-copy	No. BP	Name	Product
Mv_NIES-102_05063	Y	100%	100%	827	cmpB_2	Bicarbonate transport system permease protein CmpB
Mv_NIES-102_05062	Y	100%	100%	2006	cmpC_1	Bicarbonate transport ATP-binding protein CmpC
Mv_NIES-102_05542	Y	100%	100%	2018	cmpC_2	Bicarbonate transport ATP-binding protein CmpC
Mv_NIES-102_05061	Y	100%	100%	998	cmpD_2	Bicarbonate transport ATP-binding protein CmpD
Mv_NIES-102_05543	Y	100%	100%	839	cmpD_3	Bicarbonate transport ATP-binding protein CmpD
Mv_NIES-102_05090	Y	100%	100%	944	cmpR_2	HTH-type transcriptional activator CmpR
Mv_NIES-102_05482	Y	100%	100%	995	cmpR_3	HTH-type transcriptional activator CmpR
Mv_NIES-102_04326	Y	100%	100%	473	coaD	Phosphopantetheine adenylyltransferase
Mv_NIES-102_01092	Y	100%	100%	980	cobD_1	Cobalamin biosynthesis protein CobD
Mv_NIES-102_01684	Y	100%	100%	1115	cobD_2	Threonine-phosphate decarboxylase
Mv_NIES-102_01885	Y	100%	100%	3641	cobN_1	Aerobic cobaltochelatase subunit CobN
Mv_NIES-102_04817	Y	100%	100%	3986	cobN_2	Aerobic cobaltochelatase subunit CobN
Mv_NIES-102_03078	Y	100%	100%	1475	cobQ	Cobyric acid synthase
Mv_NIES-102_01644	Y	100%	100%	1643	cocE	Cocaine esterase
Mv_NIES-102_05165	Y	100%	100%	725	comB	putative 2-phosphosulfolactate phosphatase
Mv_NIES-102_05298	Y	100%	100%	1526	comM	Competence protein ComM
Mv_NIES-102_00219	Y	100%	100%	1157	corA_1	Cobalt/magnesium transport protein CorA
Mv_NIES-102_02087	Y	100%	100%	1160	corA_2	Cobalt/magnesium transport protein CorA
Mv_NIES-102_00150	Y	100%	100%	488	cpcA	C-phycocyanin alpha chain
Mv_NIES-102_00149	Y	100%	100%	518	cpcB	C-phycocyanin beta chain
Mv_NIES-102_00152	Y	100%	100%	878	cpcC1	Phycobilisome 32.1 kDa linker polypeptide%2C phycocyanin-associated%2C rod 1
Mv_NIES-102_00442	Y	100%	100%	812	cpcE	Phycocyanobilin lyase subunit alpha
Mv_NIES-102_00433	Y	100%	100%	647	cpcF	Phycocyanobilin lyase subunit beta
Mv_NIES-102_02278	Y	100%	100%	752	cpcG	Phycobilisome rod-core linker polypeptide CpcG
Mv_NIES-102_03986	Y	100%	100%	554	cpcS_2	Chromophore lyase CpcS/CpeS
Mv_NIES-102_04378	Y	100%	100%	584	cpcT1	Phycocyanobilin lyase CpcT
Mv_NIES-102_04652	Y	100%	100%	602	cpcT2_1	Phycocyanobilin lyase CpcT
Mv_NIES-102_02100	Y	100%	100%	1136	cpdA_2	3'%2C5'-cyclic adenosine monophosphate phosphodiesterase CpdA
Mv_NIES-102_01859	Y	100%	100%	539	cpeS2	Putative phycocyanobilin lyase CpcS 2
Mv_NIES-102_00413	Y	100%	100%	980	cphB_1	Cyanophycinase
Mv_NIES-102_00712	Y	100%	100%	2168	cpoB_1	Cell division coordinator CpoB
Mv_NIES-102_04133	Y	100%	100%	389	crcB_1	Putative fluoride ion transporter CrcB
Mv_NIES-102_04134	Y	100%	100%	404	crcB_2	Putative fluoride ion transporter CrcB
Mv_NIES-102_03035	Y	100%	100%	806	crnA	Creatinine amidohydrolase
Mv_NIES-102_04900	Y	100%	100%	932	crtB	15-cis-phytoene synthase
Mv_NIES-102_04249	Y	100%	100%	1505	crtN	4%2C4'-diapophytoene desaturase (4%2C4'-diapolycopene-forming)
Mv_NIES-102_04834	Y	100%	100%	1511	crtNb	4%2C4'-diapolycopene oxygenase
Mv_NIES-102_00015	Y	100%	100%	1262	csd	putative cysteine desulfurase
Mv_NIES-102_00003	Y	100%	100%	920	ctaA	Heme A synthase
Mv_NIES-102_00004	Y	100%	100%	965	ctaC	Cytochrome c oxidase subunit 2
Mv_NIES-102_00005	Y	100%	100%	1676	ctaD	Cytochrome c oxidase subunit 1
Mv_NIES-102_00006	Y	100%	100%	623	ctaE	putative cytochrome c oxidase subunit 3
Mv_NIES-102_00812	Y	100%	100%	1238	ctpA	Carboxy-terminal processing protease CtpA
Mv_NIES-102_01571	Y	100%	100%	2525	cugP_1	UTP--glucose-1-phosphate uridylyltransferase
Mv_NIES-102_03688	Y	100%	100%	1073	cugP_3	UTP--glucose-1-phosphate uridylyltransferase
Mv_NIES-102_02371	Y	100%	100%	338	cutA	Divalent-cation tolerance protein CutA
Mv_NIES-102_04656	Y	100%	100%	437	cynS	Cyanate hydratase
Mv_NIES-102_00711	Y	100%	100%	554	cysC	putative adenylyl-sulfate kinase
Mv_NIES-102_00966	Y	100%	100%	773	cysE	Serine acetyltransferase

Representative gene (Mv_NIES-102 as reference)	Used for core genome tree?	% presence	% intact & single-copy	No. BP	Name	Product
Mv_NIES-102_04870	Y	100%	100%	821	cysH	Phosphoadenosine phosphosulfate reductase
Mv_NIES-102_03387	Y	100%	100%	959	cysK1_2	O-acetylserine sulfhydrylase
Mv_NIES-102_05266	Y	100%	100%	275	cysO	Sulfur carrier protein CysO
Mv_NIES-102_04297	Y	100%	100%	812	cysQ_1	3'(2')%2C5'-bisphosphate nucleotidase CysQ
Mv_NIES-102_04861	Y	100%	100%	875	cysQ_2	3'(2')%2C5'-bisphosphate nucleotidase CysQ
Mv_NIES-102_00479	Y	100%	100%	1442	cysS	Cysteine--tRNA ligase
Mv_NIES-102_00726	Y	100%	100%	842	cysT	Sulfate transport system permease protein CysT
Mv_NIES-102_00725	Y	100%	100%	842	cysW	Sulfate transport system permease protein CysW
Mv_NIES-102_02095	Y	100%	100%	1424	dac	D-alanyl-D-alanine carboxypeptidase
Mv_NIES-102_05044	Y	100%	100%	830	dapB	4-hydroxy-tetrahydrodipicolinate reductase
Mv_NIES-102_03316	Y	100%	100%	905	dapF	Diaminopimelate epimerase
Mv_NIES-102_01935	Y	100%	100%	1187	dapL_1	LL-diaminopimelate aminotransferase
Mv_NIES-102_02111	Y	100%	100%	1235	dapL_2	LL-diaminopimelate aminotransferase
Mv_NIES-102_03357	Y	100%	100%	692	ddpX	D-alanyl-D-alanine dipeptidase
Mv_NIES-102_03743	Y	100%	100%	1358	der_2	GTPase Der
Mv_NIES-102_04206	Y	100%	100%	1364	der_3	GTPase Der
Mv_NIES-102_01034	Y	100%	100%	1037	desA_1	Delta(12)-fatty-acid desaturase
Mv_NIES-102_02752	Y	100%	100%	1040	desA_2	Delta(12)-fatty-acid desaturase
Mv_NIES-102_05477	Y	100%	100%	842	dhaA	Haloalkane dehalogenase
Mv_NIES-102_02822	Y	100%	100%	1508	dinG	3'-5' exonuclease DinG
Mv_NIES-102_03356	Y	100%	100%	404	divK	Polar-differentiation response regulator DivK
Mv_NIES-102_01453	Y	100%	100%	2624	dnaE	DNA polymerase III subunit alpha
Mv_NIES-102_01302	Y	100%	100%	1337	dnaE2	Error-prone DNA polymerase
Mv_NIES-102_01981	Y	100%	100%	1880	dnaG	DNA primase
Mv_NIES-102_01257	Y	100%	100%	1265	dnaJ_2	Chaperone protein DnaJ
Mv_NIES-102_02377	Y	100%	100%	1124	dnaJ_3	Chaperone protein DnaJ
Mv_NIES-102_04028	Y	100%	100%	689	dnaJ_4	Chaperone protein DnaJ
Mv_NIES-102_04062	Y	100%	100%	770	dnaJ_5	Chaperone protein DnaJ
Mv_NIES-102_04630	Y	100%	100%	494	dnaJ_6	Chaperone protein DnaJ
Mv_NIES-102_05510	Y	100%	100%	1007	dnaJ_7	Chaperone protein DnaJ
Mv_NIES-102_05242	Y	100%	100%	1595	dnaK_2	Chaperone protein DnaK
Mv_NIES-102_04964	Y	100%	100%	1904	dnaK2_1	Chaperone protein dnaK2
Mv_NIES-102_05160	Y	100%	100%	2162	dnaK2_2	Chaperone protein dnaK2
Mv_NIES-102_05440	Y	100%	100%	914	dpnM_4	Modification methylase DpnIIA
Mv_NIES-102_02062	Y	100%	100%	1616	dppA	Periplasmic dipeptide transport protein
Mv_NIES-102_01608	Y	100%	100%	1061	dppB_1	Dipeptide transport system permease protein DppB
Mv_NIES-102_00206	Y	100%	100%	728	dsbD	Thiol:disulfide interchange protein DsbD
Mv_NIES-102_02828	Y	100%	100%	779	dtd3	D-aminoacyl-tRNA deacylase
Mv_NIES-102_00451	Y	100%	100%	1070	dusA	tRNA-dihydrouridine(20/20a) synthase
Mv_NIES-102_02462	Y	100%	100%	1172	dxr	1-deoxy-D-xylulose 5-phosphate reductoisomerase
Mv_NIES-102_03551	Y	100%	100%	1901	dxs_1	1-deoxy-D-xylulose-5-phosphate synthase
Mv_NIES-102_05460	Y	100%	100%	668	ecfA1	Energy-coupling factor transporter ATP-binding protein EcfA1
Mv_NIES-102_03428	Y	100%	100%	773	ecfT_1	Energy-coupling factor transporter transmembrane protein EcfT
Mv_NIES-102_03744	Y	100%	100%	875	ecfT_2	Energy-coupling factor transporter transmembrane protein EcfT
Mv_NIES-102_00506	Y	100%	100%	920	efeU	Ferrous iron permease EfeU
Mv_NIES-102_01753	Y	100%	100%	1355	egtB_6	Hercynine oxygenase
Mv_NIES-102_01752	Y	100%	100%	1010	egtD	Histidine N-alpha-methyltransferase
Mv_NIES-102_01085	Y	100%	100%	1298	eno	Enolase
Mv_NIES-102_02632	Y	100%	100%	551	estB_1	Carboxylesterase 2

Representative gene (Mv_NIES-102 as reference)	Used for core genome tree?	% presence	% intact & single-copy	No. BP	Name	Product
Mv_NIES-102_04435	Y	100%	100%	578	estB_2	Extracellular esterase EstB
Mv_NIES-102_04016	Y	100%	100%	1889	ettA	Energy-dependent translational throttle protein EttA
Mv_NIES-102_04379	Y	100%	100%	800	exoA	Exodeoxyribonuclease
Mv_NIES-102_05492	Y	100%	100%	926	fabD	Malonyl CoA-acyl carrier protein transacylase
Mv_NIES-102_01978	Y	100%	100%	1124	fabF_1	3-oxoacyl-[acyl-carrier-protein] synthase 2
Mv_NIES-102_05076	Y	100%	100%	1250	fabF_2	3-oxoacyl-[acyl-carrier-protein] synthase 2
Mv_NIES-102_00958	Y	100%	100%	764	fabG_1	3-oxoacyl-[acyl-carrier-protein] reductase
Mv_NIES-102_05493	Y	100%	100%	1001	fabH	3-oxoacyl-[acyl-carrier-protein] synthase 3
Mv_NIES-102_03862	Y	100%	100%	776	fabI	Enoyl-[acyl-carrier-protein] reductase [NADH] FabI
Mv_NIES-102_03181	Y	100%	100%	479	fabZ	3-hydroxyacyl-[acyl-carrier-protein] dehydratase FabZ
Mv_NIES-102_01943	Y	100%	100%	1136	fbiC_1	FO synthase
Mv_NIES-102_04199	Y	100%	100%	887	fbiC_2	FO synthase
Mv_NIES-102_00093	Y	100%	100%	1046	fbp	Fructose-1%2C6-bisphosphatase class 1
Mv_NIES-102_00612	Y	100%	100%	938	fcl	GDP-L-fucose synthase
Mv_NIES-102_00811	Y	100%	100%	1079	fda	Fructose-bisphosphate aldolase
Mv_NIES-102_00191	Y	100%	100%	776	fetB	putative iron export permease protein FetB
Mv_NIES-102_02761	Y	100%	100%	1436	ffh	Signal recognition particle protein
Mv_NIES-102_05647	Y	100%	100%	1109	flhA	S-(hydroxymethyl)glutathione dehydrogenase
Mv_NIES-102_02687	Y	100%	100%	977	fmt	Methionyl-tRNA formyltransferase
Mv_NIES-102_03969	Y	100%	100%	356	folB	Dihydroneopterin aldolase
Mv_NIES-102_02134	Y	100%	100%	896	folD	Bifunctional protein FolD protein
Mv_NIES-102_01164	Y	100%	100%	698	folE_1	GTP cyclohydrolase 1
Mv_NIES-102_04205	Y	100%	100%	464	folK	2-amino-4-hydroxy-6-hydroxymethyldihydropteridine pyrophosphokinase
Mv_NIES-102_04323	Y	100%	100%	842	folP	Dihydropteroate synthase
Mv_NIES-102_03183	Y	100%	100%	476	fosB	Metallothiol transferase FosB
Mv_NIES-102_05164	Y	100%	100%	1346	fpgS	Folylpolyglutamate synthase
Mv_NIES-102_03782	Y	100%	100%	224	fprB	putative ferredoxin/ferredoxin--NADP reductase
Mv_NIES-102_02995	Y	100%	100%	1727	frdA	Fumarate reductase flavoprotein subunit
Mv_NIES-102_05438	Y	100%	100%	389	frp	Fluorescence recovery protein
Mv_NIES-102_00661	Y	100%	100%	548	frr	Ribosome-recycling factor
Mv_NIES-102_02546	Y	100%	100%	359	ftrC	Ferredoxin-thioredoxin reductase%2C catalytic chain
Mv_NIES-102_03786	Y	100%	100%	236	ftrV	Ferredoxin-thioredoxin reductase%2C variable chain
Mv_NIES-102_00346	Y	100%	100%	1511	ftsH_1	ATP-dependent zinc metalloprotease FtsH
Mv_NIES-102_01318	Y	100%	100%	1844	ftsH_3	ATP-dependent zinc metalloprotease FtsH
Mv_NIES-102_05026	Y	100%	100%	1895	ftsH1	ATP-dependent zinc metalloprotease FtsH 1
Mv_NIES-102_02553	Y	100%	100%	1886	ftsH2	ATP-dependent zinc metalloprotease FtsH 2
Mv_NIES-102_04057	Y	100%	100%	1853	ftsH3	ATP-dependent zinc metalloprotease FtsH 3
Mv_NIES-102_03575	Y	100%	100%	1877	ftsH4_1	ATP-dependent zinc metalloprotease FtsH 4
Mv_NIES-102_03871	Y	100%	100%	1964	ftsH4_2	ATP-dependent zinc metalloprotease FtsH 4
Mv_NIES-102_05253	Y	100%	100%	1187	ftsW	putative peptidoglycan glycosyltransferase FtsW
Mv_NIES-102_04144	Y	100%	100%	1334	ftsY	Signal recognition particle receptor FtsY
Mv_NIES-102_04334	Y	100%	100%	1403	fumC	Fumarate hydratase class II
Mv_NIES-102_01801	Y	100%	100%	2075	fusA_1	Elongation factor G
Mv_NIES-102_02965	Y	100%	100%	2033	fusA_2	Elongation factor G
Mv_NIES-102_03611	Y	100%	100%	998	galE	UDP-glucose 4-epimerase
Mv_NIES-102_01065	Y	100%	100%	1034	gap1	Glyceraldehyde-3-phosphate dehydrogenase 1
Mv_NIES-102_02093	Y	100%	100%	1352	garB	Glutathione amide reductase
Mv_NIES-102_04293	Y	100%	100%	869	garR_2	2-hydroxy-3-oxopropionate reductase
Mv_NIES-102_04065	Y	100%	100%	1472	gatB	Aspartyl/glutamyl-tRNA(Asn/Gln) amidotransferase subunit B

Representative gene (Mv_NIES-102 as reference)	Used for core genome tree?	% presence	% intact & single-copy	No. BP	Name	Product
Mv_NIES-102_03005	Y	100%	100%	803	gatD	Lipid II isoglutaminyl synthase (glutamine-hydrolyzing) subunit GatD
Mv_NIES-102_04462	Y	100%	100%	1322	gdhA	Glutamate dehydrogenase
Mv_NIES-102_00262	Y	100%	100%	1109	gldA	Glycerol dehydrogenase
Mv_NIES-102_02096	Y	100%	100%	1478	glgA_1	Glycogen synthase
Mv_NIES-102_04280	Y	100%	100%	1427	glgA_2	Glycogen synthase
Mv_NIES-102_02790	Y	100%	100%	2279	glgB	1%2C4-alpha-glucan branching enzyme GlgB
Mv_NIES-102_00449	Y	100%	100%	1289	glgC	Glucose-1-phosphate adenylyltransferase
Mv_NIES-102_01083	Y	100%	100%	2078	glgX_1	Glycogen operon protein GlgX
Mv_NIES-102_00383	Y	100%	100%	1046	glk	Glucokinase
Mv_NIES-102_02350	Y	100%	100%	1439	glmM	Phosphoglucosamine mutase
Mv_NIES-102_00375	Y	100%	100%	1901	glmS	Glutamine--fructose-6-phosphate aminotransferase [isomerizing]
Mv_NIES-102_03338	Y	100%	100%	1358	glmU	Bifunctional protein GlmU
Mv_NIES-102_04539	Y	100%	100%	2171	glnA_1	Glutamine synthetase
Mv_NIES-102_05472	Y	100%	100%	1421	glnA_2	Glutamine synthetase
Mv_NIES-102_03108	Y	100%	100%	338	glnB	Nitrogen regulatory protein P-II
Mv_NIES-102_00339	Y	100%	100%	755	glnQ_1	Glutamine transport ATP-binding protein GlnQ
Mv_NIES-102_01177	Y	100%	100%	749	glnQ_2	Glutamine transport ATP-binding protein GlnQ
Mv_NIES-102_03303	Y	100%	100%	728	glnQ_3	Glutamine transport ATP-binding protein GlnQ
Mv_NIES-102_04186	Y	100%	100%	1139	gltA	Citrate synthase
Mv_NIES-102_05072	Y	100%	100%	1484	gltB_2	Glutamate synthase [NADPH] small chain
Mv_NIES-102_01186	Y	100%	100%	899	gltI_1	Glutamate/aspartate import solute-binding protein
Mv_NIES-102_04060	Y	100%	100%	857	gltI_4	Glutamate/aspartate import solute-binding protein
Mv_NIES-102_00538	Y	100%	100%	4604	gltS_1	Ferredoxin-dependent glutamate synthase 2
Mv_NIES-102_02664	Y	100%	100%	1442	gltX	Glutamate--tRNA ligase
Mv_NIES-102_01421	Y	100%	100%	1283	glyA	Serine hydroxymethyltransferase
Mv_NIES-102_01797	Y	100%	100%	875	glyQ	Glycine--tRNA ligase alpha subunit
Mv_NIES-102_02331	Y	100%	100%	2132	glyS	Glycine--tRNA ligase beta subunit
Mv_NIES-102_03993	Y	100%	100%	953	gmd_2	GDP-mannose 4%2C6-dehydratase
Mv_NIES-102_04785	Y	100%	100%	554	gmk	Guanylate kinase
Mv_NIES-102_03490	Y	100%	100%	749	gph_3	Phosphoglycolate phosphatase
Mv_NIES-102_04077	Y	100%	100%	722	gph_4	Phosphoglycolate phosphatase
Mv_NIES-102_04251	Y	100%	100%	1337	gpmA	2%2C3-bisphosphoglycerate-dependent phosphoglycerate mutase
Mv_NIES-102_00803	Y	100%	100%	1598	gpmI	2%2C3-bisphosphoglycerate-independent phosphoglycerate mutase
Mv_NIES-102_00695	Y	100%	100%	929	gpsA	Glycerol-3-phosphate dehydrogenase [NAD(P)+]
Mv_NIES-102_02876	Y	100%	100%	494	gpt	Xanthine phosphoribosyltransferase
Mv_NIES-102_02077	Y	100%	100%	1625	groL1	60 kDa chaperonin 1
Mv_NIES-102_04015	Y	100%	100%	1682	groL2	60 kDa chaperonin 2
Mv_NIES-102_02076	Y	100%	100%	311	groS	10 kDa chaperonin
Mv_NIES-102_04078	Y	100%	100%	722	grpE	Protein GrpE
Mv_NIES-102_02781	Y	100%	100%	323	grxD	Glutaredoxin 4
Mv_NIES-102_02718	Y	100%	100%	1625	gsiA_2	Glutathione import ATP-binding protein GsiA
Mv_NIES-102_00204	Y	100%	100%	956	gsiC	Glutathione transport system permease protein GsiC
Mv_NIES-102_05275	Y	100%	100%	1625	guaA	GMP synthase [glutamine-hydrolyzing]
Mv_NIES-102_03354	Y	100%	100%	872	gyrA_1	DNA gyrase subunit A
Mv_NIES-102_03355	Y	100%	100%	1772	gyrA_2	DNA gyrase subunit A
Mv_NIES-102_04125	Y	100%	100%	2552	gyrA_3	DNA gyrase subunit A
Mv_NIES-102_05278	Y	100%	100%	1262	haeIIIM	Modification methylase HaeIII
Mv_NIES-102_04333	Y	100%	100%	2327	hcf136	Ycf48-like protein
Mv_NIES-102_04568	Y	100%	100%	971	hdfR_2	HTH-type transcriptional regulator HdfR

Representative gene (Mv_NIES-102 as reference)	Used for core genome tree?	% presence	% intact & single-copy	No. BP	Name	Product
Mv_NIES-102_04907	Y	100%	100%	2927	helY	putative helicase HelY
Mv_NIES-102_00622	Y	100%	100%	1286	hemA	Glutamyl-tRNA reductase
Mv_NIES-102_01525	Y	100%	100%	986	hemB_1	Delta-aminolevulinic acid dehydratase
Mv_NIES-102_00708	Y	100%	100%	1052	hemE	Uroporphyrinogen decarboxylase
Mv_NIES-102_04316	Y	100%	100%	1022	hemF	Oxygen-dependent coproporphyrinogen-III oxidase
Mv_NIES-102_01014	Y	100%	100%	1139	hemH_1	Ferrochelatase
Mv_NIES-102_04590	Y	100%	100%	1163	hemH_2	Ferrochelatase
Mv_NIES-102_01405	Y	100%	100%	1235	hemL_2	Glutamate-1-semialdehyde 2%2C1-aminomutase
Mv_NIES-102_03670	Y	100%	100%	971	hepT	Heptaprenyl diphosphate synthase component 2
Mv_NIES-102_03009	Y	100%	100%	995	hhoB	Putative serine protease HhoB
Mv_NIES-102_02837	Y	100%	100%	761	hisA	1-(5-phosphoribosyl)-5-[(5-phosphoribosylamino)methylideneamino] imidazole-4-carboxamide isomerase
Mv_NIES-102_00461	Y	100%	100%	1046	hisC	Histidinol-phosphate aminotransferase
Mv_NIES-102_05235	Y	100%	100%	1256	hisD_1	Histidinol dehydrogenase
Mv_NIES-102_05665	Y	100%	100%	1304	hisD_2	Histidinol dehydrogenase
Mv_NIES-102_02696	Y	100%	100%	764	hisF	Imidazole glycerol phosphate synthase subunit HisF
Mv_NIES-102_03489	Y	100%	100%	650	hisG	ATP phosphoribosyltransferase
Mv_NIES-102_03700	Y	100%	100%	638	hisH	Imidazole glycerol phosphate synthase subunit HisH
Mv_NIES-102_02002	Y	100%	100%	1298	hom	Homoserine dehydrogenase
Mv_NIES-102_03161	Y	100%	100%	716	hoxU	NAD-reducing hydrogenase HoxS subunit gamma
Mv_NIES-102_03158	Y	100%	100%	548	hoxY	NAD-reducing hydrogenase HoxS subunit delta
Mv_NIES-102_04752	Y	100%	100%	599	hpf	Ribosome hibernation promotion factor
Mv_NIES-102_02763	Y	100%	100%	914	hslO	33 kDa chaperonin
Mv_NIES-102_02043	Y	100%	100%	461	hspA	Spore protein SP21
Mv_NIES-102_01544	Y	100%	100%	2042	htpG	Chaperone protein HtpG
Mv_NIES-102_01988	Y	100%	100%	848	htpX_1	Protease HtpX
Mv_NIES-102_02722	Y	100%	100%	965	htpX_3	Protease HtpX
Mv_NIES-102_01685	Y	100%	100%	284	hup	DNA-binding protein HU
Mv_NIES-102_03535	Y	100%	100%	2264	hypF	Carbamoyltransferase HypF
Mv_NIES-102_03618	Y	100%	100%	1436	icd	Isocitrate dehydrogenase [NADP]
Mv_NIES-102_04729	Y	100%	100%	2858	ileS	Isoleucine--tRNA ligase
Mv_NIES-102_00653	Y	100%	100%	1511	ilvA	L-threonine dehydratase biosynthetic IlvA
Mv_NIES-102_02499	Y	100%	100%	1853	ilvB1	Acetolactate synthase large subunit IlvB1
Mv_NIES-102_00503	Y	100%	100%	1685	ilvD	Dihydroxy-acid dehydratase
Mv_NIES-102_01440	Y	100%	100%	812	ilvE_1	Branched-chain-amino-acid aminotransferase
Mv_NIES-102_05400	Y	100%	100%	512	IMPDH_2	Inosine-5'-monophosphate dehydrogenase
Mv_NIES-102_05022	Y	100%	100%	3032	infB	Translation initiation factor IF-2
Mv_NIES-102_03557	Y	100%	100%	989	iolG_1	Inositol 2-dehydrogenase/D-chiro-inositol 3-dehydrogenase
Mv_NIES-102_02609	Y	100%	100%	356	iscA	Iron-binding protein IscA
Mv_NIES-102_01090	Y	100%	100%	443	iscR	HTH-type transcriptional regulator IscR
Mv_NIES-102_04131	Y	100%	100%	941	ispE	4-diphosphocytidyl-2-C-methyl-D-erythritol kinase
Mv_NIES-102_00764	Y	100%	100%	479	ispF	2-C-methyl-D-erythritol 2%2C4-cyclodiphosphate synthase
Mv_NIES-102_00462	Y	100%	100%	1220	ispG	4-hydroxy-3-methylbut-2-en-1-yl diphosphate synthase (ferredoxin)
Mv_NIES-102_05180	Y	100%	100%	1208	ispH	4-hydroxy-3-methylbut-2-enyl diphosphate reductase
Mv_NIES-102_00746	Y	100%	100%	929	kaiA	Circadian clock protein KaiA
Mv_NIES-102_00747	Y	100%	100%	314	kaiB_1	Circadian clock protein KaiB
Mv_NIES-102_05149	Y	100%	100%	746	kaiB_3	Circadian clock protein KaiB
Mv_NIES-102_00748	Y	100%	100%	1562	kaiC_1	Circadian clock protein kinase KaiC
Mv_NIES-102_01451	Y	100%	100%	1520	kaiC_2	Circadian clock protein kinase KaiC
Mv_NIES-102_01229	Y	100%	100%	1643	kdc	Alpha-keto-acid decarboxylase

Representative gene (Mv_NIES-102 as reference)	Used for core genome tree?	% presence	% intact & single-copy	No. BP	Name	Product
Mv_NIES-102_05410	Y	100%	100%	689	ktrA	Ktr system potassium uptake protein A
Mv_NIES-102_05411	Y	100%	100%	1334	ktrB	Ktr system potassium uptake protein B
Mv_NIES-102_00699	Y	100%	100%	881	lapB_1	Lipopolysaccharide assembly protein B
Mv_NIES-102_03271	Y	100%	100%	1688	lapB_2	Lipopolysaccharide assembly protein B
Mv_NIES-102_04755	Y	100%	100%	1181	larC	Pyridinium-3%2C5-bisthiocarboxylic acid mononucleotide nickel insertion protein
Mv_NIES-102_03881	Y	100%	100%	809	larE	Pyridinium-3%2C5-bisthiocarboxylic acid mononucleotide synthase
Mv_NIES-102_03426	Y	100%	100%	743	larMN	putative fused nickel transport protein LarMN
Mv_NIES-102_04196	Y	100%	100%	995	ldh	L-lactate dehydrogenase
Mv_NIES-102_01531	Y	100%	100%	1811	lepA	Elongation factor 4
Mv_NIES-102_03689	Y	100%	100%	1085	leuB	3-isopropylmalate dehydrogenase
Mv_NIES-102_02714	Y	100%	100%	1433	leuC	3-isopropylmalate dehydratase large subunit
Mv_NIES-102_01244	Y	100%	100%	590	leuD1	3-isopropylmalate dehydratase small subunit 1
Mv_NIES-102_02036	Y	100%	100%	2552	leuS	Leucine--tRNA ligase
Mv_NIES-102_01358	Y	100%	100%	614	lexA	Transcription regulator LexA
Mv_NIES-102_03555	Y	100%	100%	851	lgt	Phosphatidylglycerol--prolipoprotein diacylglyceryl transferase
Mv_NIES-102_05515	Y	100%	100%	632	liaR	Transcriptional regulatory protein LiaR
Mv_NIES-102_02779	Y	100%	100%	884	lipA2	Lipoyl synthase 2
Mv_NIES-102_01742	Y	100%	100%	701	livF_1	High-affinity branched-chain amino acid transport ATP-binding protein LivF
Mv_NIES-102_04272	Y	100%	100%	698	livF_3	High-affinity branched-chain amino acid transport ATP-binding protein LivF
Mv_NIES-102_03556	Y	100%	100%	950	livH_1	High-affinity branched-chain amino acid transport system permease protein LivH
Mv_NIES-102_03898	Y	100%	100%	863	livH_2	High-affinity branched-chain amino acid transport system permease protein LivH
Mv_NIES-102_01768	Y	100%	100%	875	lnrL_1	Linearmycin resistance ATP-binding protein LnrL
Mv_NIES-102_05591	Y	100%	100%	941	lnrL_3	Linearmycin resistance ATP-binding protein LnrL
Mv_NIES-102_00284	Y	100%	100%	1526	lnt	Apolipoprotein N-acyltransferase
Mv_NIES-102_05391	Y	100%	100%	638	lon2	Lon protease 2
Mv_NIES-102_02543	Y	100%	100%	728	lptB_1	Lipopolysaccharide export system ATP-binding protein LptB
Mv_NIES-102_03699	Y	100%	100%	752	lptB_2	Lipopolysaccharide export system ATP-binding protein LptB
Mv_NIES-102_04273	Y	100%	100%	746	lptB_3	Lipopolysaccharide export system ATP-binding protein LptB
Mv_NIES-102_02539	Y	100%	100%	2393	lptD_1	LPS-assembly protein LptD
Mv_NIES-102_01436	Y	100%	100%	836	lpxA	Acyl-[acyl-carrier-protein]--UDP-N-acetylglucosamine O-acyltransferase
Mv_NIES-102_01437	Y	100%	100%	1229	lpxB_1	Lipid-A-disaccharide synthase
Mv_NIES-102_01982	Y	100%	100%	1238	lpxB_2	Lipid-A-disaccharide synthase
Mv_NIES-102_01459	Y	100%	100%	812	lpxC	UDP-3-O-acyl-N-acetylglucosamine deacetylase
Mv_NIES-102_01708	Y	100%	100%	590	lpxD_2	UDP-3-O-(3-hydroxymyristoyl)glucosamine N-acyltransferase
Mv_NIES-102_05053	Y	100%	100%	1031	lpxD_3	UDP-3-O-acylglucosamine N-acyltransferase
Mv_NIES-102_02110	Y	100%	100%	833	lpxG	UDP-2%2C3-diacylglucosamine pyrophosphatase LpxG
Mv_NIES-102_03683	Y	100%	100%	467	lspA	Lipoprotein signal peptidase
Mv_NIES-102_00227	Y	100%	100%	1403	lysA	Diaminopimelate decarboxylase
Mv_NIES-102_00145	Y	100%	100%	1220	macA	Macrolide export protein MacA
Mv_NIES-102_05207	Y	100%	100%	1214	macB_2	Macrolide export ATP-binding/permease protein MacB
Mv_NIES-102_05376	Y	100%	100%	764	map_2	Methionine aminopeptidase
Mv_NIES-102_04581	Y	100%	100%	977	mdh	Malate dehydrogenase
Mv_NIES-102_00698	Y	100%	100%	1478	mdtA_1	Multidrug resistance protein MdtA
Mv_NIES-102_01276	Y	100%	100%	1460	mdtA_2	Multidrug resistance protein MdtA
Mv_NIES-102_01613	Y	100%	100%	1457	mdtA_3	Multidrug resistance protein MdtA
Mv_NIES-102_00044	Y	100%	100%	3047	mdtC_1	Multidrug resistance protein MdtC
Mv_NIES-102_01278	Y	100%	100%	3065	mdtC_2	Multidrug resistance protein MdtC
Mv_NIES-102_01130	Y	100%	100%	911	menA_1	1%2C4-dihydroxy-2-naphthoate octaprenyltransferase
Mv_NIES-102_05714	Y	100%	100%	980	menA_2	1%2C4-dihydroxy-2-naphthoate octaprenyltransferase

Representative gene (Mv_NIES-102 as reference)	Used for core genome tree?	% presence	% intact & single-copy	No. BP	Name	Product
Mv_NIES-102_02058	Y	100%	100%	833	menB	1%2C4-dihydroxy-2-naphthoyl-CoA synthase
Mv_NIES-102_03204	Y	100%	100%	1373	menE_3	2-succinylbenzoate--CoA ligase
Mv_NIES-102_01328	Y	100%	100%	698	menG	Demethylmenaquinone methyltransferase
Mv_NIES-102_03445	Y	100%	100%	827	menH_1	2-succinyl-6-hydroxy-2%2C4-cyclohexadiene-1-carboxylate synthase
Mv_NIES-102_01774	Y	100%	100%	1385	merA_1	Mercuric reductase
Mv_NIES-102_05777	Y	100%	100%	1547	merA_2	Mercuric reductase
Mv_NIES-102_01808	Y	100%	100%	1613	metG	Methionine--tRNA ligase
Mv_NIES-102_05663	Y	100%	100%	3563	metH	Methionine synthase
Mv_NIES-102_02067	Y	100%	100%	1430	metK	S-adenosylmethionine synthase
Mv_NIES-102_05610	Y	100%	100%	3482	mfd	Transcription-repair-coupling factor
Mv_NIES-102_03231	Y	100%	100%	1235	MgdE	Monoglucosyldiacylglycerol epimerase
Mv_NIES-102_04353	Y	100%	100%	1133	mgtA	GDP-mannose-dependent alpha-mannosyltransferase
Mv_NIES-102_04058	Y	100%	100%	947	miaA	tRNA dimethylallyltransferase
Mv_NIES-102_02240	Y	100%	100%	1340	miaB_1	tRNA-2-methylthio-N(6)-dimethylallyladenosine synthase
Mv_NIES-102_01426	Y	100%	100%	833	minC	septum site-determining protein MinC
Mv_NIES-102_01425	Y	100%	100%	800	minD	Septum site-determining protein MinD
Mv_NIES-102_01424	Y	100%	100%	287	minE	Cell division topological specificity factor
Mv_NIES-102_01120	Y	100%	100%	779	mkl	putative ribonucleotide transport ATP-binding protein mkl
Mv_NIES-102_04803	Y	100%	100%	794	mlaE_2	Intermembrane phospholipid transport system permease protein MlaE
Mv_NIES-102_03964	Y	100%	100%	1091	mmpA	Metalloprotease MmpA
Mv_NIES-102_04242	Y	100%	100%	1118	mnaA	UDP-N-acetylglucosamine 2-epimerase
Mv_NIES-102_02313	Y	100%	100%	1373	mnmE_1	tRNA modification GTPase MnmE
Mv_NIES-102_04222	Y	100%	100%	1568	mnmE_2	tRNA modification GTPase MnmE
Mv_NIES-102_04145	Y	100%	100%	800	mntB_1	Manganese transport system ATP-binding protein MntB
Mv_NIES-102_00268	Y	100%	100%	572	mntP	putative manganese efflux pump MntP
Mv_NIES-102_03038	Y	100%	100%	1016	moaA_1	GTP 3'%2C8-cyclase
Mv_NIES-102_04632	Y	100%	100%	977	moaA_3	GTP 3'%2C8-cyclase
Mv_NIES-102_01704	Y	100%	100%	503	moaB	Molybdenum cofactor biosynthesis protein B
Mv_NIES-102_04458	Y	100%	100%	443	moaE	Molybdopterin synthase catalytic subunit
Mv_NIES-102_01258	Y	100%	100%	614	mobA	putative molybdenum cofactor guanylyltransferase
Mv_NIES-102_00473	Y	100%	100%	800	mprA_2	Response regulator MprA
Mv_NIES-102_03697	Y	100%	100%	656	mprA_3	Response regulator MprA
Mv_NIES-102_03758	Y	100%	100%	1067	mraY	Phospho-N-acetylmuramoyl-pentapeptide-transferase
Mv_NIES-102_04959	Y	100%	100%	1931	mrcA	Penicillin-binding protein 1A
Mv_NIES-102_05646	Y	100%	100%	1874	mrdA	Peptidoglycan D%2CD-transpeptidase MrdA
Mv_NIES-102_00118	Y	100%	100%	1280	mrdB	Peptidoglycan glycosyltransferase MrdB
Mv_NIES-102_01442	Y	100%	100%	1037	mreB	Cell shape-determining protein MreB
Mv_NIES-102_01529	Y	100%	100%	1814	msbA	Lipid A export ATP-binding/permease protein MsbA
Mv_NIES-102_05456	Y	100%	100%	1202	mshA_11	D-inositol-3-phosphate glycosyltransferase
Mv_NIES-102_05612	Y	100%	100%	1097	mshA_12	D-inositol-3-phosphate glycosyltransferase
Mv_NIES-102_05106	Y	100%	100%	1070	mshA_8	D-inositol-3-phosphate glycosyltransferase
Mv_NIES-102_05288	Y	100%	100%	662	msrA_2	Peptide methionine sulfoxide reductase MsrA
Mv_NIES-102_05774	Y	100%	100%	983	msrP_2	Protein-methionine-sulfoxide reductase catalytic subunit MsrP
Mv_NIES-102_01445	Y	100%	100%	2609	mtgA_1	Biosynthetic peptidoglycan transglycosylase
Mv_NIES-102_02102	Y	100%	100%	551	mtnD	Acireductone dioxygenase
Mv_NIES-102_04913	Y	100%	100%	1160	mtnW	2%2C3-diketo-5-methylthiopentyl-1-phosphate enolase
Mv_NIES-102_02534	Y	100%	100%	632	mtnX	2-hydroxy-3-keto-5-methylthiopentenyl-1-phosphate phosphatase
Mv_NIES-102_03592	Y	100%	100%	1325	murAA	UDP-N-acetylglucosamine 1-carboxyvinyltransferase 1
Mv_NIES-102_00194	Y	100%	100%	878	murB	UDP-N-acetylenolpyruvoylglucosamine reductase

Representative gene (Mv_NIES-102 as reference)	Used for core genome tree?	% presence	% intact & single-copy	No. BP	Name	Product
Mv_NIES-102_00193	Y	100%	100%	1427	murC	UDP-N-acetylmuramate--L-alanine ligase
Mv_NIES-102_04584	Y	100%	100%	1349	murD	UDP-N-acetylmuramoylalanine--D-glutamate ligase
Mv_NIES-102_02747	Y	100%	100%	1490	murE	UDP-N-acetylmuramoyl-L-alanyl-D-glutamate--2%2C6-diaminopimelate ligase
Mv_NIES-102_00026	Y	100%	100%	1352	murF	UDP-N-acetylmuramoyl-tripeptide--D-alanyl-D-alanine ligase
Mv_NIES-102_05694	Y	100%	100%	1073	murG	UDP-N-acetylglucosamine--N-acetylmuramyl-(pentapeptide) pyrophosphoryl-undecaprenol N-acetylglucosamine transferase
Mv_NIES-102_02926	Y	100%	100%	851	murI	Glutamate racemase
Mv_NIES-102_04438	Y	100%	100%	920	murQ	N-acetylmuramic acid 6-phosphate etherase
Mv_NIES-102_03006	Y	100%	100%	1337	murT	Lipid II isoglutaminyl synthase (glutamine-hydrolyzing) subunit MurT
Mv_NIES-102_00208	Y	100%	100%	857	mutM	Formamidopyrimidine-DNA glycosylase
Mv_NIES-102_00050	Y	100%	100%	2369	mutS2	Endonuclease MutS2
Mv_NIES-102_00881	Y	100%	100%	959	nadA	Quinolinate synthase A
Mv_NIES-102_01202	Y	100%	100%	1667	nadB	L-aspartate oxidase
Mv_NIES-102_05402	Y	100%	100%	848	nadC	putative nicotinate-nucleotide pyrophosphorylase [carboxylating]
Mv_NIES-102_01070	Y	100%	100%	566	nadD	putative nicotinate-nucleotide adenylyltransferase
Mv_NIES-102_01068	Y	100%	100%	1658	nadE	Glutamine-dependent NAD(+) synthetase
Mv_NIES-102_04318	Y	100%	100%	917	nadK_2	NAD kinase
Mv_NIES-102_02784	Y	100%	100%	2192	narB	Nitrate reductase
Mv_NIES-102_02174	Y	100%	100%	800	nasF	Uroporphyrinogen-III C-methyltransferase
Mv_NIES-102_03014	Y	100%	100%	1118	ndhA	NAD(P)H-quinone oxidoreductase subunit 1
Mv_NIES-102_02960	Y	100%	100%	1541	ndhB_2	NAD(P)H-quinone oxidoreductase subunit 2
Mv_NIES-102_05091	Y	100%	100%	1853	ndhB_3	NAD(P)H-quinone oxidoreductase subunit 2%2C chloroplastic
Mv_NIES-102_04776	Y	100%	100%	362	ndhC	NAD(P)H-quinone oxidoreductase subunit 3
Mv_NIES-102_01743	Y	100%	100%	1661	ndhD1_1	NAD(P)H-quinone oxidoreductase chain 4 1
Mv_NIES-102_03203	Y	100%	100%	1472	ndhD1_2	NAD(P)H-quinone oxidoreductase chain 4 1
Mv_NIES-102_05093	Y	100%	100%	1550	ndhD1_4	NAD(P)H-quinone oxidoreductase chain 4 1
Mv_NIES-102_03013	Y	100%	100%	572	ndhI	NAD(P)H-quinone oxidoreductase subunit I
Mv_NIES-102_04778	Y	100%	100%	503	ndhJ	NAD(P)H-quinone oxidoreductase subunit J
Mv_NIES-102_04777	Y	100%	100%	743	ndhK1	NAD(P)H-quinone oxidoreductase subunit K 1
Mv_NIES-102_02663	Y	100%	100%	476	ndhN	NAD(P)H-quinone oxidoreductase subunit N
Mv_NIES-102_04582	Y	100%	100%	215	ndhO	NAD(P)H-quinone oxidoreductase subunit O
Mv_NIES-102_03458	Y	100%	100%	1868	nhaP2	K(+)/H(+) antiporter NhaP2
Mv_NIES-102_01176	Y	100%	100%	1565	nhaS1	Low-affinity Na(+)/H(+) antiporter NhaS1
Mv_NIES-102_03235	Y	100%	100%	530	nhaX_1	Stress response protein NhaX
Mv_NIES-102_02535	Y	100%	100%	818	nit1	Deaminated glutathione amidase
Mv_NIES-102_01869	Y	100%	100%	2312	nrdA	Ribonucleoside-diphosphate reductase 1 subunit alpha
Mv_NIES-102_01896	Y	100%	100%	515	nrdR	Transcriptional repressor NrdR
Mv_NIES-102_01547	Y	100%	100%	1424	nrgA	Ammonium transporter
Mv_NIES-102_03863	Y	100%	100%	677	ntcA_1	Global nitrogen regulator
Mv_NIES-102_05392	Y	100%	100%	602	ntcA_2	Global nitrogen regulator
Mv_NIES-102_03965	Y	100%	100%	656	nth	Endonuclease III
Mv_NIES-102_03012	Y	100%	100%	596	nuoJ	NADH-quinone oxidoreductase subunit J
Mv_NIES-102_00798	Y	100%	100%	2051	nuoL_2	NADH-quinone oxidoreductase subunit L
Mv_NIES-102_05024	Y	100%	100%	1193	nusA	Transcription termination/antitermination protein NusA
Mv_NIES-102_00030	Y	100%	100%	653	nusB	Transcription antitermination protein NusB
Mv_NIES-102_01214	Y	100%	100%	617	nusG	Transcription termination/antitermination protein NusG
Mv_NIES-102_03122	Y	100%	100%	1028	obg	GTPase Obg
Mv_NIES-102_05426	Y	100%	100%	1337	pabB	Aminodeoxychorismate synthase component 1
Mv_NIES-102_04902	Y	100%	100%	2780	pacL_2	Calcium-transporting ATPase
Mv_NIES-102_02557	Y	100%	100%	2330	pacS_2	putative copper-transporting ATPase PacS

Representative gene (Mv_NIES-102 as reference)	Used for core genome tree?	% presence	% intact & single-copy	No. BP	Name	Product
Mv_NIES-102_02918	Y	100%	100%	767	panB	3-methyl-2-oxobutanoate hydroxymethyltransferase
Mv_NIES-102_02816	Y	100%	100%	1541	panC/cmk	Bifunctional pantoate ligase/cytidylate kinase
Mv_NIES-102_05074	Y	100%	100%	719	pbsA1	Heme oxygenase 1
Mv_NIES-102_03876	Y	100%	100%	1409	pchA	Salicylate biosynthesis isochorismate synthase
Mv_NIES-102_02068	Y	100%	100%	2321	pcrA	ATP-dependent DNA helicase PcrA
Mv_NIES-102_03149	Y	100%	100%	1337	pcxA_2	Proton extrusion protein PcxA
Mv_NIES-102_03335	Y	100%	100%	1259	pdhC	Dihydrolipoyllysine-residue acetyltransferase component of pyruvate dehydrogenase complex
Mv_NIES-102_02712	Y	100%	100%	1460	pds_1	15-cis-phytoene desaturase
Mv_NIES-102_04901	Y	100%	100%	1430	pds_2	15-cis-phytoene desaturase
Mv_NIES-102_00220	Y	100%	100%	1028	pdxA2	D-threonate 4-phosphate dehydrogenase
Mv_NIES-102_00099	Y	100%	100%	758	pdxJ	Pyridoxine 5'-phosphate synthase
Mv_NIES-102_04905	Y	100%	100%	782	peb1A	Major cell-binding factor
Mv_NIES-102_03569	Y	100%	100%	1472	pepA	Cytosol aminopeptidase
Mv_NIES-102_04931	Y	100%	100%	1319	pepP	Xaa-Pro aminopeptidase
Mv_NIES-102_05468	Y	100%	100%	986	petA	Cytochrome f
Mv_NIES-102_05467	Y	100%	100%	539	petC_2	Cytochrome b6-f complex iron-sulfur subunit
Mv_NIES-102_04859	Y	100%	100%	1199	petH_2	Ferredoxin--NADP reductase
Mv_NIES-102_01361	Y	100%	100%	1208	pfkA	ATP-dependent 6-phosphofructokinase
Mv_NIES-102_02665	Y	100%	100%	1082	pfp	Pyrophosphate--fructose 6-phosphate 1-phosphotransferase
Mv_NIES-102_01932	Y	100%	100%	1433	pgcA	Phosphoglucomutase
Mv_NIES-102_04737	Y	100%	100%	722	pgl	6-phosphogluconolactonase
Mv_NIES-102_02259	Y	100%	100%	533	pgsA	CDP-diacylglycerol--glycerol-3-phosphate 3-phosphatidyltransferase
Mv_NIES-102_02440	Y	100%	100%	716	phaB	Acetoacetyl-CoA reductase
Mv_NIES-102_02438	Y	100%	100%	1094	phaC	Poly(3-hydroxyalkanoate) polymerase subunit PhaC
Mv_NIES-102_02439	Y	100%	100%	995	phaE	Poly(3-hydroxyalkanoate) polymerase subunit PhaE
Mv_NIES-102_02336	Y	100%	100%	854	pheA	Bifunctional chorismate mutase/prephenate dehydratase
Mv_NIES-102_00256	Y	100%	100%	1523	phrB	(6-4) photolyase
Mv_NIES-102_04080	Y	100%	100%	1100	pilT	Twitching mobility protein
Mv_NIES-102_02061	Y	100%	100%	539	pipB2_1	Secreted effector protein PipB2
Mv_NIES-102_04791	Y	100%	100%	662	pipB2_3	Secreted effector protein PipB2
Mv_NIES-102_00294	Y	100%	100%	1445	pknD_1	Serine/threonine-protein kinase PknD
Mv_NIES-102_00667	Y	100%	100%	2123	pknD_3	Serine/threonine-protein kinase PknD
Mv_NIES-102_03868	Y	100%	100%	1322	pknD_5	Serine/threonine-protein kinase PknD
Mv_NIES-102_01128	Y	100%	100%	1013	pkwA_1	putative serine/threonine-protein kinase PkwA
Mv_NIES-102_01094	Y	100%	100%	668	plsC_1	1-acyl-sn-glycerol-3-phosphate acyltransferase
Mv_NIES-102_03248	Y	100%	100%	710	plsC_2	1-acyl-sn-glycerol-3-phosphate acyltransferase
Mv_NIES-102_05494	Y	100%	100%	1055	plsX	Phosphate acyltransferase
Mv_NIES-102_05049	Y	100%	100%	656	plsY	Glycerol-3-phosphate acyltransferase
Mv_NIES-102_00136	Y	100%	100%	1313	pmt	putative dolichyl-phosphate-mannose--protein mannosyltransferase
Mv_NIES-102_02737	Y	100%	100%	2153	pnp	Polyribonucleotide nucleotidyltransferase
Mv_NIES-102_03566	Y	100%	100%	296	pntA	NAD(P) transhydrogenase subunit alpha
Mv_NIES-102_03565	Y	100%	100%	1148	pntAA	NAD(P) transhydrogenase subunit alpha part 1
Mv_NIES-102_03567	Y	100%	100%	1409	pntB	NAD(P) transhydrogenase subunit beta
Mv_NIES-102_02150	Y	100%	100%	509	ppa	Inorganic pyrophosphatase
Mv_NIES-102_03668	Y	100%	100%	695	ppaX	Pyrophosphatase PpaX
Mv_NIES-102_01422	Y	100%	100%	3056	ppc	Phosphoenolpyruvate carboxylase
Mv_NIES-102_03940	Y	100%	100%	2441	ppsA_1	Phosphoenolpyruvate synthase
Mv_NIES-102_02738	Y	100%	100%	1100	prfA	Peptide chain release factor 1
Mv_NIES-102_04586	Y	100%	100%	992	prfB	Peptide chain release factor 2

Representative gene (Mv_NIES-102 as reference)	Used for core genome tree?	% presence	% intact & single-copy	No. BP	Name	Product
Mv_NIES-102_03247	Y	100%	100%	1634	prfC	Peptide chain release factor 3
Mv_NIES-102_04828	Y	100%	100%	2051	prlC	Oligopeptidase A
Mv_NIES-102_02496	Y	100%	100%	896	prmA	Ribosomal protein L11 methyltransferase
Mv_NIES-102_02461	Y	100%	100%	1295	proA_2	Gamma-glutamyl phosphate reductase
Mv_NIES-102_05685	Y	100%	100%	1139	proB	Glutamate 5-kinase
Mv_NIES-102_02956	Y	100%	100%	875	proC	Pyrroline-5-carboxylate reductase
Mv_NIES-102_01923	Y	100%	100%	1811	proS	Proline--tRNA ligase
Mv_NIES-102_01960	Y	100%	100%	722	prpC	Protein phosphatase PrpC
Mv_NIES-102_00069	Y	100%	100%	959	prs	Ribose-phosphate pyrophosphokinase
Mv_NIES-102_01148	Y	100%	100%	599	prxU	Selenocysteine-containing peroxiredoxin PrxU
Mv_NIES-102_00718	Y	100%	100%	911	psaA_1	Manganese ABC transporter substrate-binding lipoprotein
Mv_NIES-102_02198	Y	100%	100%	2249	psaA_2	Photosystem I P700 chlorophyll a apoprotein A1
Mv_NIES-102_02199	Y	100%	100%	2213	psaB	Photosystem I P700 chlorophyll a apoprotein A2
Mv_NIES-102_03297	Y	100%	100%	245	psaC	Photosystem I iron-sulfur center
Mv_NIES-102_02176	Y	100%	100%	494	psaF	Photosystem I reaction center subunit III
Mv_NIES-102_02177	Y	100%	100%	128	psaJ	Photosystem I reaction center subunit IX
Mv_NIES-102_00771	Y	100%	100%	266	psaK1	Photosystem I reaction center subunit PsaK 1
Mv_NIES-102_01162	Y	100%	100%	266	psaK2	Photosystem I reaction center subunit PsaK 2
Mv_NIES-102_00230	Y	100%	100%	359	psb28	Photosystem II reaction center Psb28 protein
Mv_NIES-102_01683	Y	100%	100%	1382	psbC	Photosystem II CP43 reaction center protein
Mv_NIES-102_00888	Y	100%	100%	245	psbE	Cytochrome b559 subunit alpha
Mv_NIES-102_00887	Y	100%	100%	134	psbF	Cytochrome b559 subunit beta
Mv_NIES-102_00885	Y	100%	100%	119	psbJ	Photosystem II reaction center protein J
Mv_NIES-102_04790	Y	100%	100%	137	psbK	Photosystem II reaction center protein K
Mv_NIES-102_00886	Y	100%	100%	119	psbL	Photosystem II reaction center protein L
Mv_NIES-102_01930	Y	100%	100%	833	psbO	Photosystem II manganese-stabilizing polypeptide
Mv_NIES-102_01203	Y	100%	100%	407	psbU	Photosystem II 12 kDa extrinsic protein
Mv_NIES-102_04782	Y	100%	100%	485	psbV	Cytochrome c-550
Mv_NIES-102_05386	Y	100%	100%	893	pstA_3	Phosphate transport system permease protein PstA
Mv_NIES-102_05384	Y	100%	100%	797	pstB	Phosphate import ATP-binding protein PstB
Mv_NIES-102_05385	Y	100%	100%	803	pstB3_3	Phosphate import ATP-binding protein PstB 3
Mv_NIES-102_05387	Y	100%	100%	959	pstC_2	Phosphate transport system permease protein PstC
Mv_NIES-102_05388	Y	100%	100%	1121	pstS_2	Phosphate-binding protein PstS
Mv_NIES-102_04332	Y	100%	100%	626	pth	Peptidyl-tRNA hydrolase
Mv_NIES-102_00023	Y	100%	100%	1265	purD	Phosphoribosylamine--glycine ligase
Mv_NIES-102_03894	Y	100%	100%	1127	purK	N5-carboxyaminoimidazole ribonucleotide synthase
Mv_NIES-102_05118	Y	100%	100%	2270	purL	Phosphoribosylformylglycinamidine synthase subunit PurL
Mv_NIES-102_03614	Y	100%	100%	638	purN	Phosphoribosylglycinamide formyltransferase
Mv_NIES-102_03117	Y	100%	100%	716	purQ	Phosphoribosylformylglycinamidine synthase subunit PurQ
Mv_NIES-102_03116	Y	100%	100%	275	purS	Phosphoribosylformylglycinamidine synthase subunit PurS
Mv_NIES-102_00443	Y	100%	100%	1775	pyk_1	Pyruvate kinase
Mv_NIES-102_02920	Y	100%	100%	1421	pyk_2	Pyruvate kinase
Mv_NIES-102_05009	Y	100%	100%	1271	pyrC_2	Dihydroorotase
Mv_NIES-102_01360	Y	100%	100%	1025	pyrD_1	Dihydroorotate dehydrogenase
Mv_NIES-102_05162	Y	100%	100%	1148	pyrD_2	Dihydroorotate dehydrogenase (quinone)
Mv_NIES-102_02548	Y	100%	100%	647	pyrE_1	Orotate phosphoribosyltransferase
Mv_NIES-102_00662	Y	100%	100%	677	pyrH	Uridylate kinase
Mv_NIES-102_02064	Y	100%	100%	533	pyrR	Bifunctional protein PyrR
Mv_NIES-102_03272	Y	100%	100%	1097	queA	S-adenosylmethionine:tRNA ribosyltransferase-isomerase

Representative gene (Mv_NIES-102 as reference)	Used for core genome tree?	% presence	% intact & single-copy	No. BP	Name	Product
Mv_NIES-102_00267	Y	100%	100%	692	queC	7-cyano-7-deazaguanine synthase
Mv_NIES-102_00264	Y	100%	100%	416	queE	7-carboxy-7-deazaguanine synthase
Mv_NIES-102_02481	Y	100%	100%	947	queG	Epoxyqueuosine reductase
Mv_NIES-102_05727	Y	100%	100%	1526	radA	DNA repair protein RadA
Mv_NIES-102_03909	Y	100%	100%	3062	rapA	RNA polymerase-associated protein RapA
Mv_NIES-102_05070	Y	100%	100%	839	rbgA	Ribosome biogenesis GTPase A
Mv_NIES-102_00421	Y	100%	100%	998	RBKS	Ribokinase
Mv_NIES-102_01884	Y	100%	100%	2195	rcsC_4	Sensor histidine kinase RcsC
Mv_NIES-102_05689	Y	100%	100%	2009	rcsC_7	Sensor histidine kinase RcsC
Mv_NIES-102_05172	Y	100%	100%	1067	recA	Protein RecA
Mv_NIES-102_03157	Y	100%	100%	2468	recG	ATP-dependent DNA helicase RecG
Mv_NIES-102_00474	Y	100%	100%	1727	recN	DNA repair protein RecN
Mv_NIES-102_00496	Y	100%	100%	782	recO	DNA repair protein RecO
Mv_NIES-102_03751	Y	100%	100%	2111	recQ	ATP-dependent DNA helicase RecQ
Mv_NIES-102_02139	Y	100%	100%	566	recR	Recombination protein RecR
Mv_NIES-102_02716	Y	100%	100%	2297	relA	GTP pyrophosphokinase
Mv_NIES-102_04784	Y	100%	100%	269	remA	Extracellular matrix regulatory protein A
Mv_NIES-102_04314	Y	100%	100%	2300	rep	ATP-dependent DNA helicase Rep
Mv_NIES-102_03687	Y	100%	100%	875	rfbD	dTDP-4-dehydrorhamnose reductase
Mv_NIES-102_04536	Y	100%	100%	1019	rgtE	Dodecaprenyl-phosphate galacturonate synthase
Mv_NIES-102_00153	Y	100%	100%	1676	ribBA_1	Riboflavin biosynthesis protein RibBA
Mv_NIES-102_01545	Y	100%	100%	1097	ribD	Riboflavin biosynthesis protein RibD
Mv_NIES-102_02334	Y	100%	100%	680	ribD2	2%2C5-diamino-6-ribosylamino-4(3H)-pyrimidinone 5'-phosphate reductase
Mv_NIES-102_02460	Y	100%	100%	671	ribE	Riboflavin synthase
Mv_NIES-102_01221	Y	100%	100%	923	ribF	Bifunctional riboflavin kinase/FMN adenylyltransferase
Mv_NIES-102_00790	Y	100%	100%	545	ribH	6%2C7-dimethyl-8-ribityllumazine synthase
Mv_NIES-102_04842	Y	100%	100%	905	rimK	Ribosomal protein S6--L-glutamate ligase
Mv_NIES-102_00521	Y	100%	100%	1313	rimO	Ribosomal protein S12 methylthiotransferase RimO
Mv_NIES-102_05025	Y	100%	100%	470	rimP	Ribosome maturation factor RimP
Mv_NIES-102_04646	Y	100%	100%	1208	rip3_1	Putative zinc metalloprotease Rip3
Mv_NIES-102_04739	Y	100%	100%	1247	rip3_2	Putative zinc metalloprotease Rip3
Mv_NIES-102_01133	Y	100%	100%	1367	rlmCD	23S rRNA (uracil-C(5))-methyltransferase RlmCD
Mv_NIES-102_04804	Y	100%	100%	1184	rlmI	Ribosomal RNA large subunit methyltransferase I
Mv_NIES-102_04580	Y	100%	100%	1127	rlmL	Ribosomal RNA large subunit methyltransferase K/L
Mv_NIES-102_01741	Y	100%	100%	749	rlpA	Endolytic peptidoglycan transglycosylase RlpA
Mv_NIES-102_01282	Y	100%	100%	746	rluB	Ribosomal large subunit pseudouridine synthase B
Mv_NIES-102_02614	Y	100%	100%	926	rluD_1	Ribosomal large subunit pseudouridine synthase D
Mv_NIES-102_05303	Y	100%	100%	584	rluE	Ribosomal large subunit pseudouridine synthase E
Mv_NIES-102_03686	Y	100%	100%	545	rmlC	dTDP-4-dehydrorhamnose 3%2C5-epimerase
Mv_NIES-102_01610	Y	100%	100%	878	rmlD	dTDP-4-dehydrorhamnose reductase
Mv_NIES-102_00290	Y	100%	100%	629	rnd	Ribonuclease D
Mv_NIES-102_05101	Y	100%	100%	2159	rne	Ribonuclease E
Mv_NIES-102_04075	Y	100%	100%	362	rnpA	Ribonuclease P protein component
Mv_NIES-102_00095	Y	100%	100%	2252	rnr_1	Ribonuclease R
Mv_NIES-102_02793	Y	100%	100%	956	rnz	Ribonuclease Z
Mv_NIES-102_02628	Y	100%	100%	2969	rocA	1-pyrroline-5-carboxylate dehydrogenase
Mv_NIES-102_03927	Y	100%	100%	707	rpiA	Ribose-5-phosphate isomerase A
Mv_NIES-102_01216	Y	100%	100%	713	rplA	50S ribosomal protein L1
Mv_NIES-102_03104	Y	100%	100%	833	rplB	50S ribosomal protein L2

Representative gene (Mv_NIES-102 as reference)	Used for core genome tree?	% presence	% intact & single-copy	No. BP	Name	Product
Mv_NIES-102_03107	Y	100%	100%	638	rplC	50S ribosomal protein L3
Mv_NIES-102_03106	Y	100%	100%	632	rplD	50S ribosomal protein L4
Mv_NIES-102_03095	Y	100%	100%	539	rplE	50S ribosomal protein L5
Mv_NIES-102_03093	Y	100%	100%	569	rplF	50S ribosomal protein L6
Mv_NIES-102_04377	Y	100%	100%	458	rplI	50S ribosomal protein L9
Mv_NIES-102_01893	Y	100%	100%	548	rplJ	50S ribosomal protein L10
Mv_NIES-102_01215	Y	100%	100%	425	rplK	50S ribosomal protein L11
Mv_NIES-102_01894	Y	100%	100%	392	rplL	50S ribosomal protein L7/L12
Mv_NIES-102_03097	Y	100%	100%	368	rplN	50S ribosomal protein L14
Mv_NIES-102_03090	Y	100%	100%	443	rplO	50S ribosomal protein L15
Mv_NIES-102_03100	Y	100%	100%	419	rplP	50S ribosomal protein L16
Mv_NIES-102_03092	Y	100%	100%	362	rplR	50S ribosomal protein L18
Mv_NIES-102_01211	Y	100%	100%	362	rplS	50S ribosomal protein L19
Mv_NIES-102_01854	Y	100%	100%	347	rplT	50S ribosomal protein L20
Mv_NIES-102_03102	Y	100%	100%	359	rplV	50S ribosomal protein L22
Mv_NIES-102_03105	Y	100%	100%	305	rplW	50S ribosomal protein L23
Mv_NIES-102_03096	Y	100%	100%	353	rplX	50S ribosomal protein L24
Mv_NIES-102_04440	Y	100%	100%	269	rpmA	50S ribosomal protein L27
Mv_NIES-102_00770	Y	100%	100%	236	rpmB	50S ribosomal protein L28
Mv_NIES-102_01855	Y	100%	100%	203	rpmI	50S ribosomal protein L35
Mv_NIES-102_02829	Y	100%	100%	3470	rpoB	DNA-directed RNA polymerase subunit beta
Mv_NIES-102_04724	Y	100%	100%	1880	rpoC	DNA-directed RNA polymerase subunit beta'
Mv_NIES-102_04290	Y	100%	100%	857	rpsA_2	30S ribosomal protein S1
Mv_NIES-102_01990	Y	100%	100%	797	rpsB	30S ribosomal protein S2
Mv_NIES-102_03101	Y	100%	100%	728	rpsC	30S ribosomal protein S3
Mv_NIES-102_03091	Y	100%	100%	521	rpsE	30S ribosomal protein S5
Mv_NIES-102_01802	Y	100%	100%	470	rpsG	30S ribosomal protein S7
Mv_NIES-102_03094	Y	100%	100%	401	rpsH	30S ribosomal protein S8
Mv_NIES-102_02736	Y	100%	100%	302	rpsN	30S ribosomal protein S14
Mv_NIES-102_03120	Y	100%	100%	248	rpsP	30S ribosomal protein S16
Mv_NIES-102_03098	Y	100%	100%	242	rpsQ	30S ribosomal protein S17
Mv_NIES-102_03103	Y	100%	100%	281	rpsS	30S ribosomal protein S19
Mv_NIES-102_04220	Y	100%	100%	404	rsfS	Ribosomal silencing factor RsfS
Mv_NIES-102_02375	Y	100%	100%	1076	rsgA	Small ribosomal subunit biogenesis GTPase RsgA
Mv_NIES-102_04132	Y	100%	100%	806	rsmA	Ribosomal RNA small subunit methyltransferase A
Mv_NIES-102_02631	Y	100%	100%	1352	rsmB	Ribosomal RNA small subunit methyltransferase B
Mv_NIES-102_05268	Y	100%	100%	545	rsmD	Ribosomal RNA small subunit methyltransferase D
Mv_NIES-102_02755	Y	100%	100%	677	rsmG	Ribosomal RNA small subunit methyltransferase G
Mv_NIES-102_00654	Y	100%	100%	881	rsmH	Ribosomal RNA small subunit methyltransferase H
Mv_NIES-102_02541	Y	100%	100%	839	rsmI	Ribosomal RNA small subunit methyltransferase I
Mv_NIES-102_03875	Y	100%	100%	404	rsxB	Ion-translocating oxidoreductase complex subunit B
Mv_NIES-102_03774	Y	100%	100%	1607	rsxC	Ion-translocating oxidoreductase complex subunit C
Mv_NIES-102_04645	Y	100%	100%	629	ruvA	Holliday junction ATP-dependent DNA helicase RuvA
Mv_NIES-102_02695	Y	100%	100%	1076	ruvB	Holliday junction ATP-dependent DNA helicase RuvB
Mv_NIES-102_04241	Y	100%	100%	485	ruvC	Crossover junction endodeoxyribonuclease RuvC
Mv_NIES-102_05029	Y	100%	100%	1256	sasA_10	Adaptive-response sensory-kinase SasA
Mv_NIES-102_01169	Y	100%	100%	1997	sasA_2	Adaptive-response sensory-kinase SasA
Mv_NIES-102_01746	Y	100%	100%	1106	sasA_3	Adaptive-response sensory-kinase SasA
Mv_NIES-102_02422	Y	100%	100%	1349	sasA_5	Adaptive-response sensory-kinase SasA

Representative gene (Mv_NIES-102 as reference)	Used for core genome tree?	% presence	% intact & single-copy	No. BP	Name	Product
Mv_NIES-102_03446	Y	100%	100%	1154	sasA_6	Adaptive-response sensory-kinase SasA
Mv_NIES-102_03998	Y	100%	100%	1442	sasA_8	Adaptive-response sensory-kinase SasA
Mv_NIES-102_00728	Y	100%	100%	1043	sbpA_1	Sulfate-binding protein
Mv_NIES-102_04296	Y	100%	100%	518	scpB	Segregation and condensation protein B
Mv_NIES-102_00768	Y	100%	100%	2816	secA	Protein translocase subunit SecA
Mv_NIES-102_04224	Y	100%	100%	1406	secDF	Protein translocase subunit SecDF
Mv_NIES-102_01213	Y	100%	100%	236	secE	Protein translocase subunit SecE
Mv_NIES-102_03089	Y	100%	100%	1319	secY	Protein translocase subunit SecY
Mv_NIES-102_02957	Y	100%	100%	572	sepF	Cell division protein SepF
Mv_NIES-102_02497	Y	100%	100%	1577	serA	D-3-phosphoglycerate dehydrogenase
Mv_NIES-102_04912	Y	100%	100%	1283	serS	Serine--tRNA ligase
Mv_NIES-102_04354	Y	100%	100%	662	sfp	4'-phosphopantetheinyl transferase Sfp
Mv_NIES-102_05458	Y	100%	100%	719	sfsA	Sugar fermentation stimulation protein A
Mv_NIES-102_01992	Y	100%	100%	1955	shc	Squalene--hopene cyclase
Mv_NIES-102_02691	Y	100%	100%	1133	sigA2_2	RNA polymerase sigma factor SigA2
Mv_NIES-102_00073	Y	100%	100%	602	sipT_1	Signal peptidase I T
Mv_NIES-102_02339	Y	100%	100%	575	sipT_2	Signal peptidase I T
Mv_NIES-102_05286	Y	100%	100%	1937	sir_2	Sulfite reductase [ferredoxin]
Mv_NIES-102_05390	Y	100%	100%	1541	sir_3	Sulfite reductase [ferredoxin]
Mv_NIES-102_03864	Y	100%	100%	1487	smc_5	Chromosome partition protein Smc
Mv_NIES-102_04448	Y	100%	100%	3023	smc_6	Chromosome partition protein Smc
Mv_NIES-102_04721	Y	100%	100%	3530	smc_7	Chromosome partition protein Smc
Mv_NIES-102_01945	Y	100%	100%	464	smpB	SsrA-binding protein
Mv_NIES-102_02141	Y	100%	100%	2039	speA_1	Biosynthetic arginine decarboxylase
Mv_NIES-102_05033	Y	100%	100%	1439	speA_2	Arginine decarboxylase
Mv_NIES-102_04445	Y	100%	100%	947	speE	Polyamine aminopropyltransferase
Mv_NIES-102_01602	Y	100%	100%	407	speH	S-adenosylmethionine decarboxylase proenzyme
Mv_NIES-102_02657	Y	100%	100%	752	sphR	Alkaline phosphatase synthesis transcriptional regulatory protein SphR
Mv_NIES-102_02658	Y	100%	100%	1301	sphS	Sensor protein SphS
Mv_NIES-102_03449	Y	100%	100%	1700	spkA	Serine/threonine-protein kinase A
Mv_NIES-102_02749	Y	100%	100%	1586	spkB	Serine/threonine-protein kinase B
Mv_NIES-102_03237	Y	100%	100%	1814	sppA	Protease 4
Mv_NIES-102_01441	Y	100%	100%	350	ssb	Single-stranded DNA-binding protein
Mv_NIES-102_03278	Y	100%	100%	824	sseA	3-mercaptopyruvate sulfurtransferase
Mv_NIES-102_00539	Y	100%	100%	887	sucD	Succinate--CoA ligase [ADP-forming] subunit alpha
Mv_NIES-102_00018	Y	100%	100%	1442	sufB	FeS cluster assembly protein SufB
Mv_NIES-102_00016	Y	100%	100%	1325	sufD	FeS cluster assembly protein SufD
Mv_NIES-102_01255	Y	100%	100%	422	sufE	Cysteine desulfuration protein SufE
Mv_NIES-102_02834	Y	100%	100%	785	surA_2	Chaperone SurA
Mv_NIES-102_00127	Y	100%	100%	725	tagA	N-acetylglucosaminyldiphosphoundecaprenol N-acetyl-beta-D-mannosaminyltransferase
Mv_NIES-102_01420	Y	100%	100%	1064	tagO_1	putative undecaprenyl-phosphate N-acetylglucosaminyl 1-phosphate transferase
Mv_NIES-102_00042	Y	100%	100%	1388	tagU_1	Polyisoprenyl-teichoic acid--peptidoglycan teichoic acid transferase TagU
Mv_NIES-102_03976	Y	100%	100%	1412	tagU_2	Polyisoprenyl-teichoic acid--peptidoglycan teichoic acid transferase TagU
Mv_NIES-102_01856	Y	100%	100%	1175	tal	Transaldolase
Mv_NIES-102_05316	Y	100%	100%	926	tam	Trans-aconitate 2-methyltransferase
Mv_NIES-102_04244	Y	100%	100%	785	tcyN	L-cystine import ATP-binding protein TcyN
Mv_NIES-102_04789	Y	100%	100%	1124	tgt	Queuine tRNA-ribosyltransferase
Mv_NIES-102_01944	Y	100%	100%	701	thf1	Protein Thf1
Mv_NIES-102_01858	Y	100%	100%	1382	thiC	Phosphomethylpyrimidine synthase

Representative gene (Mv_NIES-102 as reference)	Used for core genome tree?	% presence	% intact & single-copy	No. BP	Name	Product
Mv_NIES-102_01779	Y	100%	100%	1016	thiE	Thiamine-phosphate synthase
Mv_NIES-102_05059	Y	100%	100%	1976	thiG	Thiazole synthase
Mv_NIES-102_02760	Y	100%	100%	968	thiL	Thiamine-monophosphate kinase
Mv_NIES-102_05263	Y	100%	100%	917	thrB_1	Homoserine kinase
Mv_NIES-102_00717	Y	100%	100%	1799	thrS_1	Threonine--tRNA ligase
Mv_NIES-102_01772	Y	100%	100%	1493	thyA	Thymidylate synthase
Mv_NIES-102_04315	Y	100%	100%	974	tilS	tRNA(Ile)-lysidine synthase
Mv_NIES-102_05077	Y	100%	100%	2006	tkt	Transketolase
Mv_NIES-102_00946	Y	100%	100%	1469	tldD_1	Metalloprotease TldD
Mv_NIES-102_02684	Y	100%	100%	806	tlyA	16S/23S rRNA (cytidine-2'-O)-methyltransferase TlyA
Mv_NIES-102_01773	Y	100%	100%	623	tmk	Thymidylate kinase
Mv_NIES-102_03582	Y	100%	100%	887	todF	2-hydroxy-6-oxo-2%2C4-heptadienoate hydrolase
Mv_NIES-102_00446	Y	100%	100%	536	tolB_1	Tol-Pal system protein TolB
Mv_NIES-102_00447	Y	100%	100%	521	tolB_2	Tol-Pal system protein TolB
Mv_NIES-102_02839	Y	100%	100%	551	tolB_3	Tol-Pal system protein TolB
Mv_NIES-102_04138	Y	100%	100%	512	tolB_6	Tol-Pal system protein TolB
Mv_NIES-102_04741	Y	100%	100%	692	tpiA	Triosephosphate isomerase
Mv_NIES-102_00412	Y	100%	100%	695	trmD	tRNA (guanine-N(1)-)-methyltransferase
Mv_NIES-102_05681	Y	100%	100%	1337	trmFO	Methylenetetrahydrofolate--tRNA-(uracil-5-)-methyltransferase TrmFO
Mv_NIES-102_05413	Y	100%	100%	800	trmJ	tRNA (cytidine/uridine-2'-O-)-methyltransferase TrmJ
Mv_NIES-102_05267	Y	100%	100%	1304	trpB_1	Tryptophan synthase beta chain
Mv_NIES-102_05328	Y	100%	100%	1247	trpB_2	Tryptophan synthase beta chain
Mv_NIES-102_04567	Y	100%	100%	1061	trpD	Anthranilate phosphoribosyltransferase
Mv_NIES-102_00036	Y	100%	100%	1520	trpE	Anthranilate synthase component 1
Mv_NIES-102_01163	Y	100%	100%	638	trpF	N-(5'-phosphoribosyl)anthranilate isomerase
Mv_NIES-102_00040	Y	100%	100%	587	trpG	Anthranilate synthase component 2
Mv_NIES-102_00135	Y	100%	100%	1010	trpS	Tryptophan--tRNA ligase
Mv_NIES-102_00459	Y	100%	100%	881	truB	tRNA pseudouridine synthase B
Mv_NIES-102_00458	Y	100%	100%	1034	tsaD	tRNA N6-adenosine threonylcarbamoyltransferase
Mv_NIES-102_01991	Y	100%	100%	755	tsf	Elongation factor Ts
Mv_NIES-102_01800	Y	100%	100%	1229	tuf	Elongation factor Tu
Mv_NIES-102_00068	Y	100%	100%	1793	typA	GTP-binding protein TypA/BipA
Mv_NIES-102_03785	Y	100%	100%	1217	tyrS	Tyrosine--tRNA ligase
Mv_NIES-102_01961	Y	100%	100%	1688	ubiB_1	protein kinase UbiB
Mv_NIES-102_02322	Y	100%	100%	1991	ubiB_2	protein kinase UbiB
Mv_NIES-102_04835	Y	100%	100%	1679	ubiB_3	putative protein kinase UbiB
Mv_NIES-102_05424	Y	100%	100%	1718	ubiB_4	protein kinase UbiB
Mv_NIES-102_05470	Y	100%	100%	1649	ubiB_5	putative protein kinase UbiB
Mv_NIES-102_04779	Y	100%	100%	1505	ubiD	3-octaprenyl-4-hydroxybenzoate carboxy-lyase
Mv_NIES-102_05188	Y	100%	100%	803	udp	Uridine phosphorylase
Mv_NIES-102_00225	Y	100%	100%	749	uppS	Ditrans%2Cpolycis-undecaprenyl-diphosphate synthase ((2E%2C6E)-farnesyl-diphosphate specific)
Mv_NIES-102_04130	Y	100%	100%	812	ureD	Urease accessory protein UreD
Mv_NIES-102_01687	Y	100%	100%	476	ureE	Urease accessory protein UreE
Mv_NIES-102_01706	Y	100%	100%	665	ureF	Urease accessory protein UreF
Mv_NIES-102_00128	Y	100%	100%	596	ureG_1	Urease accessory protein UreG
Mv_NIES-102_01543	Y	100%	100%	1502	uvrB_1	UvrABC system protein B
Mv_NIES-102_03691	Y	100%	100%	1877	uvrC	UvrABC system protein C
Mv_NIES-102_03616	Y	100%	100%	2720	valS	Valine--tRNA ligase
Mv_NIES-102_00970	Y	100%	100%	686	vte5	Phytol kinase

Representative gene (Mv_NIES-102 as reference)	Used for core genome tree?	% presence	% intact & single-copy	No. BP	Name	Product
Mv_NIES-102_05073	Y	100%	100%	740	walR_1	Transcriptional regulatory protein WalR
Mv_NIES-102_05728	Y	100%	100%	728	walR_2	Transcriptional regulatory protein WalR
Mv_NIES-102_01716	Y	100%	100%	857	wbbL	N-acetylglucosaminyl-diphospho-decaprenol L-rhamnosyltransferase
Mv_NIES-102_00292	Y	100%	100%	995	wcaJ	UDP-glucose:undecaprenyl-phosphate glucose-1-phosphate transferase
Mv_NIES-102_01660	Y	100%	100%	2198	xfp	Xylulose-5-phosphate/fructose-6-phosphate phosphoketolase
Mv_NIES-102_05304	Y	100%	100%	788	yadH	Inner membrane transport permease YadH
Mv_NIES-102_01643	Y	100%	100%	1694	ybiT	putative ABC transporter ATP-binding protein YbiT
Mv_NIES-102_00120	Y	100%	100%	791	ycbX	putative protein YcbX
Mv_NIES-102_05672	Y	100%	100%	1073	yceM	Putative oxidoreductase YceM
Mv_NIES-102_03936	Y	100%	100%	119	ycf12	Photosystem II reaction center protein Ycf12
Mv_NIES-102_00132	Y	100%	100%	782	ycf3_1	Photosystem I assembly protein Ycf3
Mv_NIES-102_00504	Y	100%	100%	866	ycf3_5	Photosystem I assembly protein Ycf3
Mv_NIES-102_00719	Y	100%	100%	1103	yciC_2	Putative metal chaperone YciC
Mv_NIES-102_00255	Y	100%	100%	341	yciH	putative protein YciH
Mv_NIES-102_04754	Y	100%	100%	641	yciO	putative protein YciO
Mv_NIES-102_05489	Y	100%	100%	650	ydjZ	TVP38/TMEM64 family inner membrane protein YdjZ
Mv_NIES-102_01067	Y	100%	100%	1013	ygfZ	tRNA-modifying protein YgfZ
Mv_NIES-102_05105	Y	100%	100%	953	yhdJ_2	DNA adenine methyltransferase YhdJ
Mv_NIES-102_01657	Y	100%	100%	959	yhdN	Aldo-keto reductase YhdN
Mv_NIES-102_01338	Y	100%	100%	995	yhdY	Inner membrane amino-acid ABC transporter permease protein YhdY
Mv_NIES-102_04949	Y	100%	100%	1742	yheI	putative multidrug resistance ABC transporter ATP-binding/permease protein YheI
Mv_NIES-102_00066	Y	100%	100%	659	yhfK	putative sugar epimerase YhfK
Mv_NIES-102_01324	Y	100%	100%	1043	yhhT_2	Putative transport protein YhhT
Mv_NIES-102_04061	Y	100%	100%	1037	yhhT_3	Putative transport protein YhhT
Mv_NIES-102_04073	Y	100%	100%	1148	yidC	Membrane protein insertase YidC
Mv_NIES-102_04320	Y	100%	100%	890	ykfA_1	putative murein peptide carboxypeptidase
Mv_NIES-102_05398	Y	100%	100%	1043	ykfA_2	putative murein peptide carboxypeptidase
Mv_NIES-102_00713	Y	100%	100%	830	ykoC	Putative HMP/thiamine permease protein YkoC
Mv_NIES-102_05116	Y	100%	100%	1715	ykoD	Putative HMP/thiamine import ATP-binding protein YkoD
Mv_NIES-102_00626	Y	100%	100%	551	yrdA	Protein YrdA
Mv_NIES-102_02469	Y	100%	100%	479	yrrK	Putative pre-16S rRNA nuclease
Mv_NIES-102_01872	Y	100%	100%	491	ywnH	Putative phosphinothricin acetyltransferase YwnH
Mv_NIES-102_05609	Y	100%	100%	947	znuA	High-affinity zinc uptake system binding-protein ZnuA
Mv_NIES-102_02682	Y	100%	100%	830	znuB	High-affinity zinc uptake system membrane protein ZnuB
Mv_NIES-102_05608	Y	100%	100%	743	znuC	High-affinity zinc uptake system ATP-binding protein ZnuC
Mv_NIES-102_03115	Y	100%	100%	383	zur	Zinc uptake regulation protein
Mv_NIES-102_01877	Y	100%	100%	1529	zwf2	Glucose-6-phosphate 1-dehydrogenase 2
Mv_NIES-102_03447	Y	100%	100%	1589		1%2C4-alpha-glucan branching enzyme
Mv_NIES-102_03232	Y	100%	100%	944		2-haloacrylate reductase
Mv_NIES-102_00787	Y	100%	100%	2696		A-adding tRNA nucleotidyltransferase
Mv_NIES-102_05287	Y	100%	100%	2399		ABC transporter ATP-binding/permease protein
Mv_NIES-102_02711	Y	100%	100%	695		Aldehyde decarbonylase
Mv_NIES-102_03230	Y	100%	100%	1427		Beta-monoglucosyldiacylglycerol synthase
Mv_NIES-102_02756	Y	100%	100%	1088		Ca(2+)/H(+) antiporter
Mv_NIES-102_05280	Y	100%	100%	1085		Ca(2+)/H(+) antiporter
Mv_NIES-102_00101	Y	100%	100%	1640		Cardiolipin synthase
Mv_NIES-102_01673	Y	100%	100%	662		Catechol O-methyltransferase
Mv_NIES-102_02536	Y	100%	100%	1217		CC-adding tRNA nucleotidyltransferase
Mv_NIES-102_00229	Y	100%	100%	947		Epimerase family protein

Representative gene (Mv_NIES-102 as reference)	Used for core genome tree?	% presence	% intact & single-copy	No. BP	Name	Product
Mv_NIES-102_00103	Y	100%	100%	860		Formylglycine-generating enzyme
Mv_NIES-102_03776	Y	100%	100%	1373		Gluconeogenesis factor
Mv_NIES-102_03291	Y	100%	100%	1190		Glycogen synthase
Mv_NIES-102_01675	Y	100%	100%	941		Homogentisate phytyltransferase
Mv_NIES-102_00014	Y	100%	100%	1505		hypothetical protein
Mv_NIES-102_00019	Y	100%	100%	638		hypothetical protein
Mv_NIES-102_00021	Y	100%	100%	1556		hypothetical protein
Mv_NIES-102_00028	Y	100%	100%	587		hypothetical protein
Mv_NIES-102_00029	Y	100%	100%	1463		hypothetical protein
Mv_NIES-102_00038	Y	100%	100%	1637		hypothetical protein
Mv_NIES-102_00039	Y	100%	100%	809		hypothetical protein
Mv_NIES-102_00043	Y	100%	100%	677		hypothetical protein
Mv_NIES-102_00067	Y	100%	100%	704		hypothetical protein
Mv_NIES-102_00094	Y	100%	100%	782		hypothetical protein
Mv_NIES-102_00096	Y	100%	100%	821		hypothetical protein
Mv_NIES-102_00106	Y	100%	100%	896		hypothetical protein
Mv_NIES-102_00109	Y	100%	100%	827		hypothetical protein
Mv_NIES-102_00111	Y	100%	100%	254		hypothetical protein
Mv_NIES-102_00125	Y	100%	100%	1601		hypothetical protein
Mv_NIES-102_00126	Y	100%	100%	1415		hypothetical protein
Mv_NIES-102_00131	Y	100%	100%	548		hypothetical protein
Mv_NIES-102_00133	Y	100%	100%	179		hypothetical protein
Mv_NIES-102_00137	Y	100%	100%	629		hypothetical protein
Mv_NIES-102_00138	Y	100%	100%	2048		hypothetical protein
Mv_NIES-102_00139	Y	100%	100%	779		hypothetical protein
Mv_NIES-102_00140	Y	100%	100%	509		hypothetical protein
Mv_NIES-102_00181	Y	100%	100%	1826		hypothetical protein
Mv_NIES-102_00205	Y	100%	100%	362		hypothetical protein
Mv_NIES-102_00213	Y	100%	100%	200		hypothetical protein
Mv_NIES-102_00214	Y	100%	100%	356		hypothetical protein
Mv_NIES-102_00215	Y	100%	100%	3326		hypothetical protein
Mv_NIES-102_00217	Y	100%	100%	983		hypothetical protein
Mv_NIES-102_00254	Y	100%	100%	467		hypothetical protein
Mv_NIES-102_00263	Y	100%	100%	557		hypothetical protein
Mv_NIES-102_00269	Y	100%	100%	953		hypothetical protein
Mv_NIES-102_00286	Y	100%	100%	386		hypothetical protein
Mv_NIES-102_00287	Y	100%	100%	665		hypothetical protein
Mv_NIES-102_00293	Y	100%	100%	452		hypothetical protein
Mv_NIES-102_00299	Y	100%	100%	1139		hypothetical protein
Mv_NIES-102_00326	Y	100%	100%	185		hypothetical protein
Mv_NIES-102_00327	Y	100%	100%	1292		hypothetical protein
Mv_NIES-102_00338	Y	100%	100%	1511		hypothetical protein
Mv_NIES-102_00345	Y	100%	100%	395		hypothetical protein
Mv_NIES-102_00347	Y	100%	100%	434		hypothetical protein
Mv_NIES-102_00349	Y	100%	100%	656		hypothetical protein
Mv_NIES-102_00358	Y	100%	100%	1175		hypothetical protein
Mv_NIES-102_00359	Y	100%	100%	527		hypothetical protein
Mv_NIES-102_00363	Y	100%	100%	410		hypothetical protein
Mv_NIES-102_00364	Y	100%	100%	257		hypothetical protein

Representative gene (Mv_NIES-102 as reference)	Used for core genome tree?	% presence	% intact & single-copy	No. BP	Name	Product
Mv_NIES-102_00422	Y	100%	100%	212		hypothetical protein
Mv_NIES-102_00427	Y	100%	100%	281		hypothetical protein
Mv_NIES-102_00428	Y	100%	100%	479		hypothetical protein
Mv_NIES-102_00430	Y	100%	100%	773		hypothetical protein
Mv_NIES-102_00445	Y	100%	100%	815		hypothetical protein
Mv_NIES-102_00448	Y	100%	100%	1097		hypothetical protein
Mv_NIES-102_00450	Y	100%	100%	1364		hypothetical protein
Mv_NIES-102_00452	Y	100%	100%	812		hypothetical protein
Mv_NIES-102_00454	Y	100%	100%	581		hypothetical protein
Mv_NIES-102_00467	Y	100%	100%	1646		hypothetical protein
Mv_NIES-102_00472	Y	100%	100%	1058		hypothetical protein
Mv_NIES-102_00480	Y	100%	100%	1529		hypothetical protein
Mv_NIES-102_00489	Y	100%	100%	779		hypothetical protein
Mv_NIES-102_00490	Y	100%	100%	527		hypothetical protein
Mv_NIES-102_00493	Y	100%	100%	1547		hypothetical protein
Mv_NIES-102_00494	Y	100%	100%	812		hypothetical protein
Mv_NIES-102_00497	Y	100%	100%	1490		hypothetical protein
Mv_NIES-102_00505	Y	100%	100%	434		hypothetical protein
Mv_NIES-102_00507	Y	100%	100%	1613		hypothetical protein
Mv_NIES-102_00520	Y	100%	100%	893		hypothetical protein
Mv_NIES-102_00614	Y	100%	100%	341		hypothetical protein
Mv_NIES-102_00616	Y	100%	100%	1055		hypothetical protein
Mv_NIES-102_00624	Y	100%	100%	497		hypothetical protein
Mv_NIES-102_00625	Y	100%	100%	566		hypothetical protein
Mv_NIES-102_00628	Y	100%	100%	1334		hypothetical protein
Mv_NIES-102_00639	Y	100%	100%	461		hypothetical protein
Mv_NIES-102_00663	Y	100%	100%	581		hypothetical protein
Mv_NIES-102_00668	Y	100%	100%	668		hypothetical protein
Mv_NIES-102_00705	Y	100%	100%	1202		hypothetical protein
Mv_NIES-102_00720	Y	100%	100%	437		hypothetical protein
Mv_NIES-102_00721	Y	100%	100%	2636		hypothetical protein
Mv_NIES-102_00727	Y	100%	100%	341		hypothetical protein
Mv_NIES-102_00729	Y	100%	100%	779		hypothetical protein
Mv_NIES-102_00731	Y	100%	100%	554		hypothetical protein
Mv_NIES-102_00749	Y	100%	100%	995		hypothetical protein
Mv_NIES-102_00750	Y	100%	100%	1097		hypothetical protein
Mv_NIES-102_00755	Y	100%	100%	170		hypothetical protein
Mv_NIES-102_00756	Y	100%	100%	1946		hypothetical protein
Mv_NIES-102_00757	Y	100%	100%	407		hypothetical protein
Mv_NIES-102_00758	Y	100%	100%	251		hypothetical protein
Mv_NIES-102_00759	Y	100%	100%	758		hypothetical protein
Mv_NIES-102_00760	Y	100%	100%	392		hypothetical protein
Mv_NIES-102_00767	Y	100%	100%	170		hypothetical protein
Mv_NIES-102_00774	Y	100%	100%	1175		hypothetical protein
Mv_NIES-102_00775	Y	100%	100%	1265		hypothetical protein
Mv_NIES-102_00785	Y	100%	100%	296		hypothetical protein
Mv_NIES-102_00786	Y	100%	100%	164		hypothetical protein
Mv_NIES-102_00795	Y	100%	100%	407		hypothetical protein
Mv_NIES-102_00804	Y	100%	100%	977		hypothetical protein

Representative gene (Mv_NIES-102 as reference)	Used for core genome tree?	% presence	% intact & single-copy	No. BP	Name	Product
Mv_NIES-102_00810	Y	100%	100%	1127		hypothetical protein
Mv_NIES-102_00813	Y	100%	100%	1304		hypothetical protein
Mv_NIES-102_00890	Y	100%	100%	347		hypothetical protein
Mv_NIES-102_00921	Y	100%	100%	1211		hypothetical protein
Mv_NIES-102_00923	Y	100%	100%	530		hypothetical protein
Mv_NIES-102_00928	Y	100%	100%	224		hypothetical protein
Mv_NIES-102_00929	Y	100%	100%	809		hypothetical protein
Mv_NIES-102_00933	Y	100%	100%	1001		hypothetical protein
Mv_NIES-102_00934	Y	100%	100%	866		hypothetical protein
Mv_NIES-102_00945	Y	100%	100%	590		hypothetical protein
Mv_NIES-102_00955	Y	100%	100%	419		hypothetical protein
Mv_NIES-102_00956	Y	100%	100%	737		hypothetical protein
Mv_NIES-102_00957	Y	100%	100%	767		hypothetical protein
Mv_NIES-102_00967	Y	100%	100%	602		hypothetical protein
Mv_NIES-102_00972	Y	100%	100%	758		hypothetical protein
Mv_NIES-102_01066	Y	100%	100%	368		hypothetical protein
Mv_NIES-102_01069	Y	100%	100%	728		hypothetical protein
Mv_NIES-102_01084	Y	100%	100%	761		hypothetical protein
Mv_NIES-102_01089	Y	100%	100%	389		hypothetical protein
Mv_NIES-102_01091	Y	100%	100%	473		hypothetical protein
Mv_NIES-102_01093	Y	100%	100%	365		hypothetical protein
Mv_NIES-102_01119	Y	100%	100%	632		hypothetical protein
Mv_NIES-102_01122	Y	100%	100%	1391		hypothetical protein
Mv_NIES-102_01131	Y	100%	100%	1694		hypothetical protein
Mv_NIES-102_01135	Y	100%	100%	281		hypothetical protein
Mv_NIES-102_01136	Y	100%	100%	401		hypothetical protein
Mv_NIES-102_01137	Y	100%	100%	638		hypothetical protein
Mv_NIES-102_01140	Y	100%	100%	356		hypothetical protein
Mv_NIES-102_01141	Y	100%	100%	1130		hypothetical protein
Mv_NIES-102_01142	Y	100%	100%	563		hypothetical protein
Mv_NIES-102_01166	Y	100%	100%	905		hypothetical protein
Mv_NIES-102_01167	Y	100%	100%	407		hypothetical protein
Mv_NIES-102_01170	Y	100%	100%	347		hypothetical protein
Mv_NIES-102_01171	Y	100%	100%	179		hypothetical protein
Mv_NIES-102_01178	Y	100%	100%	449		hypothetical protein
Mv_NIES-102_01187	Y	100%	100%	374		hypothetical protein
Mv_NIES-102_01201	Y	100%	100%	1094		hypothetical protein
Mv_NIES-102_01228	Y	100%	100%	1418		hypothetical protein
Mv_NIES-102_01231	Y	100%	100%	398		hypothetical protein
Mv_NIES-102_01235	Y	100%	100%	1514		hypothetical protein
Mv_NIES-102_01240	Y	100%	100%	1283		hypothetical protein
Mv_NIES-102_01246	Y	100%	100%	2189		hypothetical protein
Mv_NIES-102_01247	Y	100%	100%	2237		hypothetical protein
Mv_NIES-102_01256	Y	100%	100%	551		hypothetical protein
Mv_NIES-102_01262	Y	100%	100%	905		hypothetical protein
Mv_NIES-102_01263	Y	100%	100%	746		hypothetical protein
Mv_NIES-102_01264	Y	100%	100%	1214		hypothetical protein
Mv_NIES-102_01265	Y	100%	100%	2222		hypothetical protein
Mv_NIES-102_01271	Y	100%	100%	782		hypothetical protein

Representative gene (Mv_NIES-102 as reference)	Used for core genome tree?	% presence	% intact & single-copy	No. BP	Name	Product
Mv_NIES-102_01280	Y	100%	100%	848		hypothetical protein
Mv_NIES-102_01281	Y	100%	100%	761		hypothetical protein
Mv_NIES-102_01314	Y	100%	100%	260		hypothetical protein
Mv_NIES-102_01315	Y	100%	100%	332		hypothetical protein
Mv_NIES-102_01322	Y	100%	100%	377		hypothetical protein
Mv_NIES-102_01329	Y	100%	100%	1151		hypothetical protein
Mv_NIES-102_01330	Y	100%	100%	704		hypothetical protein
Mv_NIES-102_01331	Y	100%	100%	806		hypothetical protein
Mv_NIES-102_01349	Y	100%	100%	539		hypothetical protein
Mv_NIES-102_01357	Y	100%	100%	266		hypothetical protein
Mv_NIES-102_01384	Y	100%	100%	434		hypothetical protein
Mv_NIES-102_01392	Y	100%	100%	2051		hypothetical protein
Mv_NIES-102_01406	Y	100%	100%	380		hypothetical protein
Mv_NIES-102_01435	Y	100%	100%	1319		hypothetical protein
Mv_NIES-102_01443	Y	100%	100%	749		hypothetical protein
Mv_NIES-102_01444	Y	100%	100%	536		hypothetical protein
Mv_NIES-102_01523	Y	100%	100%	380		hypothetical protein
Mv_NIES-102_01524	Y	100%	100%	365		hypothetical protein
Mv_NIES-102_01572	Y	100%	100%	191		hypothetical protein
Mv_NIES-102_01599	Y	100%	100%	650		hypothetical protein
Mv_NIES-102_01600	Y	100%	100%	512		hypothetical protein
Mv_NIES-102_01601	Y	100%	100%	209		hypothetical protein
Mv_NIES-102_01605	Y	100%	100%	653		hypothetical protein
Mv_NIES-102_01606	Y	100%	100%	626		hypothetical protein
Mv_NIES-102_01612	Y	100%	100%	494		hypothetical protein
Mv_NIES-102_01614	Y	100%	100%	602		hypothetical protein
Mv_NIES-102_01656	Y	100%	100%	575		hypothetical protein
Mv_NIES-102_01659	Y	100%	100%	1499		hypothetical protein
Mv_NIES-102_01661	Y	100%	100%	935		hypothetical protein
Mv_NIES-102_01662	Y	100%	100%	1127		hypothetical protein
Mv_NIES-102_01663	Y	100%	100%	1226		hypothetical protein
Mv_NIES-102_01664	Y	100%	100%	2879		hypothetical protein
Mv_NIES-102_01686	Y	100%	100%	518		hypothetical protein
Mv_NIES-102_01707	Y	100%	100%	950		hypothetical protein
Mv_NIES-102_01709	Y	100%	100%	1751		hypothetical protein
Mv_NIES-102_01727	Y	100%	100%	965		hypothetical protein
Mv_NIES-102_01731	Y	100%	100%	1445		hypothetical protein
Mv_NIES-102_01733	Y	100%	100%	707		hypothetical protein
Mv_NIES-102_01744	Y	100%	100%	194		hypothetical protein
Mv_NIES-102_01745	Y	100%	100%	416		hypothetical protein
Mv_NIES-102_01749	Y	100%	100%	224		hypothetical protein
Mv_NIES-102_01750	Y	100%	100%	590		hypothetical protein
Mv_NIES-102_01751	Y	100%	100%	284		hypothetical protein
Mv_NIES-102_01754	Y	100%	100%	434		hypothetical protein
Mv_NIES-102_01756	Y	100%	100%	128		hypothetical protein
Mv_NIES-102_01769	Y	100%	100%	818		hypothetical protein
Mv_NIES-102_01780	Y	100%	100%	209		hypothetical protein
Mv_NIES-102_01796	Y	100%	100%	923		hypothetical protein
Mv_NIES-102_01806	Y	100%	100%	1196		hypothetical protein

Representative gene (Mv_NIES-102 as reference)	Used for core genome tree?	% presence	% intact & single-copy	No. BP	Name	Product
Mv_NIES-102_01807	Y	100%	100%	605		hypothetical protein
Mv_NIES-102_01809	Y	100%	100%	716		hypothetical protein
Mv_NIES-102_01871	Y	100%	100%	2972		hypothetical protein
Mv_NIES-102_01873	Y	100%	100%	6137		hypothetical protein
Mv_NIES-102_01874	Y	100%	100%	1190		hypothetical protein
Mv_NIES-102_01878	Y	100%	100%	1355		hypothetical protein
Mv_NIES-102_01879	Y	100%	100%	584		hypothetical protein
Mv_NIES-102_01899	Y	100%	100%	701		hypothetical protein
Mv_NIES-102_01900	Y	100%	100%	668		hypothetical protein
Mv_NIES-102_01903	Y	100%	100%	1682		hypothetical protein
Mv_NIES-102_01934	Y	100%	100%	1286		hypothetical protein
Mv_NIES-102_01946	Y	100%	100%	548		hypothetical protein
Mv_NIES-102_01948	Y	100%	100%	2306		hypothetical protein
Mv_NIES-102_01962	Y	100%	100%	305		hypothetical protein
Mv_NIES-102_01986	Y	100%	100%	290		hypothetical protein
Mv_NIES-102_01987	Y	100%	100%	419		hypothetical protein
Mv_NIES-102_02001	Y	100%	100%	935		hypothetical protein
Mv_NIES-102_02042	Y	100%	100%	1214		hypothetical protein
Mv_NIES-102_02048	Y	100%	100%	1679		hypothetical protein
Mv_NIES-102_02049	Y	100%	100%	1997		hypothetical protein
Mv_NIES-102_02050	Y	100%	100%	503		hypothetical protein
Mv_NIES-102_02051	Y	100%	100%	890		hypothetical protein
Mv_NIES-102_02054	Y	100%	100%	392		hypothetical protein
Mv_NIES-102_02059	Y	100%	100%	302		hypothetical protein
Mv_NIES-102_02063	Y	100%	100%	782		hypothetical protein
Mv_NIES-102_02069	Y	100%	100%	431		hypothetical protein
Mv_NIES-102_02086	Y	100%	100%	1277		hypothetical protein
Mv_NIES-102_02097	Y	100%	100%	800		hypothetical protein
Mv_NIES-102_02098	Y	100%	100%	626		hypothetical protein
Mv_NIES-102_02116	Y	100%	100%	788		hypothetical protein
Mv_NIES-102_02135	Y	100%	100%	737		hypothetical protein
Mv_NIES-102_02137	Y	100%	100%	419		hypothetical protein
Mv_NIES-102_02140	Y	100%	100%	533		hypothetical protein
Mv_NIES-102_02175	Y	100%	100%	419		hypothetical protein
Mv_NIES-102_02179	Y	100%	100%	239		hypothetical protein
Mv_NIES-102_02196	Y	100%	100%	566		hypothetical protein
Mv_NIES-102_02232	Y	100%	100%	398		hypothetical protein
Mv_NIES-102_02234	Y	100%	100%	662		hypothetical protein
Mv_NIES-102_02235	Y	100%	100%	1958		hypothetical protein
Mv_NIES-102_02241	Y	100%	100%	386		hypothetical protein
Mv_NIES-102_02271	Y	100%	100%	1433		hypothetical protein
Mv_NIES-102_02280	Y	100%	100%	635		hypothetical protein
Mv_NIES-102_02321	Y	100%	100%	1088		hypothetical protein
Mv_NIES-102_02333	Y	100%	100%	1190		hypothetical protein
Mv_NIES-102_02352	Y	100%	100%	1727		hypothetical protein
Mv_NIES-102_02356	Y	100%	100%	374		hypothetical protein
Mv_NIES-102_02357	Y	100%	100%	494		hypothetical protein
Mv_NIES-102_02358	Y	100%	100%	1151		hypothetical protein
Mv_NIES-102_02359	Y	100%	100%	1850		hypothetical protein

Representative gene (Mv_NIES-102 as reference)	Used for core genome tree?	% presence	% intact & single-copy	No. BP	Name	Product
Mv_NIES-102_02376	Y	100%	100%	221		hypothetical protein
Mv_NIES-102_02421	Y	100%	100%	470		hypothetical protein
Mv_NIES-102_02437	Y	100%	100%	1217		hypothetical protein
Mv_NIES-102_02456	Y	100%	100%	605		hypothetical protein
Mv_NIES-102_02468	Y	100%	100%	1250		hypothetical protein
Mv_NIES-102_02470	Y	100%	100%	1241		hypothetical protein
Mv_NIES-102_02473	Y	100%	100%	506		hypothetical protein
Mv_NIES-102_02488	Y	100%	100%	1811		hypothetical protein
Mv_NIES-102_02489	Y	100%	100%	1001		hypothetical protein
Mv_NIES-102_02507	Y	100%	100%	743		hypothetical protein
Mv_NIES-102_02537	Y	100%	100%	248		hypothetical protein
Mv_NIES-102_02542	Y	100%	100%	1169		hypothetical protein
Mv_NIES-102_02545	Y	100%	100%	425		hypothetical protein
Mv_NIES-102_02547	Y	100%	100%	299		hypothetical protein
Mv_NIES-102_02554	Y	100%	100%	215		hypothetical protein
Mv_NIES-102_02555	Y	100%	100%	965		hypothetical protein
Mv_NIES-102_02556	Y	100%	100%	341		hypothetical protein
Mv_NIES-102_02576	Y	100%	100%	668		hypothetical protein
Mv_NIES-102_02610	Y	100%	100%	389		hypothetical protein
Mv_NIES-102_02611	Y	100%	100%	341		hypothetical protein
Mv_NIES-102_02615	Y	100%	100%	1001		hypothetical protein
Mv_NIES-102_02616	Y	100%	100%	215		hypothetical protein
Mv_NIES-102_02617	Y	100%	100%	386		hypothetical protein
Mv_NIES-102_02618	Y	100%	100%	407		hypothetical protein
Mv_NIES-102_02630	Y	100%	100%	410		hypothetical protein
Mv_NIES-102_02638	Y	100%	100%	830		hypothetical protein
Mv_NIES-102_02656	Y	100%	100%	767		hypothetical protein
Mv_NIES-102_02686	Y	100%	100%	380		hypothetical protein
Mv_NIES-102_02697	Y	100%	100%	200		hypothetical protein
Mv_NIES-102_02699	Y	100%	100%	1097		hypothetical protein
Mv_NIES-102_02702	Y	100%	100%	554		hypothetical protein
Mv_NIES-102_02709	Y	100%	100%	1136		hypothetical protein
Mv_NIES-102_02713	Y	100%	100%	2438		hypothetical protein
Mv_NIES-102_02717	Y	100%	100%	356		hypothetical protein
Mv_NIES-102_02720	Y	100%	100%	2135		hypothetical protein
Mv_NIES-102_02723	Y	100%	100%	266		hypothetical protein
Mv_NIES-102_02724	Y	100%	100%	887		hypothetical protein
Mv_NIES-102_02743	Y	100%	100%	383		hypothetical protein
Mv_NIES-102_02745	Y	100%	100%	548		hypothetical protein
Mv_NIES-102_02753	Y	100%	100%	914		hypothetical protein
Mv_NIES-102_02757	Y	100%	100%	623		hypothetical protein
Mv_NIES-102_02758	Y	100%	100%	1484		hypothetical protein
Mv_NIES-102_02759	Y	100%	100%	671		hypothetical protein
Mv_NIES-102_02762	Y	100%	100%	416		hypothetical protein
Mv_NIES-102_02764	Y	100%	100%	1955		hypothetical protein
Mv_NIES-102_02775	Y	100%	100%	587		hypothetical protein
Mv_NIES-102_02776	Y	100%	100%	596		hypothetical protein
Mv_NIES-102_02778	Y	100%	100%	1127		hypothetical protein
Mv_NIES-102_02783	Y	100%	100%	1964		hypothetical protein

Representative gene (Mv_NIES-102 as reference)	Used for core genome tree?	% presence	% intact & single-copy	No. BP	Name	Product
Mv_NIES-102_02794	Y	100%	100%	3236		hypothetical protein
Mv_NIES-102_02796	Y	100%	100%	425		hypothetical protein
Mv_NIES-102_02817	Y	100%	100%	1823		hypothetical protein
Mv_NIES-102_02818	Y	100%	100%	323		hypothetical protein
Mv_NIES-102_02819	Y	100%	100%	518		hypothetical protein
Mv_NIES-102_02823	Y	100%	100%	1547		hypothetical protein
Mv_NIES-102_02824	Y	100%	100%	335		hypothetical protein
Mv_NIES-102_02832	Y	100%	100%	515		hypothetical protein
Mv_NIES-102_02838	Y	100%	100%	281		hypothetical protein
Mv_NIES-102_02874	Y	100%	100%	689		hypothetical protein
Mv_NIES-102_02875	Y	100%	100%	233		hypothetical protein
Mv_NIES-102_02925	Y	100%	100%	1898		hypothetical protein
Mv_NIES-102_02952	Y	100%	100%	347		hypothetical protein
Mv_NIES-102_02954	Y	100%	100%	536		hypothetical protein
Mv_NIES-102_02959	Y	100%	100%	272		hypothetical protein
Mv_NIES-102_03004	Y	100%	100%	920		hypothetical protein
Mv_NIES-102_03029	Y	100%	100%	785		hypothetical protein
Mv_NIES-102_03037	Y	100%	100%	1373		hypothetical protein
Mv_NIES-102_03075	Y	100%	100%	740		hypothetical protein
Mv_NIES-102_03076	Y	100%	100%	704		hypothetical protein
Mv_NIES-102_03099	Y	100%	100%	218		hypothetical protein
Mv_NIES-102_03109	Y	100%	100%	485		hypothetical protein
Mv_NIES-102_03121	Y	100%	100%	365		hypothetical protein
Mv_NIES-102_03146	Y	100%	100%	1484		hypothetical protein
Mv_NIES-102_03147	Y	100%	100%	1307		hypothetical protein
Mv_NIES-102_03150	Y	100%	100%	2120		hypothetical protein
Mv_NIES-102_03156	Y	100%	100%	893		hypothetical protein
Mv_NIES-102_03180	Y	100%	100%	1139		hypothetical protein
Mv_NIES-102_03182	Y	100%	100%	767		hypothetical protein
Mv_NIES-102_03184	Y	100%	100%	533		hypothetical protein
Mv_NIES-102_03233	Y	100%	100%	386		hypothetical protein
Mv_NIES-102_03236	Y	100%	100%	2159		hypothetical protein
Mv_NIES-102_03251	Y	100%	100%	983		hypothetical protein
Mv_NIES-102_03273	Y	100%	100%	389		hypothetical protein
Mv_NIES-102_03275	Y	100%	100%	1187		hypothetical protein
Mv_NIES-102_03276	Y	100%	100%	1175		hypothetical protein
Mv_NIES-102_03277	Y	100%	100%	551		hypothetical protein
Mv_NIES-102_03279	Y	100%	100%	443		hypothetical protein
Mv_NIES-102_03285	Y	100%	100%	1169		hypothetical protein
Mv_NIES-102_03287	Y	100%	100%	578		hypothetical protein
Mv_NIES-102_03313	Y	100%	100%	1514		hypothetical protein
Mv_NIES-102_03314	Y	100%	100%	683		hypothetical protein
Mv_NIES-102_03317	Y	100%	100%	212		hypothetical protein
Mv_NIES-102_03334	Y	100%	100%	947		hypothetical protein
Mv_NIES-102_03341	Y	100%	100%	581		hypothetical protein
Mv_NIES-102_03343	Y	100%	100%	1058		hypothetical protein
Mv_NIES-102_03375	Y	100%	100%	749		hypothetical protein
Mv_NIES-102_03386	Y	100%	100%	659		hypothetical protein
Mv_NIES-102_03427	Y	100%	100%	323		hypothetical protein

Representative gene (Mv_NIES-102 as reference)	Used for core genome tree?	% presence	% intact & single-copy	No. BP	Name	Product
Mv_NIES-102_03434	Y	100%	100%	1190		hypothetical protein
Mv_NIES-102_03442	Y	100%	100%	293		hypothetical protein
Mv_NIES-102_03443	Y	100%	100%	1055		hypothetical protein
Mv_NIES-102_03450	Y	100%	100%	914		hypothetical protein
Mv_NIES-102_03451	Y	100%	100%	1187		hypothetical protein
Mv_NIES-102_03455	Y	100%	100%	1796		hypothetical protein
Mv_NIES-102_03534	Y	100%	100%	290		hypothetical protein
Mv_NIES-102_03536	Y	100%	100%	674		hypothetical protein
Mv_NIES-102_03537	Y	100%	100%	410		hypothetical protein
Mv_NIES-102_03558	Y	100%	100%	275		hypothetical protein
Mv_NIES-102_03559	Y	100%	100%	560		hypothetical protein
Mv_NIES-102_03568	Y	100%	100%	239		hypothetical protein
Mv_NIES-102_03573	Y	100%	100%	1184		hypothetical protein
Mv_NIES-102_03610	Y	100%	100%	398		hypothetical protein
Mv_NIES-102_03667	Y	100%	100%	620		hypothetical protein
Mv_NIES-102_03671	Y	100%	100%	545		hypothetical protein
Mv_NIES-102_03684	Y	100%	100%	653		hypothetical protein
Mv_NIES-102_03690	Y	100%	100%	260		hypothetical protein
Mv_NIES-102_03694	Y	100%	100%	638		hypothetical protein
Mv_NIES-102_03695	Y	100%	100%	293		hypothetical protein
Mv_NIES-102_03698	Y	100%	100%	1115		hypothetical protein
Mv_NIES-102_03750	Y	100%	100%	611		hypothetical protein
Mv_NIES-102_03757	Y	100%	100%	263		hypothetical protein
Mv_NIES-102_03773	Y	100%	100%	509		hypothetical protein
Mv_NIES-102_03775	Y	100%	100%	485		hypothetical protein
Mv_NIES-102_03851	Y	100%	100%	650		hypothetical protein
Mv_NIES-102_03872	Y	100%	100%	1916		hypothetical protein
Mv_NIES-102_03873	Y	100%	100%	353		hypothetical protein
Mv_NIES-102_03874	Y	100%	100%	551		hypothetical protein
Mv_NIES-102_03893	Y	100%	100%	938		hypothetical protein
Mv_NIES-102_03910	Y	100%	100%	440		hypothetical protein
Mv_NIES-102_03912	Y	100%	100%	434		hypothetical protein
Mv_NIES-102_03924	Y	100%	100%	572		hypothetical protein
Mv_NIES-102_03928	Y	100%	100%	674		hypothetical protein
Mv_NIES-102_03929	Y	100%	100%	200		hypothetical protein
Mv_NIES-102_03930	Y	100%	100%	881		hypothetical protein
Mv_NIES-102_03938	Y	100%	100%	1448		hypothetical protein
Mv_NIES-102_03960	Y	100%	100%	551		hypothetical protein
Mv_NIES-102_03961	Y	100%	100%	656		hypothetical protein
Mv_NIES-102_03962	Y	100%	100%	590		hypothetical protein
Mv_NIES-102_03966	Y	100%	100%	608		hypothetical protein
Mv_NIES-102_03967	Y	100%	100%	326		hypothetical protein
Mv_NIES-102_03973	Y	100%	100%	455		hypothetical protein
Mv_NIES-102_03977	Y	100%	100%	620		hypothetical protein
Mv_NIES-102_03979	Y	100%	100%	1424		hypothetical protein
Mv_NIES-102_03994	Y	100%	100%	920		hypothetical protein
Mv_NIES-102_04017	Y	100%	100%	470		hypothetical protein
Mv_NIES-102_04027	Y	100%	100%	764		hypothetical protein
Mv_NIES-102_04059	Y	100%	100%	1406		hypothetical protein

Representative gene (Mv_NIES-102 as reference)	Used for core genome tree?	% presence	% intact & single-copy	No. BP	Name	Product
Mv_NIES-102_04066	Y	100%	100%	1658		hypothetical protein
Mv_NIES-102_04072	Y	100%	100%	494		hypothetical protein
Mv_NIES-102_04074	Y	100%	100%	395		hypothetical protein
Mv_NIES-102_04076	Y	100%	100%	1964		hypothetical protein
Mv_NIES-102_04085	Y	100%	100%	2570		hypothetical protein
Mv_NIES-102_04123	Y	100%	100%	395		hypothetical protein
Mv_NIES-102_04124	Y	100%	100%	530		hypothetical protein
Mv_NIES-102_04129	Y	100%	100%	524		hypothetical protein
Mv_NIES-102_04137	Y	100%	100%	215		hypothetical protein
Mv_NIES-102_04139	Y	100%	100%	1343		hypothetical protein
Mv_NIES-102_04146	Y	100%	100%	416		hypothetical protein
Mv_NIES-102_04147	Y	100%	100%	1280		hypothetical protein
Mv_NIES-102_04172	Y	100%	100%	1223		hypothetical protein
Mv_NIES-102_04173	Y	100%	100%	494		hypothetical protein
Mv_NIES-102_04200	Y	100%	100%	1664		hypothetical protein
Mv_NIES-102_04201	Y	100%	100%	266		hypothetical protein
Mv_NIES-102_04207	Y	100%	100%	557		hypothetical protein
Mv_NIES-102_04218	Y	100%	100%	953		hypothetical protein
Mv_NIES-102_04219	Y	100%	100%	503		hypothetical protein
Mv_NIES-102_04221	Y	100%	100%	596		hypothetical protein
Mv_NIES-102_04243	Y	100%	100%	1331		hypothetical protein
Mv_NIES-102_04252	Y	100%	100%	1247		hypothetical protein
Mv_NIES-102_04254	Y	100%	100%	479		hypothetical protein
Mv_NIES-102_04259	Y	100%	100%	674		hypothetical protein
Mv_NIES-102_04274	Y	100%	100%	1154		hypothetical protein
Mv_NIES-102_04275	Y	100%	100%	1160		hypothetical protein
Mv_NIES-102_04278	Y	100%	100%	332		hypothetical protein
Mv_NIES-102_04279	Y	100%	100%	605		hypothetical protein
Mv_NIES-102_04291	Y	100%	100%	842		hypothetical protein
Mv_NIES-102_04304	Y	100%	100%	2312		hypothetical protein
Mv_NIES-102_04307	Y	100%	100%	464		hypothetical protein
Mv_NIES-102_04308	Y	100%	100%	203		hypothetical protein
Mv_NIES-102_04309	Y	100%	100%	512		hypothetical protein
Mv_NIES-102_04310	Y	100%	100%	497		hypothetical protein
Mv_NIES-102_04313	Y	100%	100%	239		hypothetical protein
Mv_NIES-102_04319	Y	100%	100%	977		hypothetical protein
Mv_NIES-102_04324	Y	100%	100%	797		hypothetical protein
Mv_NIES-102_04325	Y	100%	100%	659		hypothetical protein
Mv_NIES-102_04327	Y	100%	100%	284		hypothetical protein
Mv_NIES-102_04331	Y	100%	100%	224		hypothetical protein
Mv_NIES-102_04351	Y	100%	100%	284		hypothetical protein
Mv_NIES-102_04355	Y	100%	100%	941		hypothetical protein
Mv_NIES-102_04380	Y	100%	100%	551		hypothetical protein
Mv_NIES-102_04381	Y	100%	100%	317		hypothetical protein
Mv_NIES-102_04382	Y	100%	100%	152		hypothetical protein
Mv_NIES-102_04383	Y	100%	100%	890		hypothetical protein
Mv_NIES-102_04408	Y	100%	100%	452		hypothetical protein
Mv_NIES-102_04414	Y	100%	100%	242		hypothetical protein
Mv_NIES-102_04415	Y	100%	100%	815		hypothetical protein

Representative gene (Mv_NIES-102 as reference)	Used for core genome tree?	% presence	% intact & single-copy	No. BP	Name	Product
Mv_NIES-102_04416	Y	100%	100%	680		hypothetical protein
Mv_NIES-102_04417	Y	100%	100%	1061		hypothetical protein
Mv_NIES-102_04418	Y	100%	100%	440		hypothetical protein
Mv_NIES-102_04425	Y	100%	100%	734		hypothetical protein
Mv_NIES-102_04437	Y	100%	100%	1490		hypothetical protein
Mv_NIES-102_04439	Y	100%	100%	389		hypothetical protein
Mv_NIES-102_04446	Y	100%	100%	2282		hypothetical protein
Mv_NIES-102_04447	Y	100%	100%	254		hypothetical protein
Mv_NIES-102_04468	Y	100%	100%	176		hypothetical protein
Mv_NIES-102_04469	Y	100%	100%	1058		hypothetical protein
Mv_NIES-102_04470	Y	100%	100%	581		hypothetical protein
Mv_NIES-102_04472	Y	100%	100%	2354		hypothetical protein
Mv_NIES-102_04473	Y	100%	100%	1568		hypothetical protein
Mv_NIES-102_04474	Y	100%	100%	464		hypothetical protein
Mv_NIES-102_04476	Y	100%	100%	629		hypothetical protein
Mv_NIES-102_04537	Y	100%	100%	875		hypothetical protein
Mv_NIES-102_04577	Y	100%	100%	719		hypothetical protein
Mv_NIES-102_04579	Y	100%	100%	350		hypothetical protein
Mv_NIES-102_04583	Y	100%	100%	2651		hypothetical protein
Mv_NIES-102_04585	Y	100%	100%	1112		hypothetical protein
Mv_NIES-102_04589	Y	100%	100%	251		hypothetical protein
Mv_NIES-102_04629	Y	100%	100%	563		hypothetical protein
Mv_NIES-102_04631	Y	100%	100%	272		hypothetical protein
Mv_NIES-102_04647	Y	100%	100%	1148		hypothetical protein
Mv_NIES-102_04653	Y	100%	100%	173		hypothetical protein
Mv_NIES-102_04654	Y	100%	100%	368		hypothetical protein
Mv_NIES-102_04655	Y	100%	100%	1811		hypothetical protein
Mv_NIES-102_04723	Y	100%	100%	1511		hypothetical protein
Mv_NIES-102_04731	Y	100%	100%	731		hypothetical protein
Mv_NIES-102_04735	Y	100%	100%	791		hypothetical protein
Mv_NIES-102_04742	Y	100%	100%	791		hypothetical protein
Mv_NIES-102_04753	Y	100%	100%	410		hypothetical protein
Mv_NIES-102_04783	Y	100%	100%	206		hypothetical protein
Mv_NIES-102_04788	Y	100%	100%	461		hypothetical protein
Mv_NIES-102_04792	Y	100%	100%	362		hypothetical protein
Mv_NIES-102_04802	Y	100%	100%	1352		hypothetical protein
Mv_NIES-102_04805	Y	100%	100%	410		hypothetical protein
Mv_NIES-102_04809	Y	100%	100%	1055		hypothetical protein
Mv_NIES-102_04860	Y	100%	100%	842		hypothetical protein
Mv_NIES-102_04903	Y	100%	100%	1799		hypothetical protein
Mv_NIES-102_04904	Y	100%	100%	536		hypothetical protein
Mv_NIES-102_04908	Y	100%	100%	953		hypothetical protein
Mv_NIES-102_04909	Y	100%	100%	509		hypothetical protein
Mv_NIES-102_04910	Y	100%	100%	962		hypothetical protein
Mv_NIES-102_04911	Y	100%	100%	602		hypothetical protein
Mv_NIES-102_04914	Y	100%	100%	1118		hypothetical protein
Mv_NIES-102_04936	Y	100%	100%	377		hypothetical protein
Mv_NIES-102_04937	Y	100%	100%	419		hypothetical protein
Mv_NIES-102_04944	Y	100%	100%	1244		hypothetical protein

Representative gene (Mv_NIES-102 as reference)	Used for core genome tree?	% presence	% intact & single-copy	No. BP	Name	Product
Mv_NIES-102_04950	Y	100%	100%	1661		hypothetical protein
Mv_NIES-102_04967	Y	100%	100%	962		hypothetical protein
Mv_NIES-102_04968	Y	100%	100%	1001		hypothetical protein
Mv_NIES-102_04969	Y	100%	100%	983		hypothetical protein
Mv_NIES-102_04970	Y	100%	100%	386		hypothetical protein
Mv_NIES-102_04972	Y	100%	100%	794		hypothetical protein
Mv_NIES-102_04973	Y	100%	100%	797		hypothetical protein
Mv_NIES-102_05021	Y	100%	100%	569		hypothetical protein
Mv_NIES-102_05023	Y	100%	100%	281		hypothetical protein
Mv_NIES-102_05028	Y	100%	100%	320		hypothetical protein
Mv_NIES-102_05030	Y	100%	100%	377		hypothetical protein
Mv_NIES-102_05031	Y	100%	100%	1076		hypothetical protein
Mv_NIES-102_05035	Y	100%	100%	353		hypothetical protein
Mv_NIES-102_05067	Y	100%	100%	941		hypothetical protein
Mv_NIES-102_05084	Y	100%	100%	245		hypothetical protein
Mv_NIES-102_05086	Y	100%	100%	1121		hypothetical protein
Mv_NIES-102_05087	Y	100%	100%	1163		hypothetical protein
Mv_NIES-102_05092	Y	100%	100%	725		hypothetical protein
Mv_NIES-102_05094	Y	100%	100%	1295		hypothetical protein
Mv_NIES-102_05104	Y	100%	100%	782		hypothetical protein
Mv_NIES-102_05110	Y	100%	100%	1340		hypothetical protein
Mv_NIES-102_05111	Y	100%	100%	164		hypothetical protein
Mv_NIES-102_05148	Y	100%	100%	1367		hypothetical protein
Mv_NIES-102_05150	Y	100%	100%	212		hypothetical protein
Mv_NIES-102_05152	Y	100%	100%	467		hypothetical protein
Mv_NIES-102_05155	Y	100%	100%	797		hypothetical protein
Mv_NIES-102_05163	Y	100%	100%	1589		hypothetical protein
Mv_NIES-102_05173	Y	100%	100%	596		hypothetical protein
Mv_NIES-102_05179	Y	100%	100%	473		hypothetical protein
Mv_NIES-102_05181	Y	100%	100%	179		hypothetical protein
Mv_NIES-102_05243	Y	100%	100%	131		hypothetical protein
Mv_NIES-102_05250	Y	100%	100%	386		hypothetical protein
Mv_NIES-102_05269	Y	100%	100%	677		hypothetical protein
Mv_NIES-102_05279	Y	100%	100%	1583		hypothetical protein
Mv_NIES-102_05281	Y	100%	100%	461		hypothetical protein
Mv_NIES-102_05282	Y	100%	100%	308		hypothetical protein
Mv_NIES-102_05291	Y	100%	100%	1652		hypothetical protein
Mv_NIES-102_05292	Y	100%	100%	2051		hypothetical protein
Mv_NIES-102_05299	Y	100%	100%	1304		hypothetical protein
Mv_NIES-102_05300	Y	100%	100%	428		hypothetical protein
Mv_NIES-102_05311	Y	100%	100%	2750		hypothetical protein
Mv_NIES-102_05315	Y	100%	100%	653		hypothetical protein
Mv_NIES-102_05317	Y	100%	100%	1196		hypothetical protein
Mv_NIES-102_05320	Y	100%	100%	1262		hypothetical protein
Mv_NIES-102_05323	Y	100%	100%	1334		hypothetical protein
Mv_NIES-102_05324	Y	100%	100%	341		hypothetical protein
Mv_NIES-102_05325	Y	100%	100%	566		hypothetical protein
Mv_NIES-102_05329	Y	100%	100%	2570		hypothetical protein
Mv_NIES-102_05396	Y	100%	100%	380		hypothetical protein

Representative gene (Mv_NIES-102 as reference)	Used for core genome tree?	% presence	% intact & single-copy	No. BP	Name	Product
Mv_NIES-102_05401	Y	100%	100%	566		hypothetical protein
Mv_NIES-102_05404	Y	100%	100%	161		hypothetical protein
Mv_NIES-102_05405	Y	100%	100%	164		hypothetical protein
Mv_NIES-102_05406	Y	100%	100%	455		hypothetical protein
Mv_NIES-102_05408	Y	100%	100%	1049		hypothetical protein
Mv_NIES-102_05414	Y	100%	100%	1181		hypothetical protein
Mv_NIES-102_05415	Y	100%	100%	266		hypothetical protein
Mv_NIES-102_05416	Y	100%	100%	239		hypothetical protein
Mv_NIES-102_05425	Y	100%	100%	377		hypothetical protein
Mv_NIES-102_05439	Y	100%	100%	515		hypothetical protein
Mv_NIES-102_05471	Y	100%	100%	407		hypothetical protein
Mv_NIES-102_05474	Y	100%	100%	821		hypothetical protein
Mv_NIES-102_05481	Y	100%	100%	284		hypothetical protein
Mv_NIES-102_05505	Y	100%	100%	1163		hypothetical protein
Mv_NIES-102_05507	Y	100%	100%	1043		hypothetical protein
Mv_NIES-102_05512	Y	100%	100%	1001		hypothetical protein
Mv_NIES-102_05521	Y	100%	100%	1064		hypothetical protein
Mv_NIES-102_05522	Y	100%	100%	857		hypothetical protein
Mv_NIES-102_05529	Y	100%	100%	323		hypothetical protein
Mv_NIES-102_05530	Y	100%	100%	611		hypothetical protein
Mv_NIES-102_05588	Y	100%	100%	620		hypothetical protein
Mv_NIES-102_05589	Y	100%	100%	1526		hypothetical protein
Mv_NIES-102_05590	Y	100%	100%	830		hypothetical protein
Mv_NIES-102_05592	Y	100%	100%	980		hypothetical protein
Mv_NIES-102_05613	Y	100%	100%	1829		hypothetical protein
Mv_NIES-102_05662	Y	100%	100%	1130		hypothetical protein
Mv_NIES-102_05664	Y	100%	100%	686		hypothetical protein
Mv_NIES-102_05674	Y	100%	100%	320		hypothetical protein
Mv_NIES-102_05682	Y	100%	100%	497		hypothetical protein
Mv_NIES-102_05686	Y	100%	100%	1964		hypothetical protein
Mv_NIES-102_05687	Y	100%	100%	641		hypothetical protein
Mv_NIES-102_05688	Y	100%	100%	446		hypothetical protein
Mv_NIES-102_05690	Y	100%	100%	683		hypothetical protein
Mv_NIES-102_05691	Y	100%	100%	599		hypothetical protein
Mv_NIES-102_05692	Y	100%	100%	818		hypothetical protein
Mv_NIES-102_05693	Y	100%	100%	806		hypothetical protein
Mv_NIES-102_05712	Y	100%	100%	191		hypothetical protein
Mv_NIES-102_05713	Y	100%	100%	1076		hypothetical protein
Mv_NIES-102_05729	Y	100%	100%	641		hypothetical protein
Mv_NIES-102_00031	Y	100%	100%	476		Inorganic triphosphatase
Mv_NIES-102_04064	Y	100%	100%	650		Iron-sulfur cluster carrier protein
Mv_NIES-102_05252	Y	100%	100%	944		Isoaspartyl peptidase/L-asparaginase
Mv_NIES-102_05506	Y	100%	100%	1043		L-Ala-D/L-Glu epimerase
Mv_NIES-102_03923	Y	100%	100%	170		Metallothionein
Mv_NIES-102_04424	Y	100%	100%	1391		NAD-dependent malic enzyme
Mv_NIES-102_00129	Y	100%	100%	587		Nucleoside triphosphate pyrophosphatase
Mv_NIES-102_05437	Y	100%	100%	962		Orange carotenoid-binding protein
Mv_NIES-102_00041	Y	100%	100%	500		Pentapeptide repeat protein Rfr32
Mv_NIES-102_03895	Y	100%	100%	509		Pentapeptide repeat protein Rfr32

Representative gene (Mv_NIES-102 as reference)	Used for core genome tree?	% presence	% intact & single-copy	No. BP	Name	Product
Mv_NIES-102_04960	Y	100%	100%	485		Pentapeptide repeat protein Rfr32
Mv_NIES-102_01204	Y	100%	100%	635		Peroxiredoxin
Mv_NIES-102_05112	Y	100%	100%	542		Peroxiredoxin
Mv_NIES-102_01853	Y	100%	100%	956		PhoH-like protein
Mv_NIES-102_00051	Y	100%	100%	401		Photosystem II lipoprotein Psb27
Mv_NIES-102_02820	Y	100%	100%	338		Photosystem II reaction center Psb28 protein
Mv_NIES-102_03612	Y	100%	100%	1493		Polyphosphate:AMP phosphotransferase
Mv_NIES-102_00002	Y	100%	100%	950		Protoheme IX farnesyltransferase
Mv_NIES-102_05032	Y	100%	100%	1814		putative ABC transporter ATP-binding protein
Mv_NIES-102_04187	Y	100%	100%	1742		putative ABC transporter-binding protein
Mv_NIES-102_05775	Y	100%	100%	1385		Putative cytochrome P450 135A1
Mv_NIES-102_05696	Y	100%	100%	860		putative ketoamine kinase
Mv_NIES-102_03696	Y	100%	100%	518		Putative low molecular weight protein-tyrosine-phosphatase
Mv_NIES-102_00710	Y	100%	100%	1727		Putative multidrug export ATP-binding/permease protein
Mv_NIES-102_05171	Y	100%	100%	953		putative oxidoreductase
Mv_NIES-102_00615	Y	100%	100%	887		putative protein
Mv_NIES-102_00959	Y	100%	100%	899		putative protein
Mv_NIES-102_03759	Y	100%	100%	323		putative protein
Mv_NIES-102_04261	Y	100%	100%	1859		putative protein
Mv_NIES-102_04626	Y	100%	100%	1562		putative protein
Mv_NIES-102_03286	Y	100%	100%	1337		putative transporter
Mv_NIES-102_05427	Y	100%	100%	965		Putative two-component membrane permease complex subunit SMU_747c
Mv_NIES-102_02338	Y	100%	100%	488		Putative universal stress protein
Mv_NIES-102_03937	Y	100%	100%	1556		putative zinc protease
Mv_NIES-102_05614	Y	100%	100%	1277		putative zinc protease
Mv_NIES-102_05699	Y	100%	100%	1211		putative zinc protease
Mv_NIES-102_02958	Y	100%	100%	659		Pyridoxal phosphate homeostasis protein
Mv_NIES-102_00110	Y	100%	100%	944		Renalase
Mv_NIES-102_01325	Y	100%	100%	980		S-adenosylmethionine/S-adenosylhomocysteine transporter
Mv_NIES-102_04328	Y	100%	100%	317		Thioredoxin-like protein
Mv_NIES-102_00100	Y	100%	100%	311		Ycf54-like protein

Table S3.

ANI and dDDH basis for genospecies determination. Number of monophyletic clusters in both core and pan gene phylogenies (see Fig. 2), number of genomes (strains) in each cluster, and the minimum within cluster and maximum between cluster ANI and dDDH values for each group. Minimum OGRI thresholds were chosen as the lowest value observed across all clusters with three or more genomes. Clusters with acceptable OGRI values (i.e., minimum within cluster ANI and dDDH values ≥ 0.970 and 0.750, respectively, and maximum between cluster values < 0.970 and 0.750, respectively) are indicated in **bold** and labeled as putative genospecies.

Cluster	No. of Genomes	ANI		dDDH		Genospecies
		Minimum within cluster	Maximum between cluster	Minimum within cluster	Maximum between cluster	
**1**	**2**	**0.983**	**0.956**	**0.865**	**0.652**	**A**
**2**	**4**	**0.990**	**0.969**	**0.929**	**0.668**	**B**
**3**	**4**	**0.998**	**0.958**	**0.990**	**0.659**	**C**
**4**	**3**	**0.986**	**0.969**	**0.881**	**0.740**	**D**
5	3	0.970	0.970	0.753	0.740	
**6**	**2**	**0.981**	**0.958**	**0.835**	**0.656**	**E**
7	2	0.963	0.963	0.703	0.702	
8	2	0.966	0.964	0.720	0.703	
**9**	**3**	**0.989**	**0.969**	**0.913**	**0.746**	**F**
**10**	**16**	**0.971**	**0.969**	**0.758**	**0.746**	**G**
**11**	**5**	**0.970**	**0.960**	**0.753**	**0.673**	**H**
**12**	**3**	**0.988**	**0.961**	**0.894**	**0.673**	**I**
**13**	**3**	**0.979**	**0.961**	**0.814**	**0.670**	**J**
**14**	**6**	**0.988**	**0.959**	**0.898**	**0.669**	**K**
**15**	**2**	**0.972**	**0.963**	**0.766**	**0.681**	**L**
**16**	**4**	**0.984**	**0.964**	**0.864**	**0.700**	**M**
**17**	**6**	**0.971**	**0.964**	**0.750**	**0.702**	**N**
18	3	0.983	0.971	0.859	0.753	
19	2	0.986	0.973	0.875	0.765	
20	2	0.974	0.973	0.771	0.769	
21	5	0.972	0.973	0.772	0.769	
**22**	**2**	**0.988**	**0.969**	**0.905**	**0.736**	**O**
**23**	**29**	**0.971**	**0.962**	**0.770**	**0.680**	**P**

Table S4.

Sequence similarity matrix of 16S rRNA genes extracted from *Microcystis* genomes (begin next page). Pairwise comparisons of sequence similarities for the 61 full length 16S sequences identified in the set of 122 genomes. Each sequence was compared to all others in the dataset using MUSCLE alignment tool built-in MEGA (*44*). The lowest pairwise sequence similarity across the entire set of 1,653 comparisons was 0.9931.

Genome ID	Ma_1130S32	Ma_BLCCF108	Ma_BLCCF158	Ma_FD4	Ma_KLA2	Ma_KWA	Ma_L211_11
Ma_1130S32							
Ma_BLCCF108	0.99662166						
Ma_BLCCF158	0.99593918	0.99797373					
Ma_FD4	0.99932553	0.99730003	0.99661937				
Ma_KLA2	0.9966174	0.99729743	0.99661611	0.99729663			
Ma_KWA	0.99662066	0.99458883	0.99390219	0.99729923	0.99458199		
Ma_L211_11	0.99729833	0.99526838	0.99458335	0.99797577	0.99526242	0.99932576	
Ma_LEGE11464	0.99389658	0.99458179	0.99389425	0.99458018	0.99457494	0.99593816	0.99525802
Ma_LEGE91341	0.99594448	0.99932596	0.9972973	0.99662378	0.99662053	0.99391017	0.99459043
Ma_LG11_05	0.9979745	0.99865141	0.99662037	0.99865101	0.99729743	0.99594463	0.99662278
Ma_MB_S_20031200_S102D	0.99661953	0.99729913	0.99661824	0.99729833	0.99729573	0.99458541	0.9952654
Ma_NIES_1211	0.99594448	0.99797637	0.99594294	0.99662378	0.99662053	0.99662178	0.99729923
Ma_NIES_2481	0.99662166	0.99730082	0.99662037	0.99730003	0.99729743	0.99729843	0.99797517
Ma_NIES_2519	0.99594448	0.99797637	0.99594294	0.99662378	0.99662053	0.99662178	0.99729923
Ma_NIES_2520	0.99797322	0.99594328	0.99525921	0.99865017	0.99593816	0.99729833	0.9979751
Ma_NIES_2521	0.99662166	1	0.99797373	0.99730003	0.99729743	0.99458883	0.99526838
Ma_NIES_2522	0.9952654	0.99730003	0.9952636	0.99594583	0.99594193	0.99594343	0.99662178
Ma_NIES_2549	0.99662166	0.99730082	0.99662037	0.99730003	0.99729743	0.99729843	0.99797517
Ma_NIES_298	0.9979745	0.99865141	0.99797373	0.99865101	0.99864972	0.99594463	0.99662278
Ma_NIES_3787	0.99594193	0.99662266	0.99594038	0.99662166	0.9966184	0.99661965	0.99729753
Ma_NIES_3804	0.99526382	0.99729913	0.99526202	0.99594448	0.99594057	0.99594208	0.99662066
Ma_NIES_3806	0.99662166	0.99730082	0.99662037	0.99730003	0.99729743	0.99729843	0.99797517
Ma_NIES_3807	0.99662166	0.99730082	0.99662037	0.99730003	0.99729743	0.99729843	0.99797517
Ma_NIES_4264	0.99662166	0.99730082	0.99662037	0.99730003	0.99729743	0.99729843	0.99797517
Ma_NIES_4285	0.99729663	0.9979751	0.9972956	0.9979745	0.99932553	0.995264	0.99594328
Ma_NIES_4325	0.99526101	0.99594328	0.99797654	0.99594208	0.9966174	0.9932227	0.99390451
Ma_NIES_44	0.99594448	0.99797637	0.9972973	0.99662378	0.99662053	0.99662178	0.99729923
Ma_NIES_843	0.99865057	0.99797577	0.99594174	0.99932596	0.99661953	0.99662278	0.99730003
Ma_NIES_87	0.99458179	0.99526523	0.99457973	0.99526382	0.99525925	0.99526101	0.99594072
Ma_NIES_88	0.99932553	0.99730003	0.99661937	1	0.99729663	0.99729923	0.99797577
Ma_PCC7005	0.99661853	0.99729833	0.99661724	0.99729753	0.99865017	0.99594328	0.995264
Ma_PCC7806SL	0.99729833	0.99932596	0.99864965	0.99797577	0.99797382	0.99526698	0.99594583
Ma_PCC7941	0.9979745	0.99865141	0.99797373	0.99865101	0.99864972	0.99594463	0.99662278
Ma_PCC9432	0.99729663	0.9979751	0.9972956	0.9979745	0.99797255	0.995264	0.99594328
Ma_PCC9443	0.995264	0.99865061	0.99661837	0.99594463	0.99594072	0.99322698	0.99390836
Ma_PCC9701	0.99729913	0.99662378	0.99594174	0.99797637	0.99661953	0.99797517	0.99865101
Ma_PCC9717	0.99662166	1	0.99797373	0.99730003	0.99729743	0.99458883	0.99526838
Ma_PCC9806	0.9952668	0.99730082	0.99526501	0.99594703	0.99594313	0.99594463	0.99662278
Ma_PCC9807	0.9979745	0.99865141	0.99662037	0.99865101	0.99729743	0.99594463	0.99662278
Ma_PCC9808	0.99729833	0.99932596	0.99864965	0.99797577	0.99797382	0.99526698	0.99594583
Ma_PCC9809	0.99729833	0.99526838	0.99458335	0.99797577	0.99526242	0.99932576	1
Ma_QC_Ch_20071001_M135	0.99797322	0.99594328	0.99525921	0.99865017	0.99593816	0.99729833	0.9979751
Ma_SC_T_19800800_S464	0.9979745	0.99865141	0.99797373	0.99865101	0.99864972	0.99594463	0.99662278
Ma_Sj	0.99729663	0.9979751	0.9972956	0.9979745	0.99932553	0.995264	0.99594328
Ma_TAIHU98	0.99594193	0.99662266	0.99594038	0.99662166	0.9966184	0.99661965	0.99729753
Mf_FACHB_1344	0.9979745	0.99729923	0.99661837	0.99865101	0.99729583	0.99730003	0.99797637
Mf_QC_C_20070823_S20D	0.99729913	0.99662378	0.99594174	0.99797637	0.99661953	0.99797517	0.99865101
Mf_WU_F_19750830_S460	0.9952668	0.99730082	0.99526501	0.99594703	0.99594313	0.99594463	0.99662278
Mn_MB_F_20050700_S1D	0.99594448	0.99932596	0.9972973	0.99662378	0.99662053	0.99391017	0.99459043
Mp_FACHB_1757	0.99729833	0.99932596	0.99864965	0.99797577	0.99797382	0.99526698	0.99594583
Mp_MB_F_20051200_S9D	0.99729663	0.9979751	0.9972956	0.9979745	0.99932553	0.995264	0.99594328
Msp_0824	0.99729913	0.99932576	0.9972965	0.99797637	0.99661953	0.99526838	0.99594703
Msp_LEGE00066	0.99729833	0.99797637	0.9972973	0.99797577	0.99662053	0.99662178	0.99729923
Msp_MC19	0.98768159	0.98975588	0.98767686	0.98837776	0.9883664	0.98768207	0.98837427
Msp_OC_L_20101000_S702	0.99729663	0.9979751	0.9972956	0.9979745	0.99932553	0.995264	0.99594328
Msp_T1_4	0.99594448	0.99797637	0.99594294	0.99662378	0.99662053	0.99662178	0.99729923
Mv_FACHB_1342	0.99729833	0.99797637	0.9972973	0.99797577	0.99662053	0.99662178	0.99729923
Mv_NIES_102	0.99729833	0.99526838	0.99458335	0.99797577	0.99526242	0.99932576	1
Mw_FACHB_1317	0.99729753	0.99797577	0.99594174	0.99797517	0.99661953	0.99662278	0.99594463
Mw_FACHB_1339	0.99594448	0.99797637	0.9972973	0.99662378	0.99662053	0.99662178	0.99729923
Mw_MB_S_20031200_S109D	0.99458702	0.99797577	0.99594174	0.99526838	0.99526382	0.99526558	0.99594463

Genome ID	Ma_LEGE11464	Ma_LEGE91341	Ma_LG11_05	Ma_MB_S_20031200_S102D	Ma_NIES_1211	Ma_NIES_2481	Ma_NIES_2519
Ma_1130S32							
Ma_BLCCF108							
Ma_BLCCF158							
Ma_FD4							
Ma_KLA2							
Ma_KWA							
Ma_L211_11							
Ma_LEGE11464							
Ma_LEGE91341	0.99390225						
Ma_LG11_05	0.99458179	0.99797637					
Ma_MB_S_20031200_S102D	0.99457837	0.99662266	0.99729913				
Ma_NIES_1211	0.9966174	0.99730082	0.99797637	0.99662266			
Ma_NIES_2481	0.99729493	0.99662478	0.99730082	0.99729913	0.99932596		
Ma_NIES_2519	0.9966174	0.99730082	0.99797637	0.99662266	1	0.99932596	
Ma_NIES_2520	0.99390247	0.9952654	0.99729753	0.99594072	0.9952654	0.99594328	0.9952654
Ma_NIES_2521	0.99458179	0.99932596	0.99865141	0.99729913	0.99797637	0.99730082	0.99797637
Ma_NIES_2522	0.99593816	0.99662378	0.99730003	0.99594448	0.99932576	0.99865101	0.99932576
Ma_NIES_2549	0.99729493	0.99662478	0.99730082	0.99729913	0.99932596	1	0.99932596
Ma_NIES_298	0.99593937	0.99797637	0.99865141	0.99865057	0.99797637	0.99865141	0.99797637
Ma_NIES_3787	0.99797322	0.99594568	0.99662266	0.99662053	0.99865057	0.99932553	0.99865057
Ma_NIES_3804	0.99729663	0.99662266	0.99729913	0.99594313	0.99932553	0.99865057	0.99932553
Ma_NIES_3806	0.99729493	0.99662478	0.99730082	0.99729913	0.99932596	1	0.99932596
Ma_NIES_3807	0.99729493	0.99662478	0.99730082	0.99729913	0.99932596	1	0.99932596
Ma_NIES_4264	0.99729493	0.99662478	0.99730082	0.99729913	0.99932596	1	0.99932596
Ma_NIES_4285	0.99525784	0.99729913	0.9979751	0.99797382	0.99729913	0.9979751	0.99729913
Ma_NIES_4325	0.99321387	0.9952654	0.99594328	0.99594072	0.9952654	0.99594328	0.9952654
Ma_NIES_44	0.9966174	0.99730082	0.99662478	0.99662266	0.99865141	0.99932596	0.99865141
Ma_NIES_843	0.99390043	0.99730003	0.99932576	0.99662166	0.99730003	0.99662378	0.99730003
Ma_NIES_87	0.9979745	0.99458682	0.99526523	0.99526224	0.99729743	0.99797382	0.99729743
Ma_NIES_88	0.99458018	0.99662378	0.99865101	0.99729833	0.99662378	0.99730003	0.99662378
Ma_PCC7005	0.99593801	0.99662166	0.99729833	0.99729663	0.99662166	0.99729833	0.99662166
Ma_PCC7806SL	0.99526083	0.99865141	0.99797637	0.9979751	0.99730082	0.99797637	0.99730082
Ma_PCC7941	0.99593937	0.99797637	0.99865141	0.99865057	0.99797637	0.99865141	0.99797637
Ma_PCC9432	0.99661953	0.99729913	0.9979751	0.99797382	0.99729913	0.9979751	0.99729913
Ma_PCC9443	0.99390043	0.99797517	0.99729923	0.99594328	0.99662278	0.99594583	0.99662278
Ma_PCC9701	0.9966164	0.99594703	0.99662378	0.99662166	0.99865101	0.99932576	0.99865101
Ma_PCC9717	0.99458179	0.99932596	0.99865141	0.99729913	0.99797637	0.99730082	0.99797637
Ma_PCC9806	0.99593937	0.99662478	0.99730082	0.99594568	0.99932596	0.99865141	0.99932596
Ma_PCC9807	0.99458179	0.99797637	1	0.99729913	0.99797637	0.99730082	0.99797637
Ma_PCC9808	0.99526083	0.99865141	0.99797637	0.9979751	0.99730082	0.99797637	0.99730082
Ma_PCC9809	0.99525802	0.99459043	0.99662278	0.9952654	0.99729923	0.99797517	0.99729923
Ma_QC_Ch_20071001_M135	0.99458199	0.9952654	0.99729753	0.99594072	0.9952654	0.99594328	0.9952654
Ma_SC_T_19800800_S464	0.99593937	0.99797637	0.99865141	0.99865057	0.99797637	0.99865141	0.99797637
Ma_Sj	0.99525784	0.99729913	0.9979751	0.99797382	0.99729913	0.9979751	0.99729913
Ma_TAIHU98	0.99797322	0.99594568	0.99662266	0.99662053	0.99865057	0.99932553	0.99865057
Mf_FACHB_1344	0.99457857	0.99662278	0.99729923	0.99729753	0.99662278	0.99729923	0.99662278
Mf_QC_C_20070823_S20D	0.9966164	0.99594703	0.99662378	0.99662166	0.99865101	0.99932576	0.99865101
Mf_WU_F_19750830_S460	0.99593937	0.99662478	0.99730082	0.99594568	0.99932596	0.99865141	0.99932596
Mn_MB_F_20050700_S1D	0.99390225	0.99865141	0.99797637	0.99662266	0.99730082	0.99662478	0.99730082
Mp_FACHB_1757	0.99526083	0.99865141	0.99797637	0.9979751	0.99730082	0.99797637	0.99730082
Mp_MB_F_20051200_S9D	0.99525784	0.99729913	0.9979751	0.99797382	0.99729913	0.9979751	0.99729913
Msp_0824	0.99390043	0.99865101	0.99797577	0.99662166	0.99730003	0.99662378	0.99730003
Msp_LEGE00066	0.99526083	0.99730082	0.99797637	0.99662266	0.99730082	0.99797637	0.99730082
Msp_MC19	0.98766582	0.98906882	0.98975588	0.98837383	0.99112844	0.99044242	0.99112844
Msp_OC_L_20101000_S702	0.99525784	0.99729913	0.9979751	0.99797382	0.99729913	0.9979751	0.99729913
Msp_T1_4	0.9966174	0.99730082	0.99797637	0.99662266	1	0.99932596	1
Mv_FACHB_1342	0.99526083	0.99730082	0.99797637	0.99662266	0.99730082	0.99797637	0.99730082
Mv_NIES_102	0.99525802	0.99459043	0.99662278	0.9952654	0.99729923	0.99797517	0.99729923
Mw_FACHB_1317	0.99526224	0.99730003	0.99932576	0.99662166	0.99730003	0.99662378	0.99730003
Mw_FACHB_1339	0.9966174	0.99730082	0.99662478	0.99662266	0.99865141	0.99932596	0.99865141
Mw_MB_S_20031200_S109D	0.99525943	0.99730003	0.99662378	0.9952668	0.99865101	0.99797577	0.99865101

Genome ID	Ma_NIES_2520	Ma_NIES_2521	Ma_NIES_2522	Ma_NIES_2549	Ma_NIES_298	Ma_NIES_3787	Ma_NIES_3804
Ma_1130S32							
Ma_BLCCF108							
Ma_BLCCF158							
Ma_FD4							
Ma_KLA2							
Ma_KWA							
Ma_L211_11							
Ma_LEGE11464							
Ma_LEGE91341							
Ma_LG11_05							
Ma_MB_S_20031200_S102D							
Ma_NIES_1211							
Ma_NIES_2481							
Ma_NIES_2519							
Ma_NIES_2520							
Ma_NIES_2521	0.99594328						
Ma_NIES_2522	0.99458541	0.99730003					
Ma_NIES_2549	0.99594328	0.99730082	0.99865101				
Ma_NIES_298	0.99729753	0.99865141	0.99730003	0.99865141			
Ma_NIES_3787	0.99594583	0.99662266	0.9979745	0.99932553	0.9979751		
Ma_NIES_3804	0.9945836	0.99729913	0.99865017	0.99865057	0.99729913	0.99797382	
Ma_NIES_3806	0.99594328	0.99730082	0.99865101	1	0.99865141	0.99932553	0.99865057
Ma_NIES_3807	0.99594328	0.99730082	0.99865101	1	0.99865141	0.99932553	0.99865057
Ma_NIES_4264	0.99594328	0.99730082	0.99865101	1	0.99865141	0.99932553	0.99865057
Ma_NIES_4285	0.99661853	0.9979751	0.99662166	0.9979751	0.99932553	0.99729743	0.99662053
Ma_NIES_4325	0.99458038	0.99594328	0.99458541	0.99594328	0.99729753	0.99526242	0.9945836
Ma_NIES_44	0.9952654	0.99797637	0.99797577	0.99932596	0.99797637	0.99865057	0.9979751
Ma_NIES_843	0.9979745	0.99797577	0.99662278	0.99662378	0.99797577	0.99594448	0.99662166
Ma_NIES_87	0.99458702	0.99526523	0.99661953	0.99797382	0.99662053	0.99865057	0.9979751
Ma_NIES_88	0.99865017	0.99730003	0.99594583	0.99730003	0.99865101	0.99662166	0.99594448
Ma_PCC7005	0.99593952	0.99729833	0.99594328	0.99729833	0.99865017	0.99661953	0.99594193
Ma_PCC7806SL	0.99662066	0.99932596	0.99662378	0.99797637	0.99932596	0.99729913	0.99662266
Ma_PCC7941	0.99729753	0.99865141	0.99730003	0.99865141	1	0.9979751	0.99729913
Ma_PCC9432	0.99729923	0.9979751	0.99662166	0.9979751	0.99932553	0.99865141	0.99662053
Ma_PCC9443	0.995264	0.99865061	0.99594463	0.99594583	0.99729923	0.99594448	0.99594328
Ma_PCC9701	0.99662166	0.99662378	0.99797517	0.99932576	0.99797577	0.99865017	0.9979745
Ma_PCC9717	0.99594328	1	0.99730003	0.99730082	0.99865141	0.99662266	0.99729913
Ma_PCC9806	0.99458702	0.99730082	0.99865101	0.99865141	0.99730082	0.9979751	0.99865057
Ma_PCC9807	0.99729753	0.99865141	0.99730003	0.99730082	0.99865141	0.99662266	0.99729913
Ma_PCC9808	0.99662066	0.99932596	0.99662378	0.99797637	0.99932596	0.99729913	0.99662266
Ma_PCC9809	0.9979751	0.99526838	0.99662178	0.99797517	0.99662278	0.99729753	0.99662066
Ma_QC_Ch_20071001_M135	0.99932596	0.99594328	0.99458541	0.99594328	0.99729753	0.99662278	0.9945836
Ma_SC_T_19800800_S464	0.99729753	0.99865141	0.99730003	0.99865141	1	0.9979751	0.99729913
Ma_Sj	0.99661853	0.9979751	0.99662166	0.9979751	0.99932553	0.99729743	0.99662053
Ma_TAIHU98	0.99594583	0.99662266	0.9979745	0.99932553	0.9979751	1	0.99797382
Mf_FACHB_1344	0.99865057	0.99729923	0.99594463	0.99729923	0.99865061	0.99662066	0.99594328
Mf_QC_C_20070823_S20D	0.99662166	0.99662378	0.99797517	0.99932576	0.99797577	0.99865017	0.9979745
Mf_WU_F_19750830_S460	0.99458702	0.99730082	0.99865101	0.99865141	0.99730082	0.9979751	0.99865057
Mn_MB_F_20050700_S1D	0.9952654	0.99932596	0.99662378	0.99662478	0.99797637	0.99594568	0.99662266
Mp_FACHB_1757	0.99662066	0.99932596	0.99662378	0.99797637	0.99932596	0.99729913	0.99662266
Mp_MB_F_20051200_S9D	0.99661853	0.9979751	0.99662166	0.9979751	0.99932553	0.99729743	0.99662053
Msp_0824	0.99662166	0.99932576	0.99662278	0.99662378	0.99797577	0.99594448	0.99662166
Msp_LEGE00066	0.99662066	0.99797637	0.99662378	0.99797637	0.99797637	0.99729913	0.99662266
Msp_MC19	0.98698842	0.98975588	0.99043956	0.99044242	0.98975588	0.98974934	0.99043632
Msp_OC_L_20101000_S702	0.99661853	0.9979751	0.99662166	0.9979751	0.99932553	0.99729743	0.99662053
Msp_T1_4	0.9952654	0.99797637	0.99932576	0.99932596	0.99797637	0.99865057	0.99932553
Mv_FACHB_1342	0.99662066	0.99797637	0.99662378	0.99797637	0.99797637	0.99729913	0.99662266
Mv_NIES_102	0.9979751	0.99526838	0.99662178	0.99797517	0.99662278	0.99729753	0.99662066
Mw_FACHB_1317	0.99661965	0.99797577	0.99662278	0.99662378	0.99797577	0.99594448	0.99662166
Mw_FACHB_1339	0.9952654	0.99797637	0.99797577	0.99932596	0.99797637	0.99865057	0.9979751
Mw_MB_S_20031200_S109D	0.99390632	0.99797577	0.99797517	0.99797577	0.99662378	0.99729833	0.9979745

Genome ID	Ma_NIES_3806	Ma_NIES_3807	Ma_NIES_4264	Ma_NIES_4285	Ma_NIES_4325	Ma_NIES_44	Ma_NIES_843
Ma_1130S32							
Ma_BLCCF108							
Ma_BLCCF158							
Ma_FD4							
Ma_KLA2							
Ma_KWA							
Ma_L211_11							
Ma_LEGE11464							
Ma_LEGE91341							
Ma_LG11_05							
Ma_MB_S_20031200_S102D							
Ma_NIES_1211							
Ma_NIES_2481							
Ma_NIES_2519							
Ma_NIES_2520							
Ma_NIES_2521							
Ma_NIES_2522							
Ma_NIES_2549							
Ma_NIES_298							
Ma_NIES_3787							
Ma_NIES_3804							
Ma_NIES_3806							
Ma_NIES_3807	1						
Ma_NIES_4264	1	1					
Ma_NIES_4285	0.9979751	0.9979751	0.9979751				
Ma_NIES_4325	0.99594328	0.99594328	0.99594328	0.99729663			
Ma_NIES_44	0.99932596	0.99932596	0.99932596	0.99729913	0.9952654		
Ma_NIES_843	0.99662378	0.99662378	0.99662378	0.99729833	0.995264	0.99594703	
Ma_NIES_87	0.99797382	0.99797382	0.99797382	0.99594057	0.99390043	0.99729743	0.99458521
Ma_NIES_88	0.99730003	0.99730003	0.99730003	0.9979745	0.99594208	0.99662378	0.99932596
Ma_PCC7005	0.99729833	0.99729833	0.99729833	0.99932576	0.99661853	0.99662166	0.99662066
Ma_PCC7806SL	0.99797637	0.99797637	0.99797637	0.99865057	0.99662066	0.99865141	0.99730003
Ma_PCC7941	0.99865141	0.99865141	0.99865141	0.99932553	0.99729753	0.99797637	0.99797577
Ma_PCC9432	0.9979751	0.9979751	0.9979751	0.99864972	0.99661853	0.99729913	0.99729833
Ma_PCC9443	0.99594583	0.99594583	0.99594583	0.99662066	0.9945838	0.99662278	0.99662178
Ma_PCC9701	0.99932576	0.99932576	0.99932576	0.99729833	0.995264	0.99865101	0.99730082
Ma_PCC9717	0.99730082	0.99730082	0.99730082	0.9979751	0.99594328	0.99797637	0.99797577
Ma_PCC9806	0.99865141	0.99865141	0.99865141	0.99662266	0.99458702	0.99797637	0.99662378
Ma_PCC9807	0.99730082	0.99730082	0.99730082	0.9979751	0.99594328	0.99662478	0.99932576
Ma_PCC9808	0.99797637	0.99797637	0.99797637	0.99865057	0.99662066	0.99865141	0.99730003
Ma_PCC9809	0.99797517	0.99797517	0.99797517	0.99594328	0.99390451	0.99729923	0.99730003
Ma_QC_Ch_20071001_M135	0.99594328	0.99594328	0.99594328	0.99661853	0.99458038	0.9952654	0.9979745
Ma_SC_T_19800800_S464	0.99865141	0.99865141	0.99865141	0.99932553	0.99729753	0.99797637	0.99797577
Ma_Sj	0.9979751	0.9979751	0.9979751	1	0.99729663	0.99729913	0.99729833
Ma_TAIHU98	0.99932553	0.99932553	0.99932553	0.99729743	0.99526242	0.99865057	0.99594448
Mf_FACHB_1344	0.99729923	0.99729923	0.99729923	0.9979739	0.99594087	0.99662278	0.99797577
Mf_QC_C_20070823_S20D	0.99932576	0.99932576	0.99932576	0.99729833	0.995264	0.99865101	0.99730082
Mf_WU_F_19750830_S460	0.99865141	0.99865141	0.99865141	0.99662266	0.99458702	0.99797637	0.99662378
Mn_MB_F_20050700_S1D	0.99662478	0.99662478	0.99662478	0.99729913	0.9952654	0.99730082	0.99730003
Mp_FACHB_1757	0.99797637	0.99797637	0.99797637	0.99865057	0.99662066	0.99865141	0.99730003
Mp_MB_F_20051200_S9D	0.9979751	0.9979751	0.9979751	1	0.99729663	0.99729913	0.99729833
Msp_0824	0.99662378	0.99662378	0.99662378	0.99729833	0.995264	0.99730003	0.99865141
Msp_LEGE00066	0.99797637	0.99797637	0.99797637	0.99729913	0.9952654	0.99865141	0.99730003
Msp_MC19	0.99044242	0.99044242	0.99044242	0.98906184	0.98698842	0.98975588	0.98906554
Msp_OC_L_20101000_S702	0.9979751	0.9979751	0.9979751	1	0.99729663	0.99729913	0.99729833
Msp_T1_4	0.99932596	0.99932596	0.99932596	0.99729913	0.9952654	0.99865141	0.99730003
Mv_FACHB_1342	0.99797637	0.99797637	0.99797637	0.99729913	0.9952654	0.99865141	0.99730003
Mv_NIES_102	0.99797517	0.99797517	0.99797517	0.99594328	0.99390451	0.99729923	0.99730003
Mw_FACHB_1317	0.99662378	0.99662378	0.99662378	0.99729833	0.995264	0.99594703	0.99865061
Mw_FACHB_1339	0.99932596	0.99932596	0.99932596	0.99729913	0.9952654	1	0.99594703
Mw_MB_S_20031200_S109D	0.99797577	0.99797577	0.99797577	0.99594448	0.9952668	0.99865101	0.99594583

Genome ID	Ma_NIES_87	Ma_NIES_88	Ma_PCC7005	Ma_PCC7806SL	Ma_PCC7941	Ma_PCC9432	Ma_PCC9443
Ma_1130S32							
Ma_BLCCF108							
Ma_BLCCF158							
Ma_FD4							
Ma_KLA2							
Ma_KWA							
Ma_L211_11							
Ma_LEGE11464							
Ma_LEGE91341							
Ma_LG11_05							
Ma_MB_S_20031200_S102D							
Ma_NIES_1211							
Ma_NIES_2481							
Ma_NIES_2519							
Ma_NIES_2520							
Ma_NIES_2521							
Ma_NIES_2522							
Ma_NIES_2549							
Ma_NIES_298							
Ma_NIES_3787							
Ma_NIES_3804							
Ma_NIES_3806							
Ma_NIES_3807							
Ma_NIES_4264							
Ma_NIES_4285							
Ma_NIES_4325							
Ma_NIES_44							
Ma_NIES_843							
Ma_NIES_87							
Ma_NIES_88	0.99526382						
Ma_PCC7005	0.99526083	0.99729753					
Ma_PCC7806SL	0.99594313	0.99797577	0.9979745				
Ma_PCC7941	0.99662053	0.99865101	0.99865017	0.99932596			
Ma_PCC9432	0.99729913	0.9979745	0.99797322	0.99865057	0.99932553		
Ma_PCC9443	0.99458521	0.99594463	0.99594208	0.99797517	0.99729923	0.99729833	
Ma_PCC9701	0.99729663	0.99797637	0.99662066	0.99730003	0.99797577	0.99729833	0.99526698
Ma_PCC9717	0.99526523	0.99730003	0.99729833	0.99932596	0.99865141	0.9979751	0.99865061
Ma_PCC9806	0.99662053	0.99594703	0.99594448	0.99662478	0.99730082	0.99662266	0.99594583
Ma_PCC9807	0.99526523	0.99865101	0.99729833	0.99797637	0.99865141	0.9979751	0.99729923
Ma_PCC9808	0.99594313	0.99797577	0.9979745	1	0.99932596	0.99865057	0.99797517
Ma_PCC9809	0.99594072	0.99797577	0.995264	0.99594583	0.99662278	0.99594328	0.99390836
Ma_QC_Ch_20071001_M135	0.9952654	0.99865017	0.99593952	0.99662066	0.99729753	0.99797517	0.995264
Ma_SC_T_19800800_S464	0.99662053	0.99865101	0.99865017	0.99932596	1	0.99932553	0.99729923
Ma_Sj	0.99594057	0.9979745	0.99932576	0.99865057	0.99932553	0.99864972	0.99662066
Ma_TAIHU98	0.99865057	0.99662166	0.99661953	0.99729913	0.9979751	0.99865141	0.99594448
Mf_FACHB_1344	0.99526242	0.99865101	0.99729673	0.99797517	0.99865061	0.9979739	0.99594343
Mf_QC_C_20070823_S20D	0.99729663	0.99797637	0.99662066	0.99730003	0.99797577	0.99729833	0.99526698
Mf_WU_F_19750830_S460	0.99662053	0.99594703	0.99594448	0.99662478	0.99730082	0.99662266	0.99594583
Mn_MB_F_20050700_S1D	0.99458682	0.99662378	0.99662166	0.99865141	0.99797637	0.99729913	0.99797517
Mp_FACHB_1757	0.99594313	0.99797577	0.9979745	1	0.99932596	0.99865057	0.99797517
Mp_MB_F_20051200_S9D	0.99594057	0.9979745	0.99932576	0.99865057	0.99932553	0.99864972	0.99662066
Msp_0824	0.99458521	0.99797637	0.99662066	0.99865101	0.99797577	0.99729833	0.99797457
Msp_LEGE00066	0.99594313	0.99797577	0.99662166	0.99865141	0.99797637	0.99729913	0.99662278
Msp_MC19	0.9883664	0.98837776	0.98837034	0.98906882	0.98975588	0.98906184	0.98837427
Msp_OC_L_20101000_S702	0.99594057	0.9979745	0.99932576	0.99865057	0.99932553	0.99864972	0.99662066
Msp_T1_4	0.99729743	0.99662378	0.99662166	0.99730082	0.99797637	0.99729913	0.99662278
Mv_FACHB_1342	0.99594313	0.99797577	0.99662166	0.99865141	0.99797637	0.99729913	0.99662278
Mv_NIES_102	0.99594072	0.99797577	0.995264	0.99594583	0.99662278	0.99594328	0.99390836
Mw_FACHB_1317	0.99458521	0.99797517	0.9979751	0.99730003	0.99797577	0.99729833	0.99662178
Mw_FACHB_1339	0.99729743	0.99662378	0.99662166	0.99865141	0.99797637	0.99729913	0.99662278
Mw_MB_S_20031200_S109D	0.99594193	0.99526838	0.9952654	0.99730003	0.99662378	0.99594448	0.99662178

Genome ID	Ma_PCC9701	Ma_PCC9717	Ma_PCC9806	Ma_PCC9807	Ma_PCC9808	Ma_PCC9809	Ma_QC_Ch_20071001_M135
Ma_1130S32							
Ma_BLCCF108							
Ma_BLCCF158							
Ma_FD4							
Ma_KLA2							
Ma_KWA							
Ma_L211_11							
Ma_LEGE11464							
Ma_LEGE91341							
Ma_LG11_05							
Ma_MB_S_20031200_S102D							
Ma_NIES_1211							
Ma_NIES_2481							
Ma_NIES_2519							
Ma_NIES_2520							
Ma_NIES_2521							
Ma_NIES_2522							
Ma_NIES_2549							
Ma_NIES_298							
Ma_NIES_3787							
Ma_NIES_3804							
Ma_NIES_3806							
Ma_NIES_3807							
Ma_NIES_4264							
Ma_NIES_4285							
Ma_NIES_4325							
Ma_NIES_44							
Ma_NIES_843							
Ma_NIES_87							
Ma_NIES_88							
Ma_PCC7005							
Ma_PCC7806SL							
Ma_PCC7941							
Ma_PCC9432							
Ma_PCC9443							
Ma_PCC9701							
Ma_PCC9717	0.99662378						
Ma_PCC9806	0.99797577	0.99730082					
Ma_PCC9807	0.99662378	0.99865141	0.99730082				
Ma_PCC9808	0.99730003	0.99932596	0.99662478	0.99797637			
Ma_PCC9809	0.99865101	0.99526838	0.99662278	0.99662278	0.99594583		
Ma_QC_Ch_20071001_M135	0.99662166	0.99594328	0.99458702	0.99729753	0.99662066	0.9979751	
Ma_SC_T_19800800_S464	0.99797577	0.99865141	0.99730082	0.99865141	0.99932596	0.99662278	0.99729753
Ma_Sj	0.99729833	0.9979751	0.99662266	0.9979751	0.99865057	0.99594328	0.99661853
Ma_TAIHU98	0.99865017	0.99662266	0.9979751	0.99662266	0.99729913	0.99729753	0.99662278
Mf_FACHB_1344	0.99797577	0.99729923	0.99594583	0.99729923	0.99797517	0.99797637	0.99865057
Mf_QC_C_20070823_S20D	1	0.99662378	0.99797577	0.99662378	0.99730003	0.99865101	0.99662166
Mf_WU_F_19750830_S460	0.99797577	0.99730082	1	0.99730082	0.99662478	0.99662278	0.99458702
Mn_MB_F_20050700_S1D	0.99594703	0.99932596	0.99662478	0.99797637	0.99865141	0.99459043	0.9952654
Mp_FACHB_1757	0.99730003	0.99932596	0.99662478	0.99797637	1	0.99594583	0.99662066
Mp_MB_F_20051200_S9D	0.99729833	0.9979751	0.99662266	0.9979751	0.99865057	0.99594328	0.99661853
Msp_0824	0.99730082	0.99932576	0.99662378	0.99797577	0.99865101	0.99594703	0.99662166
Msp_LEGE00066	0.99730003	0.99797637	0.99662478	0.99797637	0.99865141	0.99729923	0.99662066
Msp_MC19	0.98975281	0.98975588	0.99044242	0.98975588	0.98906882	0.98837427	0.98698842
Msp_OC_L_20101000_S702	0.99729833	0.9979751	0.99662266	0.9979751	0.99865057	0.99594328	0.99661853
Msp_T1_4	0.99865101	0.99797637	0.99932596	0.99797637	0.99730082	0.99729923	0.9952654
Mv_FACHB_1342	0.99730003	0.99797637	0.99662478	0.99797637	0.99865141	0.99729923	0.99662066
Mv_NIES_102	0.99865101	0.99526838	0.99662278	0.99662278	0.99594583	1	0.9979751
Mw_FACHB_1317	0.99594583	0.99797577	0.99662378	0.99932576	0.99730003	0.99594463	0.99661965
Mw_FACHB_1339	0.99865101	0.99797637	0.99797637	0.99662478	0.99865141	0.99729923	0.9952654
Mw_MB_S_20031200_S109D	0.99729923	0.99797577	0.99797577	0.99662378	0.99730003	0.99594463	0.99390632

Genome ID	Ma_SC_T_19800800_S464	Ma_Sj	Ma_TAIHU98	Mf_FACHB_1344	Mf_QC_C_20070823_S20D	Mf_WU_F_19750830_S460	Mn_MB_F_20050700_S1D
Ma_1130S32							
Ma_BLCCF108							
Ma_BLCCF158							
Ma_FD4							
Ma_KLA2							
Ma_KWA							
Ma_L211_11							
Ma_LEGE11464							
Ma_LEGE91341							
Ma_LG11_05							
Ma_MB_S_20031200_S102D							
Ma_NIES_1211							
Ma_NIES_2481							
Ma_NIES_2519							
Ma_NIES_2520							
Ma_NIES_2521							
Ma_NIES_2522							
Ma_NIES_2549							
Ma_NIES_298							
Ma_NIES_3787							
Ma_NIES_3804							
Ma_NIES_3806							
Ma_NIES_3807							
Ma_NIES_4264							
Ma_NIES_4285							
Ma_NIES_4325							
Ma_NIES_44							
Ma_NIES_843							
Ma_NIES_87							
Ma_NIES_88							
Ma_PCC7005							
Ma_PCC7806SL							
Ma_PCC7941							
Ma_PCC9432							
Ma_PCC9443							
Ma_PCC9701							
Ma_PCC9717							
Ma_PCC9806							
Ma_PCC9807							
Ma_PCC9808							
Ma_PCC9809							
Ma_QC_Ch_20071001_M135							
Ma_SC_T_19800800_S464							
Ma_Sj	0.99932553						
Ma_TAIHU98	0.9979751	0.99729743					
Mf_FACHB_1344	0.99865061	0.9979739	0.99662066				
Mf_QC_C_20070823_S20D	0.99797577	0.99729833	0.99865017	0.99797577			
Mf_WU_F_19750830_S460	0.99730082	0.99662266	0.9979751	0.99594583	0.99797577		
Mn_MB_F_20050700_S1D	0.99797637	0.99729913	0.99594568	0.99662278	0.99594703	0.99662478	
Mp_FACHB_1757	0.99932596	0.99865057	0.99729913	0.99797517	0.99730003	0.99662478	0.99865141
Mp_MB_F_20051200_S9D	0.99932553	1	0.99729743	0.9979739	0.99729833	0.99662266	0.99729913
Msp_0824	0.99797577	0.99729833	0.99594448	0.99797577	0.99730082	0.99662378	0.99865101
Msp_LEGE00066	0.99797637	0.99729913	0.99729913	0.99662278	0.99730003	0.99662478	0.99730082
Msp_MC19	0.98975588	0.98906184	0.98974934	0.98837427	0.98975281	0.99044242	0.98906882
Msp_OC_L_20101000_S702	0.99932553	1	0.99729743	0.9979739	0.99729833	0.99662266	0.99729913
Msp_T1_4	0.99797637	0.99729913	0.99865057	0.99662278	0.99865101	0.99932596	0.99730082
Mv_FACHB_1342	0.99797637	0.99729913	0.99729913	0.99662278	0.99730003	0.99662478	0.99730082
Mv_NIES_102	0.99662278	0.99594328	0.99729753	0.99797637	0.99865101	0.99662278	0.99459043
Mw_FACHB_1317	0.99797577	0.99729833	0.99594448	0.99662178	0.99594583	0.99662378	0.99730003
Mw_FACHB_1339	0.99797637	0.99729913	0.99865057	0.99662278	0.99865101	0.99797637	0.99730082
Mw_MB_S_20031200_S109D	0.99662378	0.99594448	0.99729833	0.99526698	0.99729923	0.99797577	0.99730003

Genome ID	Mp_FACHB_1757	Mp_MB_F_20051200_S9D	Msp_0824	Msp_LEGE00066	Msp_MC19	Msp_OC_L_20101000_S702	Msp_T1_4
Ma_1130S32							
Ma_BLCCF108							
Ma_BLCCF158							
Ma_FD4							
Ma_KLA2							
Ma_KWA							
Ma_L211_11							
Ma_LEGE11464							
Ma_LEGE91341							
Ma_LG11_05							
Ma_MB_S_20031200_S102D							
Ma_NIES_1211							
Ma_NIES_2481							
Ma_NIES_2519							
Ma_NIES_2520							
Ma_NIES_2521							
Ma_NIES_2522							
Ma_NIES_2549							
Ma_NIES_298							
Ma_NIES_3787							
Ma_NIES_3804							
Ma_NIES_3806							
Ma_NIES_3807							
Ma_NIES_4264							
Ma_NIES_4285							
Ma_NIES_4325							
Ma_NIES_44							
Ma_NIES_843							
Ma_NIES_87							
Ma_NIES_88							
Ma_PCC7005							
Ma_PCC7806SL							
Ma_PCC7941							
Ma_PCC9432							
Ma_PCC9443							
Ma_PCC9701							
Ma_PCC9717							
Ma_PCC9806							
Ma_PCC9807							
Ma_PCC9808							
Ma_PCC9809							
Ma_QC_Ch_20071001_M135							
Ma_SC_T_19800800_S464							
Ma_Sj							
Ma_TAIHU98							
Mf_FACHB_1344							
Mf_QC_C_20070823_S20D							
Mf_WU_F_19750830_S460							
Mn_MB_F_20050700_S1D							
Mp_FACHB_1757							
Mp_MB_F_20051200_S9D	0.99865057						
Msp_0824	0.99865101	0.99729833					
Msp_LEGE00066	0.99865141	0.99729913	0.99730003				
Msp_MC19	0.98906882	0.98906184	0.98906554	0.98906882			
Msp_OC_L_20101000_S702	0.99865057	1	0.99729833	0.99729913	0.98906184		
Msp_T1_4	0.99730082	0.99729913	0.99730003	0.99730082	0.99112844	0.99729913	
Mv_FACHB_1342	0.99865141	0.99729913	0.99730003	1	0.98906882	0.99729913	0.99730082
Mv_NIES_102	0.99594583	0.99594328	0.99594703	0.99729923	0.98837427	0.99594328	0.99729923
Mw_FACHB_1317	0.99730003	0.99729833	0.99729923	0.99730003	0.98906554	0.99729833	0.99730003
Mw_FACHB_1339	0.99865141	0.99729913	0.99730003	0.99865141	0.98975588	0.99729913	0.99865141
Mw_MB_S_20031200_S109D	0.99730003	0.99594448	0.99729923	0.99730003	0.98975281	0.99594448	0.99865101

Genome ID	Mv_FACHB_1342	Mv_NIES_102	Mw_FACHB_1317	Mw_FACHB_1339
Ma_1130S32				
Ma_BLCCF108				
Ma_BLCCF158				
Ma_FD4				
Ma_KLA2				
Ma_KWA				
Ma_L211_11				
Ma_LEGE11464				
Ma_LEGE91341				
Ma_LG11_05				
Ma_MB_S_20031200_S102D				
Ma_NIES_1211				
Ma_NIES_2481				
Ma_NIES_2519				
Ma_NIES_2520				
Ma_NIES_2521				
Ma_NIES_2522				
Ma_NIES_2549				
Ma_NIES_298				
Ma_NIES_3787				
Ma_NIES_3804				
Ma_NIES_3806				
Ma_NIES_3807				
Ma_NIES_4264				
Ma_NIES_4285				
Ma_NIES_4325				
Ma_NIES_44				
Ma_NIES_843				
Ma_NIES_87				
Ma_NIES_88				
Ma_PCC7005				
Ma_PCC7806SL				
Ma_PCC7941				
Ma_PCC9432				
Ma_PCC9443				
Ma_PCC9701				
Ma_PCC9717				
Ma_PCC9806				
Ma_PCC9807				
Ma_PCC9808				
Ma_PCC9809				
Ma_QC_Ch_20071001_M135				
Ma_SC_T_19800800_S464				
Ma_Sj				
Ma_TAIHU98				
Mf_FACHB_1344				
Mf_QC_C_20070823_S20D				
Mf_WU_F_19750830_S460				
Mn_MB_F_20050700_S1D				
Mp_FACHB_1757				
Mp_MB_F_20051200_S9D				
Msp_0824				
Msp_LEGE00066				
Msp_MC19				
Msp_OC_L_20101000_S702				
Msp_T1_4				
Mv_FACHB_1342				
Mv_NIES_102	0.99729923			
Mw_FACHB_1317	0.99730003	0.99594463		
Mw_FACHB_1339	0.99865141	0.99729923	0.99594703	
Mw_MB_S_20031200_S109D	0.99730003	0.99594463	0.99594583	0.99865101

Table S5.

Proposed marker genes and primer sets for Sanger and Illumina sequencing. Proposed marker genes that can generate similar phylogenies to the core gene phylogeny shown in Fig. 2. See Fig S6 for the distribution of variable and conserved sites for marker genes.

Gene	Length (bp)	product	Ratio of variable to conserved sites§	Sanger sequencing		Illumina sequencing	
				Forward (5' → 3')	Reverse (5’→ 3')	Forward (5' → 3')	Reverse (5’→ 3')
acrB	3065	Multidrug efflux pump subunit AcrB	0.216	acrB_133: CCTTCTTTTAGTATCGATCAGAGAG	acrB_1305: TGGAGATTATCGGAGGCAATATA	acrB_605: GGTAGTTTACAGRTAAATACGGT	acrB_975: CCTAAAATTTGATCGTGAGTGGA
amtB	1436	Ammonia transporter	0.223	amtB_4: AACGAATTGCTCGAAAATCYGGGG	amtB_1280: TCTTCGGGAGTAACACGAATACCG	amtB_290: ATCGTTTTCGCCCTCTCCACCAT	amtB_610: ACCRGCGAAGTCYTTAAAACCAAG
cpoB	2168	Cell division coordinator CpoB	0.185	cpoB_60: GCSCTTTTTACTCTCACCAGTCTC	cpoB_1520: ACCGAGGTTAAATTGTTCGTCAACR	cpoB_785: CYGAACAAGCCTATCGTTACGC	cpoB_1090: AGCGATAATCGGCSGCTTCGT
dppA	1616	ABC transporter substrate-binding protein	0.250	dppA_225: GAAGGAACCACTAATTTAAAGCCC	dppA_1485: GGAACTTCATCRGCGAGAATTTC	dppA_225: GAAGGAACCACTAATTTAAAGCCC	dppA_490: GGRTAAGCTAAAAGAGCRGGAAA
glnA	1421	type I glutamate--ammonia ligase	0.161	glnA_1: ATGCCCGAAACGCCACAAGAAGTC	glnA_1352: CATCGGGTTAACTTCGTTATCGAG	glnA_812: ATGCACGTTCACCAGTCTATCTGG	glnA_1352: CATCGGGTTAACTTCGTTATCGAG
helY	2927	putative helicase HelY	0.178	helY_220: GCTCTTTCTAACCAAAAATTCCGGG	helY_1820: GGCAATTTTAGATGTATTTCCTGTC	helY_580: CCGACTGGATTAACTGGGTGCG	helY_835: GGAGCATATCCTTGTCCCGCAG
murC	1427	UDP-N-acetylmuramate--L-alanine ligase	0.174	murC_40: GGTATTGGCGGYATTGGGATGTC	murC_1065: CGTTGGTAYTGTTTGCCTTCTACC	murC_40: GGTATTGGCGGYATTGGGATGTC	murC_400: TAACCGATKARGCTGCTAGTGGTG
recJ	1964	single-stranded-DNA-specific exonuclease RecJ	0.179	recJ_15: TCCCATCCARCGCTGGTATATTGC	recJ_1455: ATTCCCCAGGGTTGYAAACTATCA	recJ_15: TCCCATCCARCGCTGGTATATTGC	recJ_360: ATTCCGTATCCCTCTTTCATGCGA
sbcC	3023	exonuclease subunit SbcC	0.207	sbcC_425: CAAGCGGACGARTTTATGAAACAG	sbcC_1900: AACGCCATCTTTTTTCTTCTCCTC	sbcC_425: CAAGCGGACGARTTTATGAAACAG	sbcC_771: GTCTAATCTCTCRATTTCYGCTAAT
trpE	1505	anthranilate synthase component I	0.148	trpE_90: ATTGCCGAGATCGCTAATCGGATGC	trpE_1290: CGCTGGCAAAATCGAGGCTATCAAAG	trpE_490: AAAGATTGCGAAACGGGAACCCCTAA	trpE_850: CCATAGTAACCCATGTGATCACCAAA
yccS	2237	FUSC-like inner membrane protein yccS	0.238	ycc_415: GTCGCTTTACAACGGGCCGGAAT	ycc_1660: TAGCTTCTAATGCTTTAATGGCAGC	ycc_1260: CCCATAGTTTTTGGATTGGTTTAACC	ycc_1660: TAGCTTCTAATGCTTTAATGGCAGC

§Based on alignment of 122 sequences of a marker gene using MUSCLE alignment tool with MEGA software *(44)*.

Table S6.

Cluster fidelity of the concatenated marker gene phylogeny. Correlation matrix between phylogenies based on concatenated marker genes and the original core gene phylogeny (Fig. 2).

Marker gene	*acrB*	*amtB*	*cpoB*	*dppA*	*glnA*	*helY*	*murC*	*recJ*	*sbcC*	*trpE*
acrB										
amtB	0.947									
cpoB	0.938	0.965								
dppA	0.982	0.965	0.956							
glnA	1.000	0.991	0.965	0.982						
helY	0.973	0.956	0.965	0.973	1.000					
murC	0.956	0.965	0.982	0.982	0.973	0.956				
recJ	0.938	0.947	0.903	0.920	0.929	0.938	0.920			
sbcC	0.965	1.000	0.965	0.982	0.956	1.000	0.982	0.956		
trpE	0.973	0.965	0.938	0.956	0.982	0.973	0.920	0.903	0.965	
yccS	0.956	0.982	0.973	0.982	0.982	0.973	0.982	0.947	0.982	0.956

Table S7.

Cluster identification of *Microcystis* in global lakes. Genospecies/cluster identified by phylogenies of marker genes extracted from MAGs from Lake Champlain (*14*) and from contigs assembled from metagenome data (other lakes, *3*).

	Genospecies																Groups						
Lake	A	B	C	D	E	F	G	H	I	J	K	L	M	N	O	P	5	7	8	18	19	20	21
Champlain												**X**	**X**		**X**	**X**					**X**		
Castle Rock							**X**									**X**							**X**
FP23																	**X**					**X**	
Grand lake																	**X**						
Pampulha				**X**			**X**																
Belső-tó																			**X**				
Münster																	**X**						
Villerest																							**X**
Kinneret					**X**													**X**					
Chaohu							**X**																
Clarendon		**X**																					
Rotoehu					**X**							**X**											
